# Comparative Phylodynamics Reveals the Evolutionary History of SARS-CoV-2 Emerging Variants in the Arabian Peninsula

**DOI:** 10.1093/ve/veac040

**Published:** 2022-05-18

**Authors:** Moh A Alkhamis, Nicholas M Fountain-Jones, Mohammad M Khajah, Mohammad Alghounaim, Salman K Al-Sabah

**Affiliations:** Department of Epidemiology and Biostatistics, Faculty of Public Health, Health Sciences Centre, Kuwait University, Kuwait City, Kuwait; School of Natural Sciences, University of Tasmania, Australia; Systems and Software development Department, Kuwait Institute for Scientific Research, Kuwait; Departement of pediatrics, Amiri Hospital, Ministry of Health, Kuwait; Jaber Al-Ahmad Al-Sabah Hospital, Ministry of Health, Kuwait; Jaber Al-Ahmad Al-Sabah Hospital, Ministry of Health, Kuwait; Department of Surgery, Faculty of Medicine, Health Sciences Center, Kuwait University, Kuwait

**Keywords:** SARS-CoV-2, variants of concern, phylodynamics, phylogeography, genomic surveillance

## Abstract

Emerging SARS-CoV-2 variants continue to be responsible for an unprecedented worldwide public health and economic catastrophe. Accurate understanding and comparison of global and regional evolutionary epidemiology of novel SARS-CoV-2 variants are critical to guide current and future interventions. Here we utilized a Bayesian phylodynamic pipeline to trace and compare the evolutionary dynamics, spatio-temporal origins and spread of and spread of five variants (Alpha, Beta, Delta, Kappa, and Eta) across the Arabian Peninsula. We found variant-specific signatures of evolution and spread that are likely linked to air travel and disease control interventions in the region. Alpha, Beta and Delta variants went through sequential periods of growth and decline, whereas we inferred inconclusive population growth patterns for the Kappa and Eta variants due to their sporadic introductions in the region. Non-pharmaceutical interventions imposed between mid-2020 and early 2021 likely played a role in reducing the epidemic progression of the Beta and the Alpha variants. In comparison, the combination of the non-pharmaceutical interventions and the rapid rollout of vaccination might have shaped Delta variant dynamics. We found that the Alpha and Beta variants were frequently introduced into the Arab peninsula between mid-2020 and early 2021 from Europe and Africa, respectively, while the Delta variant was frequently introduced between early and mid-2021 from East Asia. For these three variants, we also revealed significant and intense dispersal routes between the Arab region and Africa, Europe, Asia, and Oceania. In contrast, the restricted spread and stable effective population size of the Kappa and the Eta variants suggests that they no longer need to be targeted in genomic surveillance activities in the region. In contrast, the evolutionary characteristics of the Alpha, Beta and Delta variants confirms the dominance of these variants in the recent outbreaks. Our study highlights the urgent need to establish regional molecular surveillance programs to ensure effective decision making related to the allocation of intervention activities targeted toward the most relevant variants.

## Introduction

Severe acute respiratory syndrome coronavirus 2 (SARS-CoV-2), has caused unprecedented health and economic losses across the globe (Andersen et al., 2020). Within approximately 4 months from SARS-CoV-2 emergence in China in late 2019 (Zhu et al., 2020), it rapidly spread to almost every country, causing unprecedented rapid hospitalizations and mortalities on global scales (*World Health Organization (WHO) Coronavirus Disease (COVID-19) Dashboard. 2021. accessed [October 10th 2021] available at https://covid19.who.int*). Thus, on March 11th 2020, the World Health Organization (WHO) declared the new disease as a pandemic ("WHO Emergency Committee. Statement on the second meeting of the International Health Regulations (2005) Emergency Committee regarding the outbreak of novel coronavirus (COVID-19). Geneva: WHO, 2020. https://www.who.int/news-room/detail/30-01-2020-statement-on-the-second-meeting-of-the-international-health-regulations-(2005)-emergency-committee-regarding-the-outbreak-of-novel-coronavirus-(COVID-19) (accessed Feb 1, 2020).", 2020), which subsequently prompted governments to impose unparalleled public health restrictions that lasted for approximately two years. As of October 2021, over 240 million cases and 5 million deaths were reported in 223 countries or territories (*World Health Organization (WHO) Coronavirus Disease (COVID-19) Dashboard. 2021. accessed [October 10th 2021] available at https://covid19.who.int*). The SARS-CoV-2 is a enveloped single-stranded positive-sense RNA virus that causes the coronavirus disease 2019 (COVID-19) in humans (genus *Betacoronavirus*, family *Coronaviridae*). This family of viruses infect a wide variety of mammalian and avian species, and SARS-CoV-2 is the seventh coronavirus recognized to infect humans from potentially zoonotic origins (Andersen et al., 2020; Zhu et al., 2020). As of December 2021, RaTG13 and RmYN02 coronaviruses are the closest relatives to the SARS-CoV-2 (96% and 93% nucleotide similarity, respectively) and are commonly isolated from wild bats belonging to the species *Rhinolophus affinis* and *Rhinolophus malayanus* (Zhou et al., 2020).

The Gulf Cooperation Council (GCC) countries across the Arabian Peninsula have accumulated a total of 2.5 million confirmed cases and 19.5 thousand fatalities as of October 20th, 2021(*World Health Organization (WHO) Coronavirus Disease (COVID-19) Dashboard. 2021. accessed [October 10th 2021] available at https://covid19.who.int*). The first confirmed four cases of SARS-CoV-2 in the GCC region were detected in the United Arab Emirates on January 29th 2020, in a family of travelers arriving from Wuhan in central China (Nandkeolyar, 2020). Subsequently, Kuwait, Bahrain, Qatar, Oman and Saudi Arabia reported their first confirmed cases between mid-February and early March of 2020 (*World Health Organization (WHO) Coronavirus Disease (COVID-19) Dashboard. 2021. accessed [October 10th 2021] available at https://covid19.who.int*). Notably, patterns of SARS-COV-2 prevalence is heterogeneous among GCC member states. For example, between January 29th 2020 and October 20$^{th}$ 20201, the highest prevalence of SARS-CoV-2 cases was reported in Bahrain (%16.24), while the lowest was reported in Saudi Arabia (0.02%) (*World Health Organization (WHO) Coronavirus Disease (COVID-19) Dashboard. 2021. accessed [October 10th 2021] available at https://covid19.who.int*). Yet, such observed prevalence was highly correlated with country population size, number of daily tests and whether the countries reported both symptomatic and asymptomatic cases or only symptomatic patients. Furthermore, the mortality rate from the disease during the period, described above, was low (< 0.001%) in all GCC member states compared to the European countries such as Spain and Italy (*World Health Organization (WHO) Coronavirus Disease (COVID-19) Dashboard. 2021. accessed [October*

*10th 2021] available at https://covid19.who.int*). This low mortality rate from SARS-CoV-2 is mainly attributed to the well-funded healthcare system(Al Awaidy et al., 2021) and the region's past experience with the successful control and prevention efforts against Middle East Reparatory Syndrome (MERS-CoV) (Assiri et al., 2013).

Like many countries, the GCC member states imposed a series of strict non-pharmaceutical interventions throughout the pandemic period, including complete and partial lockdowns, international travel restrictions, public gatherings, contact tracing, and school closures (Hale et al., 2021). During the first wave, these non-pharmaceutical interventions were considered successful in reducing the number of cases, hospitalizations and deaths (*World Health Organization (WHO) Coronavirus Disease (COVID-19) Dashboard. 2021. accessed [October 10th 2021] available at https://covid19.who.int*). However, for the subsequent waves, which were dominated by emerging SARS-CoV-2 variants, non-pharmaceutical interventions were less effective in shaping the epidemic curves in the GCC member states. Declines in cases, hospitalization and mortalities only occurred when most GCC countries reached a vaccination coverage of greater than 40% (*World Health Organization (WHO) Coronavirus Disease (COVID-19) Dashboard. 2021. accessed [October 10th 2021] available at https://covid19.who.int*).

After the first global wave of the pandemic, new variants of the SARS-CoV-2, characterized by increased transmissibility and a large number of mutation and deletions rates on the spike (S) protein, began to rapidly emerge and spread within a short time in different continents (Gomez et al., 2021). These variants are classified differently by the major repositories and organizations; Global Initiative on Sharing All Influenza Data (GISAID; gisaid.org), NextStrain (nextstrain.org), and Pangolin (cov-lineages.org). However, the WHO reclassified SARS-CoV-2 emerging variants into three categories, namely Variants Of Concern (VOC), Variant Of Interest (VOI), and Variants Under Monitoring (VUM) ("World Health Organization (WHO) Tacking SARS-CoV-2 Variants. 2021. accessed [December 20th 2021] available at https://www.who.int/en/activities/tracking-SARS-CoV-2-variants/," 2021). As of October 2021, VOCs includes Alpha (B.1.1.7, GRY, or 20I), Beta (B.1.351, GH/501Y.V2, or 20H), Gamma (P.1, GR/501YV3, or 20J), and Delta (B.1.617.2, or 21A, 21I, and 21J), while VOIs includes Lambda (C.37, GR/452Q.V1, or 21G) and Mu. Additionally, VUMs includes Kappa (B.1.617.1, G/452R/V3, or 21B) , Iota (B.1.526, GH/253.V1), Eta (B.1.525, G/484K.V3, or 20A/S484K) and Epsilon (B.1.427/B.1.429, GH/452R/V1, or 20C/S.452R) ("World Health Organization (WHO) Tacking SARS-CoV-2 Variants. 2021. accessed [December 20th 2021] available at https://www.who.int/en/activities/tracking-SARS-CoV-2-variants/," 2021). The WHO issued guidance for each designated variant category containing recommended surveillance activities and primary action measures ("World Health Organization (WHO) Tacking SARS-CoV-2 Variants. 2021. accessed [December 20th 2021] available at https://www.who.int/en/activities/tracking-SARS-CoV-2-variants/," 2021). We adapted the nomenclature proposed by the WHO throughout the study to maintain its clarity and consistency.

Many countries have implemented SARS-CoV-2 whole-genome sequencing programs as a genetic surveillance tool throughout the pandemic, including the GCC member states (Al-Mahruqi et al., 2021; Benslimane et al., 2021; Bindayna et al., 2021; Liu et al., 2021; Mourier et al., 2022; Tayoun et al., 2020). However, countries that implemented integrated phylodynamic methods have uncovered important epidemiological insights at local, regional, and global scales (Benvenuto et al., 2020; Fountain-Jones et al., 2020; Worobey et al., 2020). For example,

Bayesian phylodynamic methods have been used as a robust tool for investigating circulating variants' evolutionary epidemiology by tracing their origins(Butera et al., 2021; Giovanetti et al., 2020; Nabil et al., 2020), quantifying transmission events (Miller et al., 2020; Seemann et al., 2020), and assessing the impact of non-pharmaceutical interventions before and after their implementation (Geoghegan et al., 2020; López et al., 2021). However, phylodynamic and biogeographic methods have not been fully utilized in the GCC member states, with the exception of Saudi Arabia(Mourier et al., 2022), as well as in other Asian countries (Farah et al., 2020; Jeewandara et al., 2021). This represents an important gap not only for local COVID-19 surveillance but also as the region is a major travel hub between Europe and Asia (Tayoun et al., 2020). Between November 16th 2020 and June 28th 2021, Alpha, Beta, Delta, Kappa, and Eta were the most prevalent WHO designated variants in the GCC member states, but the epidemiological dynamics of these variants is largely unknown in the region. Utilized in a unified Bayesian framework (Grubaugh et al., 2019; Pybus & Rambaut, 2009), these methods have the capacity to model viral evolution and spread in time and space to untangle epidemiological patterns and variant dynamics.

Between November and December 2021, the Omicron variant (B.1.1.529, or GRA) emerged and rapidly spread to over 70 countries worldwide ("World Health Organization (WHO) Tacking SARS-CoV-2 Variants. 2021. accessed [December 20th 2021] available at https://www.who.int/en/activities/tracking-SARS-CoV-2-variants/," 2021). Thus, a comparative understanding of global and regional evolutionary epidemiology of SARS-CoV-2 rapidly emerging variants are critical to guide current and future interventions. Here, we adapt a Bayesian phylodynamic pipeline to trace and compare evolutionary history, spatio-temporal origins and transmission dynamics of the five variants described above. We uncover new comparative insights into the evolutionary characteristics of SARS-CoV-2 selected variants and their spread from and into the GCC member states. These findings may improve current genetic surveillance efforts on regional scales and subsequently reduce the impacts of current and future emerging variants.

## Methods

### *Preliminary phylogenetic analysis*

We retrieved 3728 non-identical complete genome SARS-CoV-2 sequences from the GISAID (file S1) isolated from cases reported in the GCC member states between January 28th 2020 and June 28th 2021. We excluded identical sequences from the analysis, as they usually have a very low prevalence in full genome datasets. Also, 100% identical sequences usually do not improve the temporal signal of the phylogeny, lead to improper convergence of the posterior estimates, and may substantially increase the computational costs (Lycett et al., 2021; Morel et al., 2021). Approximately 81.5% (n = 3041; file S1) of the GCC sequences contained earlier lineages such as A, B, C and G. While approximately 18.5 % (686; Table S1) of the published sequences contained WHO designated variants, which comprised the Alpha, Beta, Delta, Kappa, and Eta. Next, we downloaded the metadata of the sequences used to construct the Novel Coronavirus-Global Subsampling (n-cov) tree from the NextStrain web interface (https://nextstrain.org/ncov/gisaid/global) in order the retrieve the corresponding sequences from the GISAID database (file S2 for the accession numbers). The NextStrain sequence dataset comprised 2971 globally representative sequences collected between December 26th 2019, and July 1st 2021. Here, we adapted the terminology suggested by NextStrain documentation (Hadfield et al., 2018), and designated the GCC dataset as focal sequences (i.e., sequences obtained locally), while the NextStrain global dataset as context sequences (i.e., sequences obtained from global public repository). The NextStrain algorithm randomly subsamples approximately 600 sequence predefined continental regions, with approximately 400 from the previous 4 months and approximately 200 from earlier strains, to generate representative inferences that are computationally efficient for the average users (see https://nextstrain.org/ncov/gisaid/global). Since countries like Denmark have substantially surpassed other regions in terms of the number of sequences isolates (see gisaid.org), We chose the NextStrain genomic dataset as our context sequences because they ensure a more equitable representation of sequence distribution across different continental regions.

To reduce the computational intensity of the subsequent analyses, we used genome-sampler version 2.0 (Bolyen et al., 2020) to subsample the context sequences while maintaining their representation in terms of the time of isolation, location of isolation, and genetic diversity (Bolyen et al., 2020). This procedure excluded 1781 sequences (59.9%) of the context sequences, and therefore, the final combined dataset (*i.e.,* focal and context) comprised 4918 sequences. We used MAFFT version 7.49 (Katoh & Standley, 2013) to align our sequence dataset and trimmed the first 130bp and last 50bp from the final alignment, as suggested elsewhere, using AliView version 1.74 (Larsson, 2014). We found no recombination events in the final alignment using Recombination Detection Program version 4.0 (Martin et al., 2015). We then used IQ-tree version 2.0 (Minh et al., 2020) to construct a Maximum Likelihood (ML) tree with 1000 ultrafast bootstraps. Using the inbuilt model selection algorithm implemented in IQ-Tree (Kalyaanamoorthy et al., 2017), we found the general time-reversible model with empirical base frequencies and gamma-distributed varying rates with four rate categories (GTR+F+R4) was the best substitution model fitting the data.

We then extracted the sequences from each WHO designated variant (*i.e.,* Alpha, Beta, Delta, Kappa, and Eta; Figure 1) from the ML tree and constructed an independent ML tree for each variant. We used the root-to-tip regression procedure implemented in TempEst version 1.5.3 (Rambaut et al., 2016) to confirm the presence of significant temporal in the phylogeny of each variant. We found that all variants' sequence datasets exhibited significant temporal signals ($R^2 > 0.2$; Figure 1), suitable for the subsequent molecular clock analyses. Furthermore, we also used TempEst to investigate additional sequence error and outliers within the dataset of each variant. Additionally, we used TreeTime (Sagulenko et al., 2018) to calibrate the phylogeny (not shown) of each variant in time. We used two genomes from Wuhan, 2019 (*i.e.,* hCoV-19/Wuhan/WH01/2019, and hCoV-19/Wuhan/Hu-1/2019) as an outgroup to estimate the posterior parameters of the subsequent molecular clock analyses.

*Variant demographics and phylogeography*

We reconstructed the demographic and phylogeographic history of SARS-CoV-2 selected variants using the relaxed-molecular clock models implemented in BEAST 1.10.4 (Suchard et al., 2018) and the BEAGLE library (Ayres et al., 2019) to improve the computational efficiency of the subsequent phylodynamic models. We used the substitution model described above and assessed three parametric (*i.e.,* constant population size (Kingman, 1982), exponential (Griffiths & Tavare, 1994), and expansion growth(Griffiths & Tavare, 1994)) one non-parametric (*i.e.,* Bayesian Skygrid (Gill et al., 2013)) coalescent node-age tree priors. Also, we evaluated the uncorrelated exponential and the lognormal branch-rate priors in combination with each node-age model. Additionally, we compared eight candidate models using the Alpha and Delta sequence datasets to determine the best fitting demographic model for other variants in the subsequent analyses, using the Bayes factor (BF) comparison of the marginal-likelihood estimates calculated using path-sampling (PS) and stepping-stone (SS) methods(Baele et al., 2012).

We quantified the global origins of SARS-CoV-2 selected variants and identified significant dispersal routes between the GCC and other geographical regions (*i.e.,* continents) using the discrete Bayesian phylogeographic analysis extension implemented in BEAST (Lemey et al., 2009). We aggregated the GCC countries into a single geographical location and specified a total of seven discrete traits, including Africa, Asia, Europe, GCC, Oceania, North and South America (Table S2). However, for each variant, continents with sequences less than two were excluded from the discrete trait assignment to limit the impact of biased posterior inferences related to the phylogeographic analysis (De Maio et al., 2015). For each variant, we identified significant dispersal routes and their directionality between regions using the Bayesian stochastic search variable selection (BSSVS) approach (Pybus & Rambaut, 2009). Also, we explored the fit of each sequence dataset to the asymmetric model (irreversible transitions) and the symmetric model (reversible transitions) using the BF comparisons, as described above. Further, we used the Markov-jump (MJ) procedure(Farr et al., 2015) to infer the expected number of backward and forward transitions between the GCC and other regions.

We performed our phylodynamic analysis in duplicate and ran the Markov chain Monte Carlo (MCMC) simulations for 350 million iterations sampling every 30,000 steps for the Alpha, Beta, and Delta variants. However, due to the small sample sizes of the Kappa and Eta datasets, we performed 100 million MCMC cycles while sampling every 10,000 steps. We used Tracer version 1.7.1(Rambaut et al., 2018) to monitor the proper convergence of the posterior parameters by reaching the effective sample size (*i.e.,* ESS > 200) We discarded the first 10% of the sampled trees from each MCMC chain and generated the maximum clade credible (MCC) tree based on the median node heights using TreeAnotator version 1.10.4.

To estimate effective population size of each variant through time, we used the Skygrid coalescent tree with the uncorrelated lognormal branch-rate models. We used FigTree version 1.4.4(Rambaut, 2018) to plot the resulting phylogeographic MCC tree for each variant. We estimated the effective population size for all sequences combined as well as for a clades circulating predominantly in the GCC. We identified these clades based on the root-state posterior probability inferred from the phylogeographic reconstructions that represents the earliest introduction into the GCC region. Clades introduced to the GCC that had > 5% of the sequences from other countries were excluded. We did not use this approach on the Kappa and the Eta variant due to the extremely low number of sequences isolated in the GCC (table S1). Then, we used Tracer to generate the Skygrid plots for each variant as well as for each identified dominant GCC clade. Further, we used SPREAD3(Bielejec et al., 2016) version 0.9.6 to assess the significance of the non-zero rate dispersal routes using the implemented BSSVS BF procedure (BSSVS BFs > 3). We plotted significant dispersal routes between regions using the JavaScript Object Notation (JSON) maps and SPREAD3. Finally, we used the Bayesian Tip-association significance test(Parker et al., 2008) implemented in BaTs version 2.0 to validate the association between the posterior phylogeny and spatial discrete traits. We used the association index (AI) and parsimony score (PS) computed from a sample of 1000 posterior trees and a 100 null replicated to assess whether the sampling regions contributed significantly to the structure of the inferred MCC tree for each variant.

**Results**

*Demographic history and pandemic dynamics*

The results of the BF comparisons indicated that the combination of the expansion growth node-age and the uncorrelated exponential branch-rate priors are the best fitting model for the Alpha and Delta variants (BFs > 50 and 3, respectively; Tables S3 and S4), and therefore was used to infer the evolutionary history of other variants. The highest estimate of the mean nucleotide substitution rate per year was inferred for Delta variant ($1.12x10^{-3}$, 95% HPD [$9.71x10^{-4}$, $1.38x10^{-3}$]; Table 1), while the lowest estimate was inferred for the Alpha variant ($2.60x10^{-4}$, 95% HPD [$1.63x10^{-4}$, $3.70x10^{-4}$]; Table 1). Our demographic reconstructions revealed sequential peaks of effective population size for Alpha, Beta and Delta variants (Figure 2).

**Table 1. Inferred substitution per site per year for the selected SARS-CoV-2 variants in the GCC countries between November 16th 2020 and June 28th 2021**.

| Variant | Substitution/site/year | 95% HPD* |
|---|---|---|
| Alpha | $2.60x10^{-4}$ | [$1.63x10^{-4}$, $3.70x10^{-4}$] |
| Beta | $7.0x10^{-4}$ | [$5.32x10^{-4}$, $8.68x10^{-4}$] |
| Delta | $1.12x10^{-3}$ | [$9.71x10^{-4}$, $1.38x10^{-3}$] |
| Eta | $5.07x10^{-4}$ | [$4.81x10^{-4}$, $8.78x10^{-4}$] |
| Kappa | $6.49x10^{-4}$ | [$4.63x10^{-4}$, $9.09x10^{-4}$] |

Our BF comparisons revealed that the asymmetric phylogeographic model with irreversible transitions was the best fitting model for the Alpha and the Delta variants. In contrast, the symmetric phylogeographic model with reversible transitions was the best fitting model for the Beta, Kappa, and the Eta variants (BFs > 15). Further, all of the estimated AI indices and PS scores of the selected geographic discrete traits were statistically significant among all variants (Table 2).

Our combined phylogeographic and demographic reconstructions revealed sequential variant waves with divergent phylogeographic and demographic histories (Figure 2). Our regional phylogenetic analysis suggests that the introductions of the Alpha variant into the GCC originated from Europe (RSPP = 0.77), with strong posterior support, during July 2020 (95% HPD [May 2020, August 2020]; (Figure 2A). Since the Alpha variant was introduced to the GCC, our demographic reconstruction of the dominant GCC clade showed plateauing effective populations size after September 2020 followed by a decline in January 2021 (Figure 2B). In contrast, our results showed that the introductions of the Beta and the Delta variants originated from Africa (RSPP = 0.76) in June 2020 (95% HPD [April 2020, September 2020]; Figure 2D) and East Asia (RSPP = 0.98) in January 2021 (95% HPD [December 2020, February 2021]; Figure 2G). Our demographic reconstruction of the selected Beta clade dominant in the GCC post introduction showed two distinct peaks in January and May 2021 followed by notable declines of the effective populations (Figure 2D). However, the effective population size of the Delta clade rapidly peaked and declined in June 2021 onward (Figure 2F). All of the selected GCC clades partially mirrored our complete demographic construction (i.e., when sequences from all countries included, Figure S 2A, 2B, & 2C). Results of the mixed global and local demographic reconstructions for the effective population size through time for the five selected variants are illustrated in Figure S2. We found some support for the Kappa and the Eta variants originating from Oceania (RSPP = 0.71) in January 2021 (95% HPD [November 2020, May 2021] and Europe (RSPP = 0.70) in February (95% HPD [August 2020, April 2021]), respectively, in which both of their estimated posterior statistical supports were substantially low ($P < 0.5$; Figures 2G & 2H). As the number of sequences from these variants was low (Tables S1 & S2), we were unable to reconstruct the demographic history of these variants within the GCC. However, we did detect substantially low effective population sizes for both the Kappa (Figure S2D) and the Eta (Figure S2E) variants with little sign of decline.

The BSSVS approach suggested that the most significant unidirectional dispersal route inferred for the Alpha variant was from Europe to the GCC ($BF > 100$; Figure 3A), while the least significant dispersal route was inferred from Asia to the GCC ($BF < 100$; Figure 3A). Additionally, Europe had the highest mean counts of relative forward transitions into the GCC (Forward = 32 *vs* Reverse = 10; Figure 3A). Results of the Beta variant suggest that the most significant bidirectional viral dispersal routes were between the GCC and Africa ($BFs > 100$; Figure 3B), followed by Asia ($BF < 100$; Figure 3B). However, both Africa and Asia had notably higher forward than reverse transitions ($MJs \geq 4$) in terms of viral jumps into the GCC (Figure 3B). We found four significant unidirectional dispersal routes for the Delta variant (Figure 3C), in which the most significant routes ($BFs > 100$) were from the GCC to Asia and Oceania (Figure 4C). However, the relative forward transitions from Asia and Oceania were substantially higher than those from the GCC ($MJs > 25$; Figure 3C). Further, the least significant dispersal routes inferred for the Delta variant were from the GCC to Africa and from North America to the GCC ($BFs < 100$; Figure 3C). Overall, we inferred least significant dispersal routes ($BF < 100$) and low mean counts of viral jumps for the Kappa and the Eta variants (Figures. 3D & 3E). However, for both the Kappa and the Eta variants the mean count of forward transitions from different global regions into the GCC was substantially higher than from reverse transitions from the GCC (Figures. 3D & 3E).

**Table 2. Association indices and parsimony scores for the discrete trait phylogeographic model of each selected variant.**

| Variant | | AI[1] | 95% CI | P-value | PS[2] | 95% CI | p-value |
|---|---|---|---|---|---|---|---|
| Alpha | Observed | 12.1 | (10.4, 13.7) | <0.001 | 123.4 | (117.0, 129.0) | <0.001 |
| | Null | 45.5 | (43.7, 47.8) | | 282.4 | (275.5, 289.9) | |
| Beta | Observed | 3.4 | (2.6, 4.2) | < 0.001 | 38.0 | (36.0, 40.0) | < 0.001 |
| | Null | 19.7 | (18.5, 21.0) | | 103.8 | (101.4, 106.2) | |
| Delta | Observed | 8.0 | (6.7, 9.3) | 0.009 | 90.9 | (86.0, 96.0) | < 0.001 |
| | Null | 31.3 | (29.8, 32.8) | | 178.9 | (174.7, 182.6) | |
| Kappa | Observed | 1.3 | (1.0, 1.7) | 0.004 | 9.7 | (9.0, 11.0) | 0.005 |
| | Null | 2.2 | (1.7, 2.7) | | 15.0 | (13.3, 16.5) | |
| Eta | Observed | 0.5 | (0.1, 0.9) | < 0.001 | 6.1 | (5.0, 7.0) | 0.009 |
| | Null | 2.6 | (2.1, 3.1) | | 14.3 | (13.5, 14.9) | |

[1]Association Index (AI); [2]Parsimony Score (PS)

## Discussion

We used a rigorous comparative Bayesian phylodynamic statistical framework to untangle the evolutionary epidemiology of five SARS-CoV-2 variants in the GCC member states. We shed new insights into each variant's demographic history in parallel with the epidemic progression and geographic origins by quantifying their demographic history and migration routes between the GCC and other continents. Thus, our results represent a mixture of global and local demographic and phylogeographic reconstructions with special emphasis on the evolutionary characteristics of GCC viruses. These findings are not only critical for guiding current and future SARS-CoV-2 molecular surveillance programs globally and in the GCC but assist with the intervention efforts on this public health devastating pathogen.

Our phylogeographic analysis revealed that the Alpha, Beta, and Delta epidemic waves in the GCC member states resulted from multiple direct introductions from regions where the viruses were first identified (i.e., Europe, Africa, and East Asia, respectively; Figures 2A, 2C, & 2E) (Galloway et al., 2021; Kirola, 2021; Mwenda et al., 2021; Yadav et al., 2021). Our findings support the notion that the evolution of SARS-CoV-2 emerging variants is strongly structured and maintained by their geographic origins (Hassan et al., 2021). Also, our results demonstrate that the increased number of reported cases in the regions of origins precipitates the introduction of each variant into the GCC region (Figures 2A, 2C, & 2E). For the Alpha variant, we found significant and intense (BF-BSSVS > 100, MJs > 30) unidirectional dispersal routes from Europe into the GCC region (Figure 3A). In contrast, weakest and least supported dispersal routes were from Asia in the GCC (Figure 3A). These findings are likely to reflect the effect of partial travel restrictions imposed by the GCC countries, in which citizens were allowed to return immediately to their countries while limiting the entries of expatriates and migrant workers (Hale et al., 2021). There is a well-established preference for GCC citizens to travel to Europe for tourism, study, and medical care, and therefore, they may have been vectors of Alpha dispersal into the GCC when they were allowed to return. However, our analysis revealed significant bidirectional, although notably less intense, migration routes for the Beta variant between the GCC and Africa and Asia (Figure 3B). This regional circulation might reflect the heterogeneous implementation of the travel restrictions across the GCC region. For example, Qatar(Benslimane et al., 2021) and UAE(Liu et al., 2021) both with major international airports, allowed limited commercial flights mainly for foreign travelers throughout the second half of 2020 (Hale et al., 2021). Interestingly, our Delta variant results unveiled significant unidirectional dispersal from the GCC into Asia and Oceania, but the forward viral jumps from both regions were more intense than the GCC (Figure 3C). The easing of non-pharmaceutical interventions in March and April 2021 may have facilitated the circulation and maintenance of the Delta variant in the region. The easing of movement restrictions for migrant workers may have been a driver of this pattern as they constitute a substantial portion of the total population in the GCC ( *International Labour Organization. "Labour Migration (Arab States)". International Labour Organization. Accessed [November 15th 2021] available at https://www.ilo.org/beirut/areasofwork/labour-migration/lang--en/index.htm.*, 2021). Migrant workers in the GCC largely originate from East Asian countries such as India, Bangladesh, and Nepal, and the circulation between Asia and the GCC may be explained by the increased movement of these workers between countries after restrictions were lifted. The paucity of migration routes detected for the Kappa and Eta variants (Figures. 3D & 3E) highlight the reduced importance of these variants in the region. This finding is in line with what is known globally about the biology of these variants of interest. For example, the Kappa variant is known not to be as transmissible as Delta, and Eta is only found at low prevalence (Tao et al., 2021).Yet, the low prevalence of the Kappa and the Eta variants in the GCC (Table S1 & S2) might significantly affected their posterior inferences.

Moreover, our phylogeographic results were used to inform our demographic analyses to help isolate the phylodynamic patterns at a regional scale. The robustness of the our Bayesian Skygrid analysis was reflected by demonstrating how the effective population size of each targeted clade in the GCC was similar to the overall population size (Figures 2B, 2D & 2F, Figures S 2A, 2B, 2C). Further, we found a similar declining pattern after an increase in the population sizes of the GCC Alpha and the Beta sclades that coincided with the temporal pattern of the reported cases (Figure S1). Yet, a second increase in effective population sizefor the Beta variant in March and April 2021 (Figure 2D) coincided with anincrease in the number of sequenced isolates. This finding suggests the introduction of a second wave of Beta strains in the region. However, the effective population size of the Delta clade predominantly in the GCC remained high for approximately 4 months with notable decline by the end of the study period (Figure 2F) mirroring the temporal pattern of the overall reported cases in the region (Figure S1). These findings may indicate that the Delta variant was introduced into the GCC at later stages of the global Delta wave, since its emergence in East Asia in late 2020 ("World Health Organization (WHO) Tacking SARS-CoV-2 Variants. 2021. accessed [December 20th 2021] available at https://www.who.int/en/activities/tracking-SARS-CoV-2-variants/," 2021). This is not surprising, since most commercial flights between the GCC and East Asia were mostly restricted in early 2021, while the first sequenced isolates were reported in early March 2021 (GISAID.org) throughout the region in parallel to the beginning of easing such restrictions. Thus, given the high mutation rate of the Delta variant, the continued genomic surveillance should either detect persistent decline in the genetic diversity of the circulating Delta viruses or introduction of further strains that could reaggravate the genetic diversity as inferred with the Beta variant (Figure 2D). Such findings justify the allocation of additional resources for targeted genomic surveillance of the Delta variant over other selected variants during this specific period of time.

Our results also show the potential impact of pharmaceutical and non-pharmaceutical interventions on variant dynamics. Most GCC member states relaxed their non-pharmaceutical interventions at the beginning of 2021 following the large-scale rollout of vaccinations (Hale et al., 2021). At this time, our models show a rapid decline in the population sizes for Beta and Delta variants that may be attributed to this vaccination rollout (Figures 2D & 2F). However, the slow decline of the genetic diversity of the Alpha variant in late January 2021 maybe attributed to the strict implementation of the non-pharmaceutical interventions and natural immunity resulting from the highly observed recovery rate in the region (Kojima & Klausner, 2021; López et al., 2021). These findings are almost in agreement with the observed epidemic curve in the GCC region (Figure S1). It is worth noting that the waves dominated by the Beta and the Delta variant had coincided with an unusual, prolonged sandstorm that occurred during mid-March 2021(Meo et al., 2021) and lasted for approximately two weeks throughout the GCC region. This sandstorm resulted in a substantial increase in the air Particle Matter (PM) 2.5, subsequently leading to a sharp increase in the number of cases throughout the region (Figure S1). Also, it is worth that noting shorter duration (*i.e.,* 2-4 days long) sand storms are frequent in the region between April and May of each year (Arishi, 2021), and therefore, the air pollution accompanied by related anthropological behaviours could have aggravated and intensified the prolonged population growth of the local Delta variant (Figure 2F), and subsequently, its transmissibility in the communities (Baron, 2021). Furthermore, the epidemic curve of the GCC had two prominent peaks between May and July for both 2020 and 2021 (Figure S1), suggesting hints of seasonality in the transmission of the virus in the region (Fountain-Jones et al., 2020). Therefore, just like other respiratory diseases, air pollution and seasonality of the SARS-CoV-2 could be considered in future studies using generalized linear (GLM) model-based phylodynamic methods (Tobias et al., 2021) (Lemey et al., 2014).

Our study has limitations typically found in phylogenetic studies derived from publicly available genomic surveillance data. One important limitation is that our phylodynamic analyses used only sequences available at GISAID within the time frame of the present study. This restriction resulted in some biases in space and time for each variant. For example, Saudi Arabia and Oman were severely underrepresented in terms of the number of sequences and variants detected (see Figure 1 and Table S1). Furthermore, the availability of sequence metadata mainly related to travel history was extremely rare. Thus, such limitation prevented us from implementing the recently introduced phylodynamic method that accommodates individual travel history to generate less biased and more realistic posterior inferences (Lemey et al., 2020). Also, the notable inflation in the uncertainty estimates of substitution per site per year for each variant (Table 1) might be attributed to short time frame of the sampling events (Figure S2) (Ghafari et al., 2022). That said, our phylodynamic inferences were based on all available genomic data collected by passive and active surveillance in the GCC region. Furthermore, our spatial discrete trait aggregation of the GCC countries into one unit might be helped in reducing the biases caused by missing information. For example, with the exception of the Beta variant, the demographic reconstruction results showed an early prolonged slow increase in the effective population size rather than a sharp increase even though most isolates were collected in a later stage from the study period (Figures S2 A, C, D, &E; Figures 2B & 2F). Additionally, consistent declines in the population size were inferred, particularly for the Alpha, and the Delta variants, which were also not affected by the later intensity of the sampling events (Figures 2B & 2F). Further, for the Alpha, Beta, and Delta variants, we inferred the region from which they were first isolated, as the ancestral location of introduction, while the GCC sequences' number was substantially larger (Table S2).

Saudi Arabia was the first country in the GCC that integrated phylodynamic methods in their genomic surveillance of SARS-CoV-2(Mourier et al., 2022). However, their implementation focused on identifying mutations in the nucleocapsid (N) protein, followed by reconstructing population demographics and epidemic progression of earlier lineages (Mourier et al., 2022). Therefore, there were no rigorous attempts related to the direct implementation of phylogeographic reconstructions of multiple emerging variants in the region to the authors' knowledge. It is worth noting that revisiting the best fitting model for the selected variants is critical when a new sequence dataset is made available, especially when sampling events are done over shorter time durations. This is because the evolutionary parameters of rapidly evolving pathogens like SARS-CoV-2 are time-dependent, in which best fitting node-age or/and branch rate might change over time with new sequence datasets, leading to different inferred posterior estimates (Ghafari et al., 2022). Furthermore, our analysis pipeline is focused on the comparative evolutionary characteristics of emerging variants to guide risk-based genomic surveillance efforts. Thus, in our GCC scenario, future genomic efforts should target the Delta variant and compare its evolutionary characteristics to the newly emerging variants, such as the Omicron variant. Also, if one decides to analyze multiple partial gene segments simultaneously, it is important to note that each gene segment might require different node-age or/and branch-rate model combinations, which might lead to similar or different posterior results.

## Conclusions

Our study represents the first attempt in the GCC region and the Middle East to implement a rigorous Bayesian phylodynamic analytical pipeline to compare and target the evolutionary characteristics of emerging variants of SARS-CoV-2. We unveiled and compared the evolutionary demographics and phylogeographic history of the Alpha, Beta, Delta, Kappa, and Eta variants under pharmaceutical and none-pharmaceutical interventions in the GCC region. Our inferred phylodynamics for the Kappa and the Eta variants suggests that they no longer need to be targeted in genomic surveillance activities in the GCC. In contrast, the evolutionary characteristics of the Alpha, Beta and Delta variants confirms their dominance in the GCC in the 2021 outbreaks, and therefore need to be targeted, particularly the Delta variant. Due to the continued rapid evolution of SARS-CoV-2 and the emergence of new variants, increasing sequencing capacity and detailed reporting of their related metadata, particularly in undersampled geographical areas, is critical for improving the performance and accuracy of current genomic surveillance programs in the region. Therefore, our study highlights the urgent need to establish collaborative regional molecular surveillance programs to ensure effective decision making related to the allocation of intervention activities targeted toward the most relevant variants.

**Acknowledgment**

This research was funded by part by the Kuwait Foundation for the Advancement of Science (Project No: 2020/1428). Accession numbers and their corresponding acknowledgment information for focal and context sequences in s1.focal.sequences.pdf and s2.context.sequences.pdf, respectively, in accordance with GISAID regulations.

**Data availability**

BEAST XML files and R-codes for the analysis of each variant are available at:
https://github.com/maalkhamis/Comparative-Phylodynamic-Models-for-SARS-CoV-2-in-the-GCC

**Figure Captions**

**Figure 1. Reconstructed maximum likelihood (ML; n = 4918) tree and the selected variants' prevalence among the Arabian Peninsula countries of SARS-CoV-2 between November 2020, and June 2021.** (A) The ML tree consisted of all SARS-CoV-2 variants identified worldwide between December 26th 2019, and June 28th 2021and was reconstructed using the GTR+F+R4 substitution model, and 1000 bootstrap replicates implemented in IQ-Tree version 2. The scale bar below the tree indicates the substitution rate per site. The five selected variants are highlighted by distinct colours, and their root-to-tip divergence was estimated using TempEst version 1.5.3. (B) Pie charts represent the prevalence of the selected variants per GCC country, in which the size of the pies is proportional to the number of sequences published in www.gisaid.org. The maps were generated using Q-GIS version 3.16.

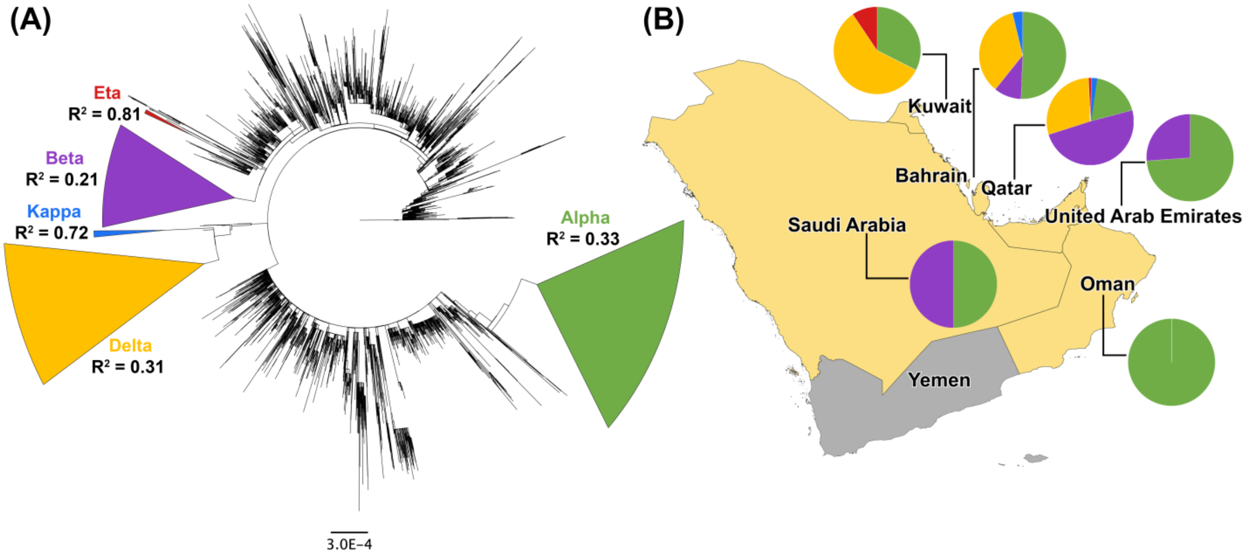

**Figure 2. Maximum clade credibility (MCC) phylogeny of the SARS-CoV-2 variants in the GCC countries (with related global isolates) between November 2020 and June 2021 and their demographic reconstruction of selected early introductions (highlighted in red rectangles) in the Arabian Peninsula countries.** (A, C, E, G, & H) Color of the branches indicate the most probable geographical location state of their descendent nodes and corresponds to the legend on the upper left of each tree. Circle sizes are proportional to the posterior node support (P > 0.5 for larger circles and P < 0.5 for smaller circles). The red arrows point to the earliest probable introduction of the variant to the GCC countries from other regions with their inferred root state posterior probability (RSPP). The panels on the right (B, D, & F) represent Bayesian skygrid plots of the effective population size through time for the selected GCC clades within the phylogeny of the Alpha, Beta, and the Delta variants. The posterior median estimate is indicated by the dark colored line, and the light shaded areas correspond to the 95% high posterior density (HPD). The vertical doted lines corresponds to the estimated time at which each variant transitioned from slow to fast population growth. (A-B) Alpha variant; (C-D) Beta variant; (E-F) represents Delta variant (G) represents Kappa variant; (H) represents Eta variant.

Alpha
Africa
Asia
Europe
GCC
North America
Oceania
South America
RSPP = 0.77
(A)
2019-07
2020-01
2020-07
2021-01
Effective Population Size (NeT)
Number of Sequences
Time (Years)
2020-08
2020-11
2021-02
2021-05
(B)
Beta
RSPP = 0.76
(C)
(D)
2020-10
2020-12
2021-02
2021-04
2021-06
Delta
RSSP = 0.98
(E)
2021-07
(F)
2021-03
2021-05
Kappa
RSPP = 0.71
(G)
Eta
RSPP = 0.70
(H)
Time (years)

RIPT

**Figure 3**. **Regional exchange routes and posterior mean counts of the Markov jumps (MJ) over the phylogeny of the selected SARS-CoV-2 variants in the GCC countries between November 2020 and June 2021.** Dispersal routes with none-zero rates were inferred using the Bayesian stochastic search variable selection (BSSVS) and were selected using Bayes factor comparisons. Significantly supported Dispersal routes (Bayes Factor > 3) are plotted. Red arrows represent dispersal routes with the strongest posterior support (BSSVS BF > 100) as indicated by the legend on the lower left. The bar chart on the right of each map summarizes the total expected forward and reverse MJs between the GCC countries and other regions. (A) represents Alpha and was inferred from the asymmetric phylogeographic model with irreversible transitions; (B) represents Beta and was inferred from the symmetric phylogeographic model with reversible transitions; (C) represents Delta and was inferred from the asymmetric phylogeographic model with irreversible transitions; (D) represents Kappa and was inferred from the symmetric phylogeographic model with reversible transitions; (E) represents Eta and was inferred from the symmetric phylogeographic model with reversible transitions. (F) GCC countries' names. The maps were generated using by Q-GIS version 3.16 and SPREAD3 version 0.96.

## Supplementary Material

veac040_Supp

