# Supplementary material for: Comparative Phylodynamics Reveals the Evolutionary History of SARS-CoV-2 Emerging Variants in the Arabian Peninsula
Source: Virus Evol. 2022 May 18:veac040. doi: 10.1093/ve/veac040 (PMC9129158; doi:10.1093/ve/veac040)
Supplement: veac040_Supp [file veac040_supp.zip › s1.focal.sequences.pdf]

We gratefully acknowledge the following Authors from the Originating laboratories responsible for obtaining the specimens, as well as the Submitting laboratories where the genome data were generated and shared via GISAID, on which this research is based.

All Submitters of data may be contacted directly via www.gisaid.org

Authors are sorted alphabetically.

Acknowledgement EPI_SET Identifier: EPI_SET_20220314np

Accession ID	Originating Laboratory	Submitting Laboratory	Authors
EPI_ISL_528538	Alsafar	Alsafar	Andreas Henschel; Ernesto Damiani; Gihan Elsir Ahmed Daw Elbait; Guan Tay; Habiba Alsafar; Rifat; Samuel Feng
EPI_ISL_416432	Clinical Microbiology Lab	Infectious Disease Research Department, King Abdullah International Medical Research Center (KAIMRC)	Abdulrahman Alswaji; Liliane Okdah; Majed Alghoribi; Michel Doumith; Sadeem Alhayli; Sameera Al Johani
EPI_ISL_416542, EPI_ISL_416543, EPI_ISL_421652	Dasman Diabetes Institute	Dasman Diabetes Institute	Ebaa Al-Ozairi; Ebaa AlOzairi; Fahd Al-Mulla; Motasem Melhem; Qais Al-Duwairi; Rasheeba Iqbal; Sara Al-Qabandi; Sumi John
EPI_ISL_416541	Dasman Diabetes Institute and Virology Laboratory Ministry of Health	Dasman Diabetes Institute	Ebaa AlOzairi; Fahd Al-Mulla; Motasem Melhem; Qais Al-Duwairi; Rasheeba Iqbal; Sara Al-Qabandi; Sumi John
EPI_ISL_422426, EPI_ISL_422427	JABER AL AHMAD AL SABAH HOSPITAL – KUWAIT CITY	Dasman Diabetes Institute	Ebaa Al-Ozairi; Fahd Al-Mulla; Qais Al-Duwairi; Rasheeba Iqbal; Sumi John
EPI_ISL_422424	Jaber Al Ahmad Al Sabah Hospital	Dasman diabetes Institute	Ebaa Al-Ozairi; Fahd Al-Mulla; Qais Al-Duwairi; Rasheeba Iqbal; Sumi John
EPI_ISL_489996, EPI_ISL_489997, EPI_ISL_489998, EPI_ISL_489999, EPI_ISL_490000, EPI_ISL_490001, EPI_ISL_490002, EPI_ISL_490003, EPI_ISL_490004, EPI_ISL_490005, EPI_ISL_490007, EPI_ISL_490008, EPI_ISL_490009, EPI_ISL_490010, EPI_ISL_490011, EPI_ISL_490012			
see above	King Fahad Medical City	King Fahad Medical City	Alghoraibi, M.; Alosaimi, B.; Enani, M.; Naeem, A.
EPI_ISL_483542, EPI_ISL_483543, EPI_ISL_483544, EPI_ISL_483546, EPI_ISL_483547, EPI_ISL_483548, EPI_ISL_483549, EPI_ISL_483550, EPI_ISL_483551, EPI_ISL_483552, EPI_ISL_483553, EPI_ISL_483554, EPI_ISL_483555, EPI_ISL_483556, EPI_ISL_483557, EPI_ISL_483558, EPI_ISL_483562, EPI_ISL_483563, EPI_ISL_483564, EPI_ISL_483565, EPI_ISL_483638, EPI_ISL_483639, EPI_ISL_483640			
see above	Kingdom of Bahrein Ministry of Health	Erasmus Medical Center	Amjad Ghanem Mohamed; Anne van der Linden; Bas Oude Munnink; Claudia Schapendonk; David Nieuwenhuijse; Ebrahim Shehad; Fatema; Hashmeya Al Wasti; Irina Chestakova; Marion Koopmans; Mark Pronk; Pascal Lexmond; Reina Sikkema; Richard Molenkamp; Stefan van Nieuwkoop; Theo Bestebroer; on behalf of the Dutch national COVID-19 response team.
EPI_ISL_427408, EPI_ISL_427416, EPI_ISL_427417, EPI_ISL_427418	Ministry of Public Health (MoPH)	Biomedical Research Center (BRC)	Abdullatif Al-Khal; Ajaeb D. M. H. Al-Nabet; Asmaa A. Al-Thani.; Einas A. E. Al-Kuwari; Fatiha M. Benslimane; Hadi M. Yassine; Hamad E. Al-Romaihi; Heba A. Al-Khatib; Mohammed Al-Thani; Muna A. S. Al-Maslamani; Nourah B. M. Younes; Peter V. Coyle; Salih Al-Marri; Sonia Boughattas
EPI_ISL_1712664, EPI_ISL_1712671, EPI_ISL_1712672, EPI_ISL_1712673, EPI_ISL_1712674, EPI_ISL_1712676, EPI_ISL_1712677, EPI_ISL_1712678, EPI_ISL_1712679, EPI_ISL_1712680, EPI_ISL_1712681, EPI_ISL_1712682, EPI_ISL_1712683, EPI_ISL_1712684, EPI_ISL_1712685, EPI_ISL_1712686, EPI_ISL_1712687, EPI_ISL_1712688, EPI_ISL_1712689, EPI_ISL_1712690, EPI_ISL_1712692, EPI_ISL_1712693, EPI_ISL_1712694, EPI_ISL_1712695, EPI_ISL_1712696, EPI_ISL_1712697, EPI_ISL_1712698, EPI_ISL_1712699, EPI_ISL_1712700, EPI_ISL_1712701, EPI_ISL_1712702, EPI_ISL_1712703, EPI_ISL_1712704, EPI_ISL_1712705, EPI_ISL_1712706, EPI_ISL_1712707, EPI_ISL_1713939			
see above	Ministry of Public Health / Hamad Medical Corporation	Biomedical Research Center (BRC), Qatar University / Qatar Genome Project (QGP)	Asmaa A. Al-Thani. MOPH and HMC: Abdullatif Al-Khal; BRC: Fatiha M. Benslimane; Chadi Saad; Dana Al-Batesh; Dina Elgakhlab QGP: Fatima H. Al-Kuwari; Einas A. E. Al-Kuwari; Hadi M. Yassine; Hamad E. Al-Romaihi; Hamda Alromaihi; Heba A. Al-Khatib; Mashael A. Al-Bader; Mohammed Al-Thani; Muna A. S. Al-Maslamani; Oal Al-Jamal; Peter V. Coyle; Reham A. El-Kahlout. QBB: Tasneem Al-Hamad; Roberto Bertollini; Salih Al-Marri
EPI_ISL_1713950, EPI_ISL_1713954, EPI_ISL_1713955, EPI_ISL_1713969, EPI_ISL_1713971, EPI_ISL_1713973, EPI_ISL_1713982, EPI_ISL_1713985, EPI_ISL_1713988, EPI_ISL_1713989, EPI_ISL_1713994, EPI_ISL_1713995, EPI_ISL_1714000, EPI_ISL_1714006, EPI_ISL_1714014, EPI_ISL_1714015, EPI_ISL_1714025, EPI_ISL_1714026, EPI_ISL_1714029, EPI_ISL_1714030, EPI_ISL_1714032, EPI_ISL_1714047, EPI_ISL_1714048, EPI_ISL_1714678, EPI_ISL_1714683, EPI_ISL_1714686, EPI_ISL_1714705, EPI_ISL_1714739, EPI_ISL_1714746			
see above	Ministry of Public Health / Hamad Medical Corporation	Weill Cornell Medical College - Qatar (WCM-Q), Genomics Core Laboratory / Qatar Genome Project (QGP)	Chadi Saad MOPH and HMC: Abdullatif Al-Khal; Dina Elgakhlab; Einas A. E. Al-Kuwari; Hamad E. Al-Romaihi; Hamda Alromaihi; Joel A Malek. QGP: Fatima H. Al-Kuwari; Laith Abu-Raddad; Mashael A. Al-Bader; Meryem Bensaad; Mohammed Al-Thani; Muna A. S. Al-Maslamani; Peter V. Coyle; Reham A. El-Kahlout. QBB: Tasneem Al-Hamad; Roberto Bertollini; Salih Al-Marri; Shameem Younuskunju; WCMQ: Ayeda A. Ahmed; Yasmin Mohamoud
EPI_ISL_435119	Mohammed Bin Rashid University of Medicine and Health Sciences	Al Jalila Children's Hospital	Abdulmajeed Alkhaja; Abiola Catherine Senok; Ahmad Abou Tayoun; Alawi Alsheikh-Ali; Divinlal Harilal; Hamda Khansaheb; Hanan Al Suwaidi; Mohammed Uddin; Norbert Nowotny; Qutayba Hamid; Rabih Halwani; Rifat Hamoudi; Rupa Murthy Varghese; Sathishkumar Ramaswamy; Tom Loney; Zulfa Omar Deesi
EPI_ISL_435120, EPI_ISL_435121, EPI_ISL_435122, EPI_ISL_435123, EPI_ISL_435124, EPI_ISL_435125, EPI_ISL_435126, EPI_ISL_435127, EPI_ISL_435128, EPI_ISL_435130, EPI_ISL_435131, EPI_ISL_435132, EPI_ISL_435133, EPI_ISL_435134, EPI_ISL_435135, EPI_ISL_435136, EPI_ISL_435137, EPI_ISL_435138, EPI_ISL_435140, EPI_ISL_435141, EPI_ISL_435142, EPI_ISL_435143, EPI_ISL_463740, EPI_ISL_469276, EPI_ISL_469278, EPI_ISL_469279, EPI_ISL_469280, EPI_ISL_469281, EPI_ISL_520664, EPI_ISL_520665, EPI_ISL_520666, EPI_ISL_520667, EPI_ISL_520668, EPI_ISL_520670, EPI_ISL_520680, EPI_ISL_520717, EPI_ISL_520718, EPI_ISL_520719, EPI_ISL_520720, EPI_ISL_520721, EPI_ISL_520722, EPI_ISL_520723, EPI_ISL_520724, EPI_ISL_520725, EPI_ISL_520726, EPI_ISL_520727, EPI_ISL_520728, EPI_ISL_520730, EPI_ISL_520731, EPI_ISL_520732, EPI_ISL_520734, EPI_ISL_520735, EPI_ISL_520736, EPI_ISL_520743, EPI_ISL_523953			
see above	Mohammed Bin Rashid University of Medicine and Health Sciences	Al Jalila Genomics Center	Abdulmajeed Alkhaja; Abiola Catherine Senok; Ahmad Abou Tayoun; Alawi Alsheikh-Ali; Divinlal Harilal; Hamda Khansaheb; Hanan Al Suwaidi; Mohammed Uddin; Norbert Nowotny; Qutayba Hamid; Rabih Halwani; Rifat Hamoudi; Rupa Murthy Varghese; Sathishkumar Ramaswamy; Tom Loney; Zulfa Omar Deesi
EPI_ISL_458119, EPI_ISL_458120, EPI_ISL_458121, EPI_ISL_458123	Oman National Influenza Centre	Department of Microbiology and Immunology-SQUH	Abdulla Balkhair; Ahlam Al-Amri; Aisha Al-Amri; Aisha Al-Busaidi; Amina Al Jardani; Fahad Zadjali; Fatma BaAlawi; Hamida AL Barwani; Hanan Al-kindi; Intisar Al-Shukri; Khulood Al-Mammary; Mohammed Al-Tobi; Samiha Al Kharusi; Samira Al-Maruqi; Zeyana AL-Dahmani
EPI_ISL_457707	Oman-NIC	Department of Microbiology and Immunology- SQUH	Abdulla Balkhair; Ahlam Al-Amri; Aisha Al-Amri; Aisha Al-Busaidi; Amina Al Jardani; Fahad Zadjali; Fatma BaAlawi; Hamida AL Barwani; Hanan Al-kindi; Intisar Al-Shukri; Khulood Al-Mammary; Mohammed Al-Tobi; Samiha Al Kharusi; Samira Al-Maruqi; Zeyana AL-Dahmani
EPI_ISL_457981	Oman-NIC	Department of Microbiology and Immunology-SQUH	Abdulla Balkhair; Ahlam Al-Amri; Aisha Al-Amri; Aisha Al-Busaidi; Amina Al Jardani; Fahad Zadjali; Fatma BaAlawi; Hamida AL Barwani; Hanan Al-kindi; Intisar Al-Shukri; Khulood Al-Mammary; Mohammed Al-Tobi; Samiha Al Kharusi; Samira Al-Maruqi; Zeyana AL-Dahmani
EPI_ISL_457702	Oman-NIC	Microbiology laboratory- Sultan Qaboos University Hospital	Abdulla Balkhair; Ahlam Al-Amri; Aisha Al-Amri; Aisha Al-Busaidi; Amina Al Jardani; Fahad Zadjali; Fatma BaAlawi; Hamida AL Barwani; Hanan Al-kindi; Intisar Al-Shukri; Khulood Al-Mammary; Mohammed Al-Tobi; Samiha Al Kharusi; Samira Al-Maruqi; Zeyana AL-Dahmani
EPI_ISL_457704, EPI_ISL_457706, EPI_ISL_457937, EPI_ISL_457938, EPI_ISL_457939, EPI_ISL_457974, EPI_ISL_457975, EPI_ISL_457976, EPI_ISL_457977, EPI_ISL_457978, EPI_ISL_457979, EPI_ISL_457980, EPI_ISL_457985, EPI_ISL_457986, EPI_ISL_457987, EPI_ISL_457988, EPI_ISL_457989, EPI_ISL_457990, EPI_ISL_457991, EPI_ISL_457992, EPI_ISL_457993, EPI_ISL_457994, EPI_ISL_457995, EPI_ISL_457996, EPI_ISL_457997, EPI_ISL_457998			
see above	Oman-NIC	Oman-NIC	Abdulla Balkhair; Ahlam Al-Amri; Aisha Al-Amri; Aisha Al-Busaidi; Amina Al Jardani; Fahad Zadjali; Fatma BaAlawi; Hamida AL Barwani; Hanan Al-kindi; Intisar Al-Shukri; Khulood Al-Mammary; Mohammed Al-Tobi; Samiha Al Kharusi; Samira Al-Maruqi; Zeyana AL-Dahmani
EPI_ISL_491116, EPI_ISL_491121, EPI_ISL_491123, EPI_ISL_491129, EPI_ISL_491134, EPI_ISL_491137, EPI_ISL_491143, EPI_ISL_491144, EPI_ISL_491147, EPI_ISL_491148, EPI_ISL_491149, EPI_ISL_491150, EPI_ISL_491152, EPI_ISL_491154, EPI_ISL_491156, EPI_ISL_491157, EPI_ISL_491163, EPI_ISL_491167, EPI_ISL_491168, EPI_ISL_491171, EPI_ISL_525423, EPI_ISL_525424, EPI_ISL_525425			
see above	Oman-National Influenza Center	Biotechnology & OMICs Laboratory	Abdul Latif Khan; Adil Al-Wahaibi; Adil Khan; Ahlam Al-Amri; Ahmed Al-Harrasi; Ahmed Al-Rawahi; Aisha Al-Amri; Aisha Al-Busaidi; Amina Al-Jardani; Hanan Al-Kindi; Intisar Al-Shukri; Sajjad Asaf; Samiha Al-Kharusi; Samira Al-Mahruqi; Seif Al-Abri; Seif Al-Abri.
EPI_ISL_518821	Oman-National Influenza Center	Biotechnology & OMICs Laboratory, Natural & Medical Sciences Research Center, University of Nizwa	Abdul Latif Khan; Adil Al-Wahaibi; Adil Khan; Ahmed Al-Harrasi; Ahmed Al-Rawahi; Amina Al-Jardani; Hanan Al-Kindi; Intisar Al-Shukri; Sajjad Asaf; Samiha Al-Kharusi; Samira Al-Mahruqi; Seif Al-Abri
EPI_ISL_437459, EPI_ISL_437460, EPI_ISL_437461, EPI_ISL_437462, EPI_ISL_437463, EPI_ISL_437464, EPI_ISL_437465, EPI_ISL_437466, EPI_ISL_437467, EPI_ISL_437468, EPI_ISL_437469, EPI_ISL_437470, EPI_ISL_437471, EPI_ISL_437472, EPI_ISL_437473, EPI_ISL_437474, EPI_ISL_437475, EPI_ISL_437477, EPI_ISL_437479, EPI_ISL_437481, EPI_ISL_437482, EPI_ISL_437484, EPI_ISL_437486, EPI_ISL_437489, EPI_ISL_437491, EPI_ISL_437492, EPI_ISL_437495, EPI_ISL_437496, EPI_ISL_437691, EPI_ISL_437692, EPI_ISL_437693, EPI_ISL_437694, EPI_ISL_437695, EPI_ISL_437696, EPI_ISL_437697, EPI_ISL_437698, EPI_ISL_437699, EPI_ISL_437703, EPI_ISL_437704, EPI_ISL_437705, EPI_ISL_437706, EPI_ISL_437707, EPI_ISL_437709, EPI_ISL_437711, EPI_ISL_437713, EPI_ISL_437715, EPI_ISL_437716, EPI_ISL_437718, EPI_ISL_437720, EPI_ISL_437721, EPI_ISL_437722, EPI_ISL_437723, EPI_ISL_437724, EPI_ISL_437725, EPI_ISL_437726, EPI_ISL_437727, EPI_ISL_437729, EPI_ISL_437731, EPI_ISL_437733, EPI_ISL_437739, EPI_ISL_437740, EPI_ISL_437741, EPI_ISL_437743, EPI_ISL_437745, EPI_ISL_437746, EPI_ISL_437747, EPI_ISL_437748, EPI_ISL_437751, EPI_ISL_437752, EPI_ISL_437753, EPI_ISL_437754, EPI_ISL_437756, EPI_ISL_437757, EPI_ISL_437759, EPI_ISL_437760, EPI_ISL_437761, EPI_ISL_512908, EPI_ISL_512909, EPI_ISL_512911, EPI_ISL_512912, EPI_ISL_512913, EPI_ISL_512914, EPI_ISL_512915, EPI_ISL_512916, EPI_ISL_512917, EPI_ISL_512918, EPI_ISL_512928, EPI_ISL_512931, EPI_ISL_512932, EPI_ISL_512933, EPI_ISL_512934, EPI_ISL_512936, EPI_ISL_512937, EPI_ISL_512938, EPI_ISL_512939, EPI_ISL_512943, EPI_ISL_512956, EPI_ISL_512957, EPI_ISL_512959, EPI_ISL_512979, EPI_ISL_512981, EPI_ISL_512989, EPI_ISL_512993, EPI_ISL_512995, EPI_ISL_512996, EPI_ISL_512997, EPI_ISL_512998, EPI_ISL_513001, EPI_ISL_513041, EPI_ISL_513042, EPI_ISL_513043, EPI_ISL_513044, EPI_ISL_513045, EPI_ISL_513046, EPI_ISL_513047, EPI_ISL_513048, EPI_ISL_513050, EPI_ISL_513051, EPI_ISL_513052, EPI_ISL_513056, EPI_ISL_513057, EPI_ISL_513058, EPI_ISL_513059, EPI_ISL_513061, EPI_ISL_513062, EPI_ISL_513121, EPI_ISL_513125, EPI_ISL_513128, EPI_ISL_513158, EPI_ISL_513159, EPI_ISL_513160, EPI_ISL_513161, EPI_ISL_513162, EPI_ISL_513163, EPI_ISL_513164, EPI_ISL_513165, EPI_ISL_513167, EPI_ISL_513173, EPI_ISL_513182, EPI_ISL_513183, EPI_ISL_513188, EPI_ISL_513189, EPI_ISL_513214, EPI_ISL_513217, EPI_ISL_513218, EPI_ISL_513219, EPI_ISL_513221, EPI_ISL_513222, EPI_ISL_513224, EPI_ISL_513229, EPI_ISL_513231, EPI_ISL_513234, EPI_ISL_513235, EPI_ISL_513238, EPI_ISL_513240, EPI_ISL_513241, EPI_ISL_513243, EPI_ISL_513244, EPI_ISL_513245, EPI_ISL_513247, EPI_ISL_677940, EPI_ISL_677943, EPI_ISL_677968, EPI_ISL_677973, EPI_ISL_677976, EPI_ISL_678026, EPI_ISL_678029, EPI_ISL_678031, EPI_ISL_678036, EPI_ISL_678037, EPI_ISL_678065, EPI_ISL_678066, EPI_ISL_678068, EPI_ISL_678071, EPI_ISL_678072, EPI_ISL_678073, EPI_ISL_678074, EPI_ISL_678075, EPI_ISL_678076, EPI_ISL_678077, EPI_ISL_678079, EPI_ISL_678080, EPI_ISL_678081, EPI_ISL_678082, EPI_ISL_678097, EPI_ISL_678100, EPI_ISL_678101, EPI_ISL_678102, EPI_ISL_678103, EPI_ISL_678104, EPI_ISL_678105, EPI_ISL_678107, EPI_ISL_678109, EPI_ISL_678110, EPI_ISL_678111, EPI_ISL_678112, EPI_ISL_678113, EPI_ISL_678114, EPI_ISL_678115, EPI_ISL_678116, EPI_ISL_678117, EPI_ISL_678118, EPI_ISL_678119, EPI_ISL_678120, EPI_ISL_678121, EPI_ISL_678123, EPI_ISL_678136, EPI_ISL_678137, EPI_ISL_678139, EPI_ISL_678140, EPI_ISL_678142, EPI_ISL_678229, EPI_ISL_678230, EPI_ISL_678231, EPI_ISL_678233, EPI_ISL_751221, EPI_ISL_751223, EPI_ISL_751224, EPI_ISL_751237, EPI_ISL_751238			
see above	Pathogen Genomics Lab King Abdullah University of Science and Technology(KAUST)	Pathogen Genomics Lab King Abdullah University of Science and Technology(KAUST)	Abdulaziz Alahmadi; Afrah Alsomali; Ahmad Bakur Mahmoud; Amanda; Amanda Ooi; Amit Kumar Subudhi; Amit Subudhi; Anwar Hashem; Arnab Pain; Asim Khogeer; Fadwa Alofi; Fathia Ben Rached; Fathia Ben-Rached; Fathia Rached; Jumana Taha; Kahled Algithami; Khaled Algithami; Luke; Luke Esau; Naif Almontashiri; Nashwa Al-khotani; Olga Douvropoulou; Raeece Naeem; Rahul P Salunke; Rahul Salunke; Raushan Nugmanova; Sara Mfarrej; Sharif Hala
EPI_ISL_416521	Public Health Laboratory	Public Health Laboratory, Saudi CDC	A; Albarrag
EPI_ISL_582125, EPI_ISL_582126	Sheikh Khalifa Medical City	Molecular Surveillance lab Sheikh Khalifa Medical City	Amirtharaj Francis; Hala Imambaccus; Hiba Saud; Sahar Almarzooqi; Sajeed Abdul; Stefan Weber
EPI_ISL_582613, EPI_ISL_582620, EPI_ISL_582621, EPI_ISL_582624, EPI_ISL_582625, EPI_ISL_582632	Sheikh Khalifa Medical City	Molecular/Surveillance lab Sheikh Khalifa Medical City	Amirtharaj Francis; Hala Imambaccus; Hiba Saud; Sahar Almarzooqi; Sajeed Abdul; Stefan Weber
EPI_ISL_469241, EPI_ISL_469243, EPI_ISL_469244, EPI_ISL_469245, EPI_ISL_469246, EPI_ISL_469247, EPI_ISL_469248, EPI_ISL_469249, EPI_ISL_469250, EPI_ISL_469251			
see above	Special Infectious Agents Unit	Special Infectious Agents Unit	A.M.; Al-Sobahy; Azhar; E.I.; El-Kafrawy; Farraj; Hassan; N.A.; S.A.; T.L.; Tolah; Uthman
EPI_ISL_507010, EPI_ISL_507011, EPI_ISL_507012, EPI_ISL_507013, EPI_ISL_507014, EPI_ISL_507015, EPI_ISL_507016, EPI_ISL_507017, EPI_ISL_507018, EPI_ISL_507019, EPI_ISL_507020, EPI_ISL_507021, EPI_ISL_507022, EPI_ISL_507023, EPI_ISL_507024, EPI_ISL_507025, EPI_ISL_507026, EPI_ISL_507027, EPI_ISL_507028, EPI_ISL_507029, EPI_ISL_507030, EPI_ISL_507031, EPI_ISL_507032, EPI_ISL_507033, EPI_ISL_507034, EPI_ISL_507035, EPI_ISL_507036, EPI_ISL_507037, EPI_ISL_507038			
see above	unknown	Infectious Diseases Research, King Abdullah International Medical Research Center (KAIMRC)	Alghoribi; M.F.

We gratefully acknowledge the following Authors from the Originating laboratories responsible for obtaining the specimens, as well as the Submitting laboratories where the genome data were generated and shared via GISAID, on which this research is based.

All Submitters of data may be contacted directly via www.gisaid.org

Authors are sorted alphabetically.

Acknowledgement EPI_SET Identifier: EPI_SET_20220314xy

Accession ID	Originating Laboratory	Submitting Laboratory	Authors
EPI_ISL_528686, EPI_ISL_528687, EPI_ISL_528688, EPI_ISL_528689, EPI_ISL_528690, EPI_ISL_528692, EPI_ISL_528693, EPI_ISL_528698, EPI_ISL_528699, EPI_ISL_528701, EPI_ISL_528704, EPI_ISL_528709, EPI_ISL_528712, EPI_ISL_528714, EPI_ISL_528715, EPI_ISL_528716, EPI_ISL_528717, EPI_ISL_528718, EPI_ISL_528719, EPI_ISL_528720, EPI_ISL_528721			
see above	Alsafar - Khalifa University Abu Dhabi	Alsafar - Khalifa University Abu Dhabi	Andreas Henschel; Ernesto Damiani; Gihan Daw Elbait; Guan Tay; Habiba Alsafar; Rifat Hamoudi; Samuel Feng
EPI_ISL_859936, EPI_ISL_859937, EPI_ISL_859938, EPI_ISL_859939, EPI_ISL_859940, EPI_ISL_859942, EPI_ISL_859943, EPI_ISL_859945, EPI_ISL_859946, EPI_ISL_859951, EPI_ISL_859952, EPI_ISL_859954, EPI_ISL_859955, EPI_ISL_859956, EPI_ISL_859957, EPI_ISL_859958, EPI_ISL_859959, EPI_ISL_859960, EPI_ISL_859962, EPI_ISL_859964, EPI_ISL_859966, EPI_ISL_859967, EPI_ISL_859968, EPI_ISL_859969, EPI_ISL_859970, EPI_ISL_859971, EPI_ISL_859973, EPI_ISL_859974, EPI_ISL_859975, EPI_ISL_859976, EPI_ISL_859979, EPI_ISL_859980, EPI_ISL_859981, EPI_ISL_859983, EPI_ISL_859985, EPI_ISL_859987, EPI_ISL_859988, EPI_ISL_859989, EPI_ISL_859990, EPI_ISL_859991, EPI_ISL_859992, EPI_ISL_859994, EPI_ISL_859995, EPI_ISL_859997, EPI_ISL_859998, EPI_ISL_860000, EPI_ISL_860001, EPI_ISL_860002, EPI_ISL_860003, EPI_ISL_860006, EPI_ISL_860007, EPI_ISL_860008, EPI_ISL_860009, EPI_ISL_860010, EPI_ISL_860011, EPI_ISL_860012, EPI_ISL_860013, EPI_ISL_860014, EPI_ISL_860015, EPI_ISL_860016, EPI_ISL_860017, EPI_ISL_860020, EPI_ISL_860022, EPI_ISL_860023, EPI_ISL_860024			
see above	BTC, Khalifa University	BTC, Khalifa University	Al Safar et al
EPI_ISL_548966, EPI_ISL_548967, EPI_ISL_548968, EPI_ISL_548969, EPI_ISL_548970, EPI_ISL_548971	Expo2020 Emergency Center	Agiomix	Cengiz Yakicier; Cherif Ben Hamada; Rashid Mohammed; Tamer Degheidy; Walaa Allam; Walid Dridi
EPI_ISL_698155, EPI_ISL_698156, EPI_ISL_698157, EPI_ISL_698158, EPI_ISL_698159, EPI_ISL_698163, EPI_ISL_698164, EPI_ISL_698165, EPI_ISL_698166, EPI_ISL_698167, EPI_ISL_698168, EPI_ISL_698178, EPI_ISL_698181, EPI_ISL_698182, EPI_ISL_698189, EPI_ISL_698198, EPI_ISL_698199, EPI_ISL_698213, EPI_ISL_698215, EPI_ISL_698216, EPI_ISL_698219, EPI_ISL_698220, EPI_ISL_698222, EPI_ISL_698223, EPI_ISL_698224, EPI_ISL_698225, EPI_ISL_698226, EPI_ISL_698227, EPI_ISL_698228, EPI_ISL_698229, EPI_ISL_698230, EPI_ISL_698231, EPI_ISL_698232, EPI_ISL_698233, EPI_ISL_698265, EPI_ISL_698266, EPI_ISL_698267, EPI_ISL_698281, EPI_ISL_698282, EPI_ISL_698283, EPI_ISL_698284, EPI_ISL_698285, EPI_ISL_698295, EPI_ISL_698296, EPI_ISL_698297, EPI_ISL_698298, EPI_ISL_698299, EPI_ISL_698300, EPI_ISL_698301, EPI_ISL_698302, EPI_ISL_698303, EPI_ISL_698304, EPI_ISL_698306, EPI_ISL_698307, EPI_ISL_698308, EPI_ISL_698309, EPI_ISL_698310, EPI_ISL_698521, EPI_ISL_698522, EPI_ISL_698525, EPI_ISL_698528, EPI_ISL_698536, EPI_ISL_698538, EPI_ISL_698539, EPI_ISL_698540, EPI_ISL_698541, EPI_ISL_698543, EPI_ISL_698544, EPI_ISL_698545, EPI_ISL_698546, EPI_ISL_698547, EPI_ISL_698549, EPI_ISL_698551, EPI_ISL_698556, EPI_ISL_698557, EPI_ISL_698559, EPI_ISL_698561, EPI_ISL_698563, EPI_ISL_698567, EPI_ISL_698568, EPI_ISL_698579, EPI_ISL_698580, EPI_ISL_698581, EPI_ISL_698583, EPI_ISL_698586, EPI_ISL_698587, EPI_ISL_698588, EPI_ISL_698589, EPI_ISL_698591, EPI_ISL_698592, EPI_ISL_698593, EPI_ISL_698594, EPI_ISL_698607, EPI_ISL_698608, EPI_ISL_698609, EPI_ISL_698610, EPI_ISL_698611, EPI_ISL_698708, EPI_ISL_698709, EPI_ISL_698710, EPI_ISL_698711, EPI_ISL_698712, EPI_ISL_698713, EPI_ISL_698714, EPI_ISL_698723, EPI_ISL_698725, EPI_ISL_698726, EPI_ISL_698727, EPI_ISL_698728, EPI_ISL_698729, EPI_ISL_698730, EPI_ISL_698751, EPI_ISL_698752, EPI_ISL_698754, EPI_ISL_698755, EPI_ISL_698756, EPI_ISL_698757, EPI_ISL_698758, EPI_ISL_698759, EPI_ISL_698761, EPI_ISL_698762, EPI_ISL_698764			
see above	Group 42 (G42) Healthcare, Abu Dhabi, United Arab Emirates; Department of Health, The United Arab Emirates	G42 Healthcare	Ashish Koshy; Budoor Alqarni; Denghui Liu; Fang Chen; Hanif Khalak; Huanming Yang; Javier Quilez; Jian Wang; Junhua Li; Ke Liang; Long Lin; Mohammed Saifuddin Fasihuddin; Nan Qiao; Nawal Ahmed Mohamed Al Kaabi; Pauline Ogrodzki; Pei Wu; Peng Xiao; Pengjuan Liu; Rong Liu; Sally Mahmoud; Siyang Liu; Stephen S. Francis; Tao Ma; Vinay Kusuma; Walid Abbas Zaher; Weibin Liu; Wenjun He; Xavier Anton; Xin Jin; Xin Meng; Xinyu Huang; Xun Xu; Zhaorong Yuan
EPI_ISL_1712729, EPI_ISL_1712748, EPI_ISL_1712749, EPI_ISL_1712751, EPI_ISL_1712752, EPI_ISL_1712753, EPI_ISL_1712754, EPI_ISL_1712755, EPI_ISL_1712758, EPI_ISL_1712761, EPI_ISL_1712764, EPI_ISL_1712766, EPI_ISL_1712773, EPI_ISL_1712774, EPI_ISL_1712776, EPI_ISL_1712777, EPI_ISL_1712780, EPI_ISL_1712785, EPI_ISL_1712787, EPI_ISL_1712791, EPI_ISL_1712792			
see above	Ministry of Public Health / Hamad Medical Corporation	Biomedical Research Center (BRC), Qatar University / Qatar Genome Project (QGP)	Asmaa A. Al-Thani. MOPH and HMC: Abdullatif Al-Khal; BRC: Fatiha M. Benslimane; Chadi Saad; Dana Al-Batesh; Dina Elgakhlab QGP: Fatima H. Al-Kuwari; Einas A. E. Al-Kuwari; Hadi M. Yassine; Hamad E. Al-Romaihi; Hamda Alromaihi; Heba A. Al-Khatib; Mashael A. Al-Bader; Mohammed Al-Thani; Muna A. S. Al-Maslamani; Oal Al-Jamal; Peter V. Coyle; Reham A. El-Kahlout. QBB: Tasneem Al-Hamad; Roberto Bertollini; Salih Al-Marri
EPI_ISL_1713947, EPI_ISL_1713948, EPI_ISL_1713949, EPI_ISL_1713953, EPI_ISL_1713956, EPI_ISL_1713957, EPI_ISL_1713961, EPI_ISL_1713962, EPI_ISL_1713965, EPI_ISL_1713966, EPI_ISL_1713968, EPI_ISL_1713970, EPI_ISL_1713972, EPI_ISL_1713974, EPI_ISL_1713975, EPI_ISL_1713976, EPI_ISL_1713977, EPI_ISL_1713978, EPI_ISL_1713979, EPI_ISL_1713980, EPI_ISL_1713981, EPI_ISL_1713983, EPI_ISL_1713984, EPI_ISL_1713986, EPI_ISL_1713987, EPI_ISL_1713990, EPI_ISL_1713991, EPI_ISL_1713992, EPI_ISL_1713993, EPI_ISL_1713996, EPI_ISL_1713997, EPI_ISL_1713998, EPI_ISL_1713999, EPI_ISL_1714001, EPI_ISL_1714002, EPI_ISL_1714003, EPI_ISL_1714004, EPI_ISL_1714005, EPI_ISL_1714007, EPI_ISL_1714008, EPI_ISL_1714009, EPI_ISL_1714010, EPI_ISL_1714011, EPI_ISL_1714012, EPI_ISL_1714013, EPI_ISL_1714016, EPI_ISL_1714017, EPI_ISL_1714018, EPI_ISL_1714019, EPI_ISL_1714020, EPI_ISL_1714021, EPI_ISL_1714022, EPI_ISL_1714027, EPI_ISL_1714028, EPI_ISL_1714031, EPI_ISL_1714033, EPI_ISL_1714034, EPI_ISL_1714035, EPI_ISL_1714036, EPI_ISL_1714037, EPI_ISL_1714038, EPI_ISL_1714039, EPI_ISL_1714041, EPI_ISL_1714042, EPI_ISL_1714043, EPI_ISL_1714044, EPI_ISL_1714058, EPI_ISL_1714060, EPI_ISL_1714064, EPI_ISL_1714065, EPI_ISL_1714066, EPI_ISL_1714067, EPI_ISL_1714074, EPI_ISL_1714091, EPI_ISL_1714093, EPI_ISL_1714096, EPI_ISL_1714102, EPI_ISL_1714110, EPI_ISL_1714112, EPI_ISL_1714114, EPI_ISL_1714186, EPI_ISL_1714358, EPI_ISL_1714359, EPI_ISL_1714363, EPI_ISL_1714364, EPI_ISL_1714563, EPI_ISL_1714572, EPI_ISL_1714573, EPI_ISL_1714576, EPI_ISL_1714577, EPI_ISL_1714594, EPI_ISL_1714597, EPI_ISL_1714599, EPI_ISL_1714600, EPI_ISL_1714610, EPI_ISL_1714611, EPI_ISL_1714634, EPI_ISL_1714635, EPI_ISL_1714669, EPI_ISL_1714671, EPI_ISL_1714672, EPI_ISL_1714675, EPI_ISL_1714681, EPI_ISL_1714684, EPI_ISL_1714687, EPI_ISL_1714692, EPI_ISL_1714693, EPI_ISL_1714694, EPI_ISL_1714696, EPI_ISL_1714697, EPI_ISL_1714699, EPI_ISL_1714700, EPI_ISL_1714702, EPI_ISL_1714703, EPI_ISL_1714704, EPI_ISL_1714707, EPI_ISL_1714708, EPI_ISL_1714710, EPI_ISL_1714711, EPI_ISL_1714712, EPI_ISL_1714713, EPI_ISL_1714714, EPI_ISL_1714715, EPI_ISL_1714717, EPI_ISL_1714718, EPI_ISL_1714719, EPI_ISL_1714720, EPI_ISL_1714727, EPI_ISL_1714733, EPI_ISL_1714735, EPI_ISL_1714740, EPI_ISL_1714747, EPI_ISL_1714748, EPI_ISL_1714749			
see above	Ministry of Public Health / Hamad Medical Corporation	Weill Cornell Medical College - Qatar (WCM-Q), Genomics Core Laboratory / Qatar Genome Project (QGP)	Chadi Saad MOPH and HMC: Abdullatif Al-Khal; Dina Elgakhlab; Einas A. E. Al-Kuwari; Hamad E. Al-Romaihi; Hamda Alromaihi; Joel A Malek. QGP: Fatima H. Al-Kuwari; Laith Abu-Raddad; Mashael A. Al-Bader; Meryem Bensaad; Mohammed Al-Thani; Muna A. S. Al-Maslamani; Peter V. Coyle; Reham A. El-Kahlout. QBB: Tasneem Al-Hamad; Roberto Bertollini; Salih Al-Marri; Shameem Younuskunju; WCMQ: Ayeda A. Ahmed; Yasmin Mohamoud
EPI_ISL_469277, EPI_ISL_520663, EPI_ISL_520672, EPI_ISL_520673, EPI_ISL_520674, EPI_ISL_520675, EPI_ISL_520681, EPI_ISL_520682, EPI_ISL_520683, EPI_ISL_520684, EPI_ISL_520685, EPI_ISL_520686, EPI_ISL_520716, EPI_ISL_520738, EPI_ISL_520740, EPI_ISL_520741, EPI_ISL_520742			
see above	Mohammed Bin Rashid University of Medicine and Health Sciences	Al Jalila Genomics Center	Abdulmajeed Alkhaja; Abiola Catherine Senok; Ahmad Abou Tayoun; Alawi Alsheikh-Ali; Divinlal Harilal; Hamda Khansaheb; Hanan Al Suwaidi; Mohammed Uddin; Norbert Nowotny; Qutayba Hamid; Rabih Halwani; Rifat Hamoudi; Rupa Murthy Varghese; Sathishkumar Ramaswamy; Tom Loney; Zulfa Omar Deesi
EPI_ISL_457705	OMAN-NIC	Department of Microbiology and Immunology- SQUH	Abdulla Balkhair; Ahlam Al-Amri; Aisha Al-Amri; Aisha Al-Busaidi; Amina Al Jardani; Fahad Zadjali; Fatma BaAlawi; Hamida AL Barwani; Hanan Al-kindi; Intisar Al-Shukri; Khulood Al-Mammary; Mohammed Al-Tobi; Samiha Al Kharusi; Samira Al-Maruqi; Zeyana AL-Dahmani
EPI_ISL_458116, EPI_ISL_458117, EPI_ISL_458118, EPI_ISL_458124	Oman National Influenza Centre	Department of Microbiology and Immunology-SQUH	Abdulla Balkhair; Ahlam Al-Amri; Aisha Al-Amri; Aisha Al-Busaidi; Amina Al Jardani; Fahad Zadjali; Fatma BaAlawi; Hamida AL Barwani; Hanan Al-kindi; Intisar Al-Shukri; Khulood Al-Mammary; Mohammed Al-Tobi; Samiha Al Kharusi; Samira Al-Maruqi; Zeyana AL-Dahmani
EPI_ISL_491970, EPI_ISL_491973, EPI_ISL_491974	Oman-NIC	Department of Microbiology and Immunology-SQUH	Abdulla Balkhair; Ahlam Al-Amri; Aisha Al-Amri; Aisha Al-Busaidi; Amina Al Jardani; Fahad Zadjali; Fatma BaAlawi; Hamida AL Barwani; Hanan Al-kindi; Intisar Al-Shukri; Khulood Al-Mammary; Mohammed Al-Tobi; Samiha Al Kharusi; Samira Al-Maruqi; Zeyana AL-Dahmani
EPI_ISL_491122, EPI_ISL_491124, EPI_ISL_491125, EPI_ISL_491126, EPI_ISL_491127, EPI_ISL_491132, EPI_ISL_491136, EPI_ISL_491141, EPI_ISL_491142, EPI_ISL_491145, EPI_ISL_491146, EPI_ISL_491153, EPI_ISL_491165, EPI_ISL_491169			
see above	Oman-National Influenza Center	Biotechnology & OMICs Laboratory	Abdul Latif Khan; Adil Al-Wahaibi; Adil Khan; Ahlam Al-Amri; Ahmed Al-Harrasi; Ahmed Al-Rawahi; Aisha Al-Amri; Aisha Al-Busaidi; Amina Al-Jardani; Hanan Al-Kindi; Intisar Al-Shukri; Sajjad Asaf; Samiha Al-Kharusi; Samira Al-Mahruqi; Seif Al-Abri; Seif Al-Abri.
EPI_ISL_518843, EPI_ISL_518844, EPI_ISL_518845, EPI_ISL_518846, EPI_ISL_518848, EPI_ISL_518849, EPI_ISL_518850, EPI_ISL_518851, EPI_ISL_518852, EPI_ISL_518853, EPI_ISL_518854			
see above	Oman-National Influenza Center	Biotechnology & OMICs Laboratory, Natural & Medical Sciences Research Center, University of Nizwa	Abdul Latif Khan; Adil Al-Wahaibi; Adil Khan; Ahlam Al-Amri; Ahmed Al-Harrasi; Ahmed Al-Rawahi; Aisha Al-Amri; Aisha Al-Busaidi; Amina Al-Jardani; Hanan Al-Kindi; Intisar Al-Shukri; Sajjad Asaf; Samiha Al-Kharusi; Samira Al-Mahruqi; Seif Al-Abri; Seif Al-Abri.
EPI_ISL_512944, EPI_ISL_512945, EPI_ISL_512947, EPI_ISL_512948, EPI_ISL_512950, EPI_ISL_512952, EPI_ISL_512955, EPI_ISL_512961, EPI_ISL_512964, EPI_ISL_512966, EPI_ISL_512967, EPI_ISL_512968, EPI_ISL_512969, EPI_ISL_512970, EPI_ISL_512971, EPI_ISL_512973, EPI_ISL_512983, EPI_ISL_512984, EPI_ISL_512985, EPI_ISL_512986, EPI_ISL_512987, EPI_ISL_512990, EPI_ISL_513127, EPI_ISL_513129, EPI_ISL_513131, EPI_ISL_513132, EPI_ISL_513134, EPI_ISL_513137, EPI_ISL_513138, EPI_ISL_513139, EPI_ISL_513141, EPI_ISL_513143, EPI_ISL_513148, EPI_ISL_513150, EPI_ISL_513152, EPI_ISL_513153, EPI_ISL_513154, EPI_ISL_513168, EPI_ISL_513169, EPI_ISL_513172, EPI_ISL_513174, EPI_ISL_513175, EPI_ISL_513176, EPI_ISL_513178, EPI_ISL_513179, EPI_ISL_513180, EPI_ISL_513181, EPI_ISL_513206, EPI_ISL_513208, EPI_ISL_513239, EPI_ISL_513248, EPI_ISL_513249, EPI_ISL_513250, EPI_ISL_513251, EPI_ISL_513254, EPI_ISL_513257, EPI_ISL_513260, EPI_ISL_513261, EPI_ISL_513263, EPI_ISL_513264, EPI_ISL_636964, EPI_ISL_677938, EPI_ISL_677939, EPI_ISL_677942, EPI_ISL_678032, EPI_ISL_678033, EPI_ISL_678034, EPI_ISL_678067, EPI_ISL_678124, EPI_ISL_678125, EPI_ISL_678126, EPI_ISL_678128, EPI_ISL_678130, EPI_ISL_678131, EPI_ISL_678132, EPI_ISL_678133, EPI_ISL_678134, EPI_ISL_678145, EPI_ISL_678146, EPI_ISL_678246			
see above	Pathogen Genomics Lab King Abdullah University of Science and Technology(KAUST)	Pathogen Genomics Lab King Abdullah University of Science and Technology(KAUST)	Abdulaziz Alahmadi; Afrah Alsomali; Ahmad Bakur Mahmoud; Amanda; Amanda Ooi; Amit Kumar Subudhi; Anwar Hashem; Arnab Pain; Asim Khogeer; Fadwa Alofi; Fathia Ben Rached; Jumana Taha; Kahled Algithami; Luke; Luke Esau; Naif Almontashiri; Nashwa Al-khotani; Olga Douvropoulou; Raeece Naeem; Rahul P Salunke; Raushan Nugmanova; Sara Mfarrej; Sharif Hala

We gratefully acknowledge the following Authors from the Originating laboratories responsible for obtaining the specimens, as well as the Submitting laboratories where the genome data were generated and shared via GISAID, on which this research is based.

All Submitters of data may be contacted directly via www.gisaid.org

Authors are sorted alphabetically.

Acknowledgement EPI_SET Identifier: EPI_SET_20220314kt

Accession ID	Originating Laboratory	Submitting Laboratory	Authors
EPI_ISL_698160, EPI_ISL_698161, EPI_ISL_698169, EPI_ISL_698172, EPI_ISL_698173, EPI_ISL_698174, EPI_ISL_698175, EPI_ISL_698176, EPI_ISL_698177, EPI_ISL_698183, EPI_ISL_698184, EPI_ISL_698185, EPI_ISL_698190, EPI_ISL_698192, EPI_ISL_698193, EPI_ISL_698195, EPI_ISL_698196, EPI_ISL_698197, EPI_ISL_698200, EPI_ISL_698202, EPI_ISL_698203, EPI_ISL_698204, EPI_ISL_698207, EPI_ISL_698208, EPI_ISL_698209, EPI_ISL_698210, EPI_ISL_698234, EPI_ISL_698235, EPI_ISL_698236, EPI_ISL_698237, EPI_ISL_698238, EPI_ISL_698239, EPI_ISL_698240, EPI_ISL_698242, EPI_ISL_698243, EPI_ISL_698244, EPI_ISL_698245, EPI_ISL_698246, EPI_ISL_698248, EPI_ISL_698250, EPI_ISL_698251, EPI_ISL_698252, EPI_ISL_698255, EPI_ISL_698256, EPI_ISL_698257, EPI_ISL_698258, EPI_ISL_698259, EPI_ISL_698260, EPI_ISL_698261, EPI_ISL_698262, EPI_ISL_698263, EPI_ISL_698264, EPI_ISL_698268, EPI_ISL_698269, EPI_ISL_698270, EPI_ISL_698271, EPI_ISL_698273, EPI_ISL_698274, EPI_ISL_698275, EPI_ISL_698276, EPI_ISL_698278, EPI_ISL_698279, EPI_ISL_698280, EPI_ISL_698286, EPI_ISL_698287, EPI_ISL_698288, EPI_ISL_698289, EPI_ISL_698290, EPI_ISL_698291, EPI_ISL_698292, EPI_ISL_698293, EPI_ISL_698294, EPI_ISL_698311, EPI_ISL_698312, EPI_ISL_698313, EPI_ISL_698315, EPI_ISL_698316, EPI_ISL_698317, EPI_ISL_698318, EPI_ISL_698320, EPI_ISL_698321, EPI_ISL_698322, EPI_ISL_698323, EPI_ISL_698326, EPI_ISL_698327, EPI_ISL_698330, EPI_ISL_698331, EPI_ISL_698333, EPI_ISL_698334, EPI_ISL_698335, EPI_ISL_698336, EPI_ISL_698337, EPI_ISL_698338, EPI_ISL_698339, EPI_ISL_698340, EPI_ISL_698341, EPI_ISL_698342, EPI_ISL_698343, EPI_ISL_698344, EPI_ISL_698345, EPI_ISL_698348, EPI_ISL_698351, EPI_ISL_698352, EPI_ISL_698353, EPI_ISL_698354, EPI_ISL_698355, EPI_ISL_698356, EPI_ISL_698357, EPI_ISL_698358, EPI_ISL_698359, EPI_ISL_698360, EPI_ISL_698362, EPI_ISL_698363, EPI_ISL_698364, EPI_ISL_698365, EPI_ISL_698366, EPI_ISL_698367, EPI_ISL_698368, EPI_ISL_698369, EPI_ISL_698370, EPI_ISL_698371, EPI_ISL_698372, EPI_ISL_698373, EPI_ISL_698374, EPI_ISL_698375, EPI_ISL_698376, EPI_ISL_698377, EPI_ISL_698378, EPI_ISL_698379, EPI_ISL_698380, EPI_ISL_698382, EPI_ISL_698383, EPI_ISL_698385, EPI_ISL_698386, EPI_ISL_698387, EPI_ISL_698388, EPI_ISL_698389, EPI_ISL_698390, EPI_ISL_698392, EPI_ISL_698393, EPI_ISL_698394, EPI_ISL_698395, EPI_ISL_698397, EPI_ISL_698398, EPI_ISL_698400, EPI_ISL_698401, EPI_ISL_698402, EPI_ISL_698403, EPI_ISL_698404, EPI_ISL_698405, EPI_ISL_698406, EPI_ISL_698407, EPI_ISL_698408, EPI_ISL_698409, EPI_ISL_698411, EPI_ISL_698413, EPI_ISL_698414, EPI_ISL_698416, EPI_ISL_698417, EPI_ISL_698419, EPI_ISL_698423, EPI_ISL_698424, EPI_ISL_698425, EPI_ISL_698426, EPI_ISL_698427, EPI_ISL_698428, EPI_ISL_698429, EPI_ISL_698430, EPI_ISL_698431, EPI_ISL_698432, EPI_ISL_698433, EPI_ISL_698434, EPI_ISL_698435, EPI_ISL_698436, EPI_ISL_698437, EPI_ISL_698438, EPI_ISL_698439, EPI_ISL_698440, EPI_ISL_698441, EPI_ISL_698442, EPI_ISL_698443, EPI_ISL_698444, EPI_ISL_698445, EPI_ISL_698446, EPI_ISL_698447, EPI_ISL_698448, EPI_ISL_698449, EPI_ISL_698450, EPI_ISL_698451, EPI_ISL_698453, EPI_ISL_698454, EPI_ISL_698455, EPI_ISL_698457, EPI_ISL_698458, EPI_ISL_698459, EPI_ISL_698461, EPI_ISL_698462, EPI_ISL_698465, EPI_ISL_698468, EPI_ISL_698470, EPI_ISL_698471, EPI_ISL_698472, EPI_ISL_698473, EPI_ISL_698474, EPI_ISL_698475, EPI_ISL_698476, EPI_ISL_698478, EPI_ISL_698480, EPI_ISL_698481, EPI_ISL_698482, EPI_ISL_698483, EPI_ISL_698486, EPI_ISL_698488, EPI_ISL_698489, EPI_ISL_698490, EPI_ISL_698491, EPI_ISL_698492, EPI_ISL_698494, EPI_ISL_698495, EPI_ISL_698496, EPI_ISL_698498, EPI_ISL_698499, EPI_ISL_698500, EPI_ISL_698501, EPI_ISL_698502, EPI_ISL_698503, EPI_ISL_698504, EPI_ISL_698505, EPI_ISL_698506, EPI_ISL_698508, EPI_ISL_698509, EPI_ISL_698510, EPI_ISL_698511, EPI_ISL_698512, EPI_ISL_698513, EPI_ISL_698514, EPI_ISL_698515, EPI_ISL_698516, EPI_ISL_698518, EPI_ISL_698520, EPI_ISL_698530, EPI_ISL_698531, EPI_ISL_698533, EPI_ISL_698553, EPI_ISL_698554, EPI_ISL_698555, EPI_ISL_698566, EPI_ISL_698569, EPI_ISL_698570, EPI_ISL_698571, EPI_ISL_698572, EPI_ISL_698573, EPI_ISL_698575, EPI_ISL_698576, EPI_ISL_698577, EPI_ISL_698595, EPI_ISL_698596, EPI_ISL_698597, EPI_ISL_698598, EPI_ISL_698599, EPI_ISL_698600, EPI_ISL_698601, EPI_ISL_698602, EPI_ISL_698603, EPI_ISL_698604, EPI_ISL_698605, EPI_ISL_698606, EPI_ISL_698617, EPI_ISL_698618, EPI_ISL_698619, EPI_ISL_698621, EPI_ISL_698626, EPI_ISL_698628, EPI_ISL_698631, EPI_ISL_698632, EPI_ISL_698635, EPI_ISL_698636, EPI_ISL_698638, EPI_ISL_698639, EPI_ISL_698640, EPI_ISL_698641, EPI_ISL_698642, EPI_ISL_698643, EPI_ISL_698644, EPI_ISL_698646, EPI_ISL_698647, EPI_ISL_698649, EPI_ISL_698650, EPI_ISL_698651, EPI_ISL_698652, EPI_ISL_698653, EPI_ISL_698654, EPI_ISL_698655, EPI_ISL_698656, EPI_ISL_698657, EPI_ISL_698659, EPI_ISL_698663, EPI_ISL_698666, EPI_ISL_698667, EPI_ISL_698668, EPI_ISL_698669, EPI_ISL_698672, EPI_ISL_698674, EPI_ISL_698675, EPI_ISL_698676, EPI_ISL_698677, EPI_ISL_698678, EPI_ISL_698679, EPI_ISL_698680, EPI_ISL_698682, EPI_ISL_698683, EPI_ISL_698684, EPI_ISL_698687, EPI_ISL_698688, EPI_ISL_698691, EPI_ISL_698693, EPI_ISL_698695, EPI_ISL_698697, EPI_ISL_698698, EPI_ISL_698700, EPI_ISL_698701, EPI_ISL_698704, EPI_ISL_698706, EPI_ISL_698717, EPI_ISL_698719, EPI_ISL_698720, EPI_ISL_698721, EPI_ISL_698722, EPI_ISL_698731, EPI_ISL_698732, EPI_ISL_698735, EPI_ISL_698739, EPI_ISL_698741, EPI_ISL_698742, EPI_ISL_698743, EPI_ISL_698746, EPI_ISL_698748, EPI_ISL_698765, EPI_ISL_698766, EPI_ISL_698767, EPI_ISL_698768, EPI_ISL_698769, EPI_ISL_698770, EPI_ISL_698771, EPI_ISL_698773, EPI_ISL_698774, EPI_ISL_698775, EPI_ISL_698779, EPI_ISL_698780, EPI_ISL_698781, EPI_ISL_698782, EPI_ISL_698783, EPI_ISL_698785, EPI_ISL_698786, EPI_ISL_698787, EPI_ISL_698790, EPI_ISL_698791, EPI_ISL_698792, EPI_ISL_698799, EPI_ISL_698801, EPI_ISL_698802, EPI_ISL_698804, EPI_ISL_698806, EPI_ISL_698807, EPI_ISL_698808, EPI_ISL_698809, EPI_ISL_698810, EPI_ISL_698813, EPI_ISL_698816, EPI_ISL_698817, EPI_ISL_698818, EPI_ISL_698819, EPI_ISL_698822, EPI_ISL_698823, EPI_ISL_698824, EPI_ISL_698825, EPI_ISL_698826, EPI_ISL_698828, EPI_ISL_698829, EPI_ISL_698830, EPI_ISL_698831, EPI_ISL_698832, EPI_ISL_698835, EPI_ISL_698837, EPI_ISL_698838, EPI_ISL_698839, EPI_ISL_698841, EPI_ISL_698845, EPI_ISL_698846, EPI_ISL_698847, EPI_ISL_698848, EPI_ISL_698850, EPI_ISL_698852, EPI_ISL_698854, EPI_ISL_698855, EPI_ISL_698856, EPI_ISL_698858, EPI_ISL_698861, EPI_ISL_698863, EPI_ISL_698865, EPI_ISL_698866, EPI_ISL_698868, EPI_ISL_698869, EPI_ISL_698871, EPI_ISL_698872, EPI_ISL_698873, EPI_ISL_698874, EPI_ISL_698878, EPI_ISL_698879, EPI_ISL_698881, EPI_ISL_698882, EPI_ISL_698883, EPI_ISL_698884, EPI_ISL_698885, EPI_ISL_698887, EPI_ISL_698888, EPI_ISL_698890, EPI_ISL_698891, EPI_ISL_698893, EPI_ISL_698894, EPI_ISL_698896, EPI_ISL_698900, EPI_ISL_698902, EPI_ISL_698905, EPI_ISL_698906, EPI_ISL_698907, EPI_ISL_698908, EPI_ISL_698909, EPI_ISL_698912, EPI_ISL_698913, EPI_ISL_698915, EPI_ISL_698918, EPI_ISL_698920, EPI_ISL_698921, EPI_ISL_698923, EPI_ISL_698924, EPI_ISL_698925, EPI_ISL_698926, EPI_ISL_698927, EPI_ISL_698928, EPI_ISL_698929, EPI_ISL_698930, EPI_ISL_698931, EPI_ISL_698933, EPI_ISL_698934, EPI_ISL_698937, EPI_ISL_698939, EPI_ISL_698940, EPI_ISL_698942, EPI_ISL_698944, EPI_ISL_698946, EPI_ISL_698947, EPI_ISL_698948, EPI_ISL_698949, EPI_ISL_698951, EPI_ISL_698952, EPI_ISL_698953, EPI_ISL_698954, EPI_ISL_698955, EPI_ISL_698956, EPI_ISL_698957, EPI_ISL_698958, EPI_ISL_698961, EPI_ISL_698962, EPI_ISL_698963, EPI_ISL_698964, EPI_ISL_698965, EPI_ISL_698966, EPI_ISL_698967, EPI_ISL_698969, EPI_ISL_698970, EPI_ISL_698972, EPI_ISL_698974, EPI_ISL_698975, EPI_ISL_698976, EPI_ISL_708828, EPI_ISL_708830, EPI_ISL_708832, EPI_ISL_708833, EPI_ISL_708837, EPI_ISL_708838			
see above	Group 42 (G42) Healthcare, Abu Dhabi, United Arab Emirates; Department of Health, The United Arab Emirates	G42 Healthcare	Ashish Koshy; Budoor Alqarni; Denghui Liu; Fang Chen; Hanif Khalak; Huanming Yang; Javier Quilez; Jian Wang; Junhua Li; Ke Liang; Long Lin; Mohammed Saifuddin Fasihuddin; Nan Qiao; Nawal Ahmed Mohamed Al Kaabi; Pauline Ogrodzki; Pei Wu; Peng Xiao; Pengjuan Liu; Rong Liu; Sally Mahmoud; Siyang Liu; Stephen S. Francis; Tao Ma; Vinay Kusuma; Walid Abbas Zaher; Weibin Liu; Wenjun He; Xavier Anton; Xin Jin; Xin Meng; Xinyu Huang; Xun Xu; Zhaorong Yuan
EPI_ISL_1712867	Ministry of Public Health / Hamad Medical Corporation	Biomedical Research Center (BRC), Qatar University / Qatar Genome Project (QGP)	Asmaa A. Al-Thani. MOPH and HMC: Abdullatif Al-Khal; BRC: Fatiha M. Benslimane; Chadi Saad; Dana Al-Batesh; Dina Elgakhlab QGP: Fatima H. Al-Kuwari; Einas A. E. Al-Kuwari; Hadi M. Yassine; Hamad E. Al-Romaihi; Hamda Alromaihi; Heba A. Al-Khatib; Mashael A. Al-Bader; Mohammed Al-Thani; Muna A. S. Al-Maslamani; Oal Al-Jamal; Peter V. Coyle; Reham A. El-Kahlout. QBB: Tasneem Al-Hamad; Roberto Bertollini; Salih Al-Marri
EPI_ISL_1714662, EPI_ISL_1714744	Ministry of Public Health / Hamad Medical Corporation	Weill Cornell Medical College - Qatar (WCM-Q), Genomics Core Laboratory / Qatar Genome Project (QGP)	Chadi Saad MOPH and HMC: Abdullatif Al-Khal; Dina Elgakhlab; Einas A. E. Al-Kuwari; Hamad E. Al-Romaihi; Hamda Alromaihi; Joel A Malek. QGP: Fatima H. Al-Kuwari; Laith Abu-Raddad; Mashael A. Al-Bader; Meryem Bensaad; Mohammed Al-Thani; Muna A. S. Al-Maslamani; Peter V. Coyle; Reham A. El-Kahlout. QBB: Tasneem Al-Hamad; Roberto Bertollini; Salih Al-Marri; Shameem Younuskunju; WCMQ: Ayeda A. Ahmed; Yasmin Mohamoud
EPI_ISL_520687, EPI_ISL_520688, EPI_ISL_520689, EPI_ISL_520690, EPI_ISL_520691, EPI_ISL_520692, EPI_ISL_520693, EPI_ISL_520694			
see above	Mohammed Bin Rashid University of Medicine and Health Sciences	Al Jalila Genomics Center	Abdulmajeed Alkhaja; Abiola Catherine Senok; Ahmad Abou Tayoun; Alawi Alsheikh-Ali; Divinlal Harilal; Hamda Khansaheb; Hanan Al Suwaidi; Mohammed Uddin; Norbert Nowotny; Qutayba Hamid; Rabih Halwani; Rifat Hamoudi; Rupa Murthy Varghese; Sathishkumar Ramaswamy; Tom Loney; Zulfa Omar Deesi
EPI_ISL_491976, EPI_ISL_491978, EPI_ISL_491979, EPI_ISL_491980, EPI_ISL_491981, EPI_ISL_491982, EPI_ISL_491983, EPI_ISL_491984			
see above	Oman-NIC	Department of Microbiology and Immunology-SQUH	Abdulla Balkhair; Ahlam Al-Amri; Aisha Al-Amri; Aisha Al-Busaidi; Amina Al Jardani; Fahad Zadjali; Fatma BaAlawi; Hamida AL Barwani; Hanan Al-kindi; Intisar Al-Shukri; Khulood Al-Mammary; Mohammed Al-Tobi; Samiha Al Kharusi; Samira Al-Maruqi; Zeyana AL-Dahmani

We gratefully acknowledge the following Authors from the Originating laboratories responsible for obtaining the specimens, as well as the Submitting laboratories where the genome data were generated and shared via GISAID, on which this research is based.

All Submitters of data may be contacted directly via www.gisaid.org

Authors are sorted alphabetically.

Acknowledgement EPI_SET Identifier: EPI_SET_20220314ze

Accession ID	Originating Laboratory	Submitting Laboratory	Authors
EPI_ISL_528691, EPI_ISL_528694, EPI_ISL_528695, EPI_ISL_528696, EPI_ISL_528700, EPI_ISL_528702, EPI_ISL_528708			
see above	Alsafar - Khalifa University Abu Dhabi	Alsafar - Khalifa University Abu Dhabi	Andreas Henschel; Ernesto Damiani; Gihan Daw Elbait; Guan Tay; Habiba Alsafar; Rifat Hamoudi; Samuel Feng
EPI_ISL_632265, EPI_ISL_632285	Communicable Disease Laboratory, Public Health Directorate	Communicable Disease Laboratory, Public Health Directorate	AlAbbas, Z.; AlHujairi, Z.; AlTaif, Z.; AlWasti, H.
EPI_ISL_698105, EPI_ISL_698106, EPI_ISL_698107, EPI_ISL_698108, EPI_ISL_698109, EPI_ISL_698110, EPI_ISL_698111, EPI_ISL_698112, EPI_ISL_698113, EPI_ISL_698114, EPI_ISL_698115, EPI_ISL_698116, EPI_ISL_698120, EPI_ISL_698121, EPI_ISL_698122, EPI_ISL_698123, EPI_ISL_698124, EPI_ISL_698125, EPI_ISL_698126, EPI_ISL_698127, EPI_ISL_698128, EPI_ISL_698130, EPI_ISL_698131, EPI_ISL_698132, EPI_ISL_698135, EPI_ISL_698138, EPI_ISL_698139, EPI_ISL_698140, EPI_ISL_698141, EPI_ISL_698142, EPI_ISL_698143, EPI_ISL_698146, EPI_ISL_698147, EPI_ISL_698149, EPI_ISL_698151, EPI_ISL_698152, EPI_ISL_698154, EPI_ISL_698212, EPI_ISL_698977, EPI_ISL_698978, EPI_ISL_698981, EPI_ISL_698985, EPI_ISL_698986, EPI_ISL_698987, EPI_ISL_698988, EPI_ISL_698990, EPI_ISL_698992, EPI_ISL_698993, EPI_ISL_698995, EPI_ISL_698999, EPI_ISL_699000, EPI_ISL_699003, EPI_ISL_699004, EPI_ISL_699005, EPI_ISL_699006, EPI_ISL_699007, EPI_ISL_699008, EPI_ISL_699012, EPI_ISL_699013, EPI_ISL_699014, EPI_ISL_699015, EPI_ISL_699017, EPI_ISL_699019, EPI_ISL_699020, EPI_ISL_699021, EPI_ISL_699023, EPI_ISL_699024, EPI_ISL_699026, EPI_ISL_699041, EPI_ISL_699091, EPI_ISL_699113, EPI_ISL_699114, EPI_ISL_699116, EPI_ISL_699117, EPI_ISL_699119, EPI_ISL_699120, EPI_ISL_699121, EPI_ISL_699123, EPI_ISL_699124, EPI_ISL_699125, EPI_ISL_699126, EPI_ISL_699127, EPI_ISL_699129, EPI_ISL_699130, EPI_ISL_699131, EPI_ISL_699132, EPI_ISL_699133, EPI_ISL_699135, EPI_ISL_699136, EPI_ISL_699137, EPI_ISL_699138, EPI_ISL_699140, EPI_ISL_699141, EPI_ISL_699142, EPI_ISL_699144, EPI_ISL_699145, EPI_ISL_699148, EPI_ISL_699149, EPI_ISL_699152, EPI_ISL_699156, EPI_ISL_699161, EPI_ISL_708827, EPI_ISL_708829			
see above	Group 42 (G42) Healthcare, Abu Dhabi, United Arab Emirates; Department of Health, The United Arab Emirates	G42 Healthcare	Ashish Koshy; Budoor Alqarni; Denghui Liu; Fang Chen; Hanif Khalak; Huanming Yang; Javier Quilez; Jian Wang; Junhua Li; Ke Liang; Long Lin; Mohammed Saifuddin Fasihuddin; Nan Qiao; Nawal Ahmed Mohamed Al Kaabi; Pauline Ogrodzki; Pei Wu; Peng Xiao; Pengjuan Liu; Rong Liu; Sally Mahmoud; Siyang Liu; Stephen S. Francis; Tao Ma; Vinay Kusuma; Walid Abbas Zaher; Weibin Liu; Wenjun He; Xavier Anton; Xin Jin; Xin Meng; Xinyu Huang; Xun Xu; Zhaorong Yuan
EPI_ISL_1712818, EPI_ISL_1712820, EPI_ISL_1712824, EPI_ISL_1712850	Ministry of Public Health / Hamad Medical Corporation	Biomedical Research Center (BRC), Qatar University / Qatar Genome Project (QGP)	Asmaa A. Al-Thani. MOPH and HMC: Abdullatif Al-Khal; BRC: Fatiha M. Benslimane; Chadi Saad; Dana Al-Batesh; Dina Elgakhlab QGP: Fatima H. Al-Kuwari; Einas A. E. Al-Kuwari; Hadi M. Yassine; Hamad E. Al-Romaihi; Hamda Alromaihi; Heba A. Al-Khatib; Mashael A. Al-Bader; Mohammed Al-Thani; Muna A. S. Al-Maslamani; Oal Al-Jamal; Peter V. Coyle; Reham A. El-Kahlout. QBB: Tasneem Al-Hamad; Roberto Bertollini; Salih Al-Marri
EPI_ISL_1714052, EPI_ISL_1714057, EPI_ISL_1714071, EPI_ISL_1714072, EPI_ISL_1714073, EPI_ISL_1714075, EPI_ISL_1714149, EPI_ISL_1714152, EPI_ISL_1714163, EPI_ISL_1714169, EPI_ISL_1714171, EPI_ISL_1714172, EPI_ISL_1714175, EPI_ISL_1714178, EPI_ISL_1714187, EPI_ISL_1714188, EPI_ISL_1714190, EPI_ISL_1714192, EPI_ISL_1714193, EPI_ISL_1714194, EPI_ISL_1714197, EPI_ISL_1714198, EPI_ISL_1714199, EPI_ISL_1714200, EPI_ISL_1714201, EPI_ISL_1714204, EPI_ISL_1714205, EPI_ISL_1714206, EPI_ISL_1714208, EPI_ISL_1714215, EPI_ISL_1714216, EPI_ISL_1714217, EPI_ISL_1714218, EPI_ISL_1714220, EPI_ISL_1714221, EPI_ISL_1714223, EPI_ISL_1714224, EPI_ISL_1714225, EPI_ISL_1714227, EPI_ISL_1714233, EPI_ISL_1714234, EPI_ISL_1714235, EPI_ISL_1714236, EPI_ISL_1714238, EPI_ISL_1714239, EPI_ISL_1714240, EPI_ISL_1714246, EPI_ISL_1714247, EPI_ISL_1714249, EPI_ISL_1714250, EPI_ISL_1714252, EPI_ISL_1714253, EPI_ISL_1714254, EPI_ISL_1714255, EPI_ISL_1714258, EPI_ISL_1714259, EPI_ISL_1714260, EPI_ISL_1714261, EPI_ISL_1714263, EPI_ISL_1714265, EPI_ISL_1714266, EPI_ISL_1714267, EPI_ISL_1714269, EPI_ISL_1714271, EPI_ISL_1714273, EPI_ISL_1714274, EPI_ISL_1714355, EPI_ISL_1714362, EPI_ISL_1714550, EPI_ISL_1714551, EPI_ISL_1714555, EPI_ISL_1714556, EPI_ISL_1714557, EPI_ISL_1714558, EPI_ISL_1714559, EPI_ISL_1714560, EPI_ISL_1714561, EPI_ISL_1714562, EPI_ISL_1714564, EPI_ISL_1714565, EPI_ISL_1714566, EPI_ISL_1714567, EPI_ISL_1714568, EPI_ISL_1714569, EPI_ISL_1714570, EPI_ISL_1714571, EPI_ISL_1714574, EPI_ISL_1714575, EPI_ISL_1714578, EPI_ISL_1714579, EPI_ISL_1714580, EPI_ISL_1714581, EPI_ISL_1714582, EPI_ISL_1714583, EPI_ISL_1714584, EPI_ISL_1714585, EPI_ISL_1714586, EPI_ISL_1714587, EPI_ISL_1714588, EPI_ISL_1714590, EPI_ISL_1714591, EPI_ISL_1714592, EPI_ISL_1714593, EPI_ISL_1714595, EPI_ISL_1714596, EPI_ISL_1714598, EPI_ISL_1714601, EPI_ISL_1714602, EPI_ISL_1714603, EPI_ISL_1714604, EPI_ISL_1714605, EPI_ISL_1714606, EPI_ISL_1714607, EPI_ISL_1714608, EPI_ISL_1714609, EPI_ISL_1714612, EPI_ISL_1714613, EPI_ISL_1714614, EPI_ISL_1714615, EPI_ISL_1714616, EPI_ISL_1714617, EPI_ISL_1714618, EPI_ISL_1714619, EPI_ISL_1714620, EPI_ISL_1714621, EPI_ISL_1714622, EPI_ISL_1714623, EPI_ISL_1714624, EPI_ISL_1714625, EPI_ISL_1714626, EPI_ISL_1714627, EPI_ISL_1714628, EPI_ISL_1714629, EPI_ISL_1714633, EPI_ISL_1714636, EPI_ISL_1714637, EPI_ISL_1714638, EPI_ISL_1714639, EPI_ISL_1714640, EPI_ISL_1714641, EPI_ISL_1714642, EPI_ISL_1714643, EPI_ISL_1714644, EPI_ISL_1714661			
see above	Ministry of Public Health / Hamad Medical Corporation	Weill Cornell Medical College - Qatar (WCM-Q), Genomics Core Laboratory / Qatar Genome Project (QGP)	Chadi Saad MOPH and HMC: Abdullatif Al-Khal; Dina Elgakhlab; Einas A. E. Al-Kuwari; Hamad E. Al-Romaihi; Hamda Alromaihi; Joel A Malek. QGP: Fatima H. Al-Kuwari; Laith Abu-Raddad; Mashael A. Al-Bader; Meryem Bensaad; Mohammed Al-Thani; Muna A. S. Al-Maslamani; Peter V. Coyle; Reham A. El-Kahlout. QBB: Tasneem Al-Hamad; Roberto Bertollini; Salih Al-Marri; Shameem Younuskunju; WCMQ: Ayeda A. Ahmed; Yasmin Mohamoud
EPI_ISL_520676, EPI_ISL_520677, EPI_ISL_520678, EPI_ISL_520679, EPI_ISL_520695, EPI_ISL_520696, EPI_ISL_520697, EPI_ISL_520699, EPI_ISL_520700, EPI_ISL_520701, EPI_ISL_520702, EPI_ISL_520703, EPI_ISL_520704, EPI_ISL_520705, EPI_ISL_520706, EPI_ISL_520707, EPI_ISL_520708, EPI_ISL_520709, EPI_ISL_520711, EPI_ISL_520712, EPI_ISL_520713, EPI_ISL_520714, EPI_ISL_520715, EPI_ISL_523952			
see above	Mohammed Bin Rashid University of Medicine and Health Sciences	Al Jalila Genomics Center	Abdulmajeed Alkhaja; Abiola Catherine Senok; Ahmad Abou Tayoun; Alawi Alsheikh-Ali; Divinlal Harilal; Hamda Khansaheb; Hanan Al Suwaidi; Mohammed Uddin; Norbert Nowotny; Qutayba Hamid; Rabih Halwani; Rifat Hamoudi; Rupa Murthy Varghese; Sathishkumar Ramaswamy; Tom Loney; Zulfa Omar Deesi
EPI_ISL_1795043, EPI_ISL_1795044, EPI_ISL_1795045, EPI_ISL_1795047	Oman-NIC	Department of Microbiology and Immunology-SQUH	Abdulla Balkhair; Ahlam Al-Amri; Aisha Al-Amri; Aisha Al-Busaidi; Amina Al Jardani; Fahad Zadjali; Fatma BaAlawi; Hamida AL Barwani; Hanan Al-kindi; Intisar Al-Shukri; Khulood Al-Mammary; Mohammed Al-Tobi; Samiha Al Kharusi; Samira Al-Maruqi; Zeyana AL-Dahmani
EPI_ISL_491988, EPI_ISL_491989, EPI_ISL_491993, EPI_ISL_491995, EPI_ISL_491996, EPI_ISL_491998, EPI_ISL_491999, EPI_ISL_492002, EPI_ISL_492003, EPI_ISL_492008, EPI_ISL_492009, EPI_ISL_492010, EPI_ISL_492011, EPI_ISL_492014, EPI_ISL_492016, EPI_ISL_492017, EPI_ISL_492019, EPI_ISL_492020, EPI_ISL_492021, EPI_ISL_492023, EPI_ISL_492025			
see above	Oman-NIC	Department of Microbiology and Immunology-SQUH	Abdulla Balkhair; Ahlam Al-Amri; Aisha Al-Amri; Aisha Al-Busaidi; Amina Al Jardani; Fahad Zadjali; Fatma BaAlawi; Hamida AL Barwani; Hanan Al-kindi; Intisar Al-Shukri; Khulood Al-Mammary; Mohammed Al-Tobi; Samiha Al Kharusi; Samira Al-Maruqi; Zeyana AL-Dahmani
EPI_ISL_1819073, EPI_ISL_1819074	Oman-NIC	Oman-National Influenza Center-Department of Microbiology and Immunology-SQUH	Abdulla Balkhair; Ahlam Al-Amri; Aisha Al-Amri; Aisha Al-Busaidi; Amina Al Jardani; Fahad Zadjali; Fatma BaAlawi; Hamida AL Barwani; Hanan Al-kindi; Intisar Al-Shukri; Khulood Al-Mammary; Mohammed Al-Tobi; Samiha Al Kharusi; Samira Al-Maruqi; Zeyana AL-Dahmani
EPI_ISL_525426, EPI_ISL_525428, EPI_ISL_525429, EPI_ISL_525466	Oman-National Influenza Center	Biotechnology & OMICs Laboratory	Abdul Latif Khan; Adil Al-Wahaibi; Adil Khan; Ahlam Al-Amri; Ahmed Al-Harrasi; Ahmed Al-Rawahi; Aisha Al-Amri; Aisha Al-Busaidi; Amina Al-Jardani; Hanan Al-Kindi; Intisar Al-Shukri; Sajjad Asaf; Samiha Al-Kharusi; Samira Al-Mahruqi; Seif Al-Abri; Seif Al-Abri.
EPI_ISL_518824, EPI_ISL_518825, EPI_ISL_518826, EPI_ISL_518827, EPI_ISL_518828, EPI_ISL_518829, EPI_ISL_518830, EPI_ISL_518831, EPI_ISL_518832, EPI_ISL_518833, EPI_ISL_518834, EPI_ISL_518835, EPI_ISL_518836, EPI_ISL_518837, EPI_ISL_518839, EPI_ISL_518840, EPI_ISL_518842			
see above	Oman-National Influenza Center	Biotechnology & OMICs Laboratory, Natural & Medical Sciences Research Center, University of Nizwa	Abdul Latif Khan; Adil Al-Wahaibi; Adil Khan; Ahlam Al-Amri; Ahmed Al-Harrasi; Ahmed Al-Rawahi; Aisha Al-Amri; Aisha Al-Busaidi; Amina Al-Jardani; Hanan Al-Kindi; Intisar Al-Shukri; Sajjad Asaf; Samiha Al-Kharusi; Samira Al-Mahruqi; Seif Al-Abri; Seif Al-Abri.
EPI_ISL_636972	Pathogen Genomics Lab King Abdullah University of Science and Technology (KAUST)	Pathogen Genomics Lab King Abdullah University of Science and Technology (KAUST)	Afrah Alsomali; Amanda Ooi; Amit Kumar Subudhi; Anwar Hashem; Arnab Pain; Asim Khogeer; Fadwa Alofi; Fathia Ben Rached; Kahled Algithami; Luke Esau; Naif Almontashiri; Raeece Naeem; Rahul P Salunke; Sara Mfarrej; Sharif Hala
EPI_ISL_512878, EPI_ISL_512879, EPI_ISL_512880, EPI_ISL_512881, EPI_ISL_512884, EPI_ISL_512885, EPI_ISL_512886, EPI_ISL_512887, EPI_ISL_512888, EPI_ISL_512896, EPI_ISL_512903, EPI_ISL_512905, EPI_ISL_513002, EPI_ISL_513003, EPI_ISL_513005, EPI_ISL_513009, EPI_ISL_513010, EPI_ISL_513012, EPI_ISL_513013, EPI_ISL_513014, EPI_ISL_513016, EPI_ISL_513018, EPI_ISL_513020, EPI_ISL_513021, EPI_ISL_513022, EPI_ISL_513023, EPI_ISL_513024, EPI_ISL_513025, EPI_ISL_513026, EPI_ISL_513027, EPI_ISL_513028, EPI_ISL_513029, EPI_ISL_513030, EPI_ISL_513033, EPI_ISL_513034, EPI_ISL_513037, EPI_ISL_513038, EPI_ISL_513039, EPI_ISL_513040, EPI_ISL_513063, EPI_ISL_513066, EPI_ISL_513067, EPI_ISL_513068, EPI_ISL_513069, EPI_ISL_513070, EPI_ISL_513073, EPI_ISL_513074, EPI_ISL_513075, EPI_ISL_513076, EPI_ISL_513077, EPI_ISL_513078, EPI_ISL_513079, EPI_ISL_513080, EPI_ISL_513081, EPI_ISL_513082, EPI_ISL_513083, EPI_ISL_513085, EPI_ISL_513086, EPI_ISL_513087, EPI_ISL_513089, EPI_ISL_513090, EPI_ISL_513091, EPI_ISL_513092, EPI_ISL_513093, EPI_ISL_513094, EPI_ISL_513095, EPI_ISL_513096, EPI_ISL_513097, EPI_ISL_513098, EPI_ISL_513099, EPI_ISL_513100, EPI_ISL_513101, EPI_ISL_513102, EPI_ISL_513103, EPI_ISL_513104, EPI_ISL_513105, EPI_ISL_513106, EPI_ISL_513107, EPI_ISL_513108, EPI_ISL_513109, EPI_ISL_513111, EPI_ISL_513112, EPI_ISL_513113, EPI_ISL_513114, EPI_ISL_513115, EPI_ISL_513116, EPI_ISL_513117, EPI_ISL_513120, EPI_ISL_513190, EPI_ISL_513191, EPI_ISL_513192, EPI_ISL_513193, EPI_ISL_513194, EPI_ISL_513197, EPI_ISL_513199, EPI_ISL_513200, EPI_ISL_513201, EPI_ISL_513202, EPI_ISL_513204, EPI_ISL_513207, EPI_ISL_513212, EPI_ISL_636965, EPI_ISL_636967, EPI_ISL_636968, EPI_ISL_677967, EPI_ISL_677969, EPI_ISL_677972, EPI_ISL_677974, EPI_ISL_677975, EPI_ISL_677977, EPI_ISL_677978, EPI_ISL_677979, EPI_ISL_677980, EPI_ISL_677981, EPI_ISL_677982, EPI_ISL_677983, EPI_ISL_677984, EPI_ISL_677985, EPI_ISL_677986, EPI_ISL_677987, EPI_ISL_677988, EPI_ISL_677989, EPI_ISL_677990, EPI_ISL_678003, EPI_ISL_678011, EPI_ISL_678012, EPI_ISL_678013, EPI_ISL_678014, EPI_ISL_678015, EPI_ISL_678016, EPI_ISL_678017, EPI_ISL_678018, EPI_ISL_678019, EPI_ISL_678020, EPI_ISL_678021, EPI_ISL_678022, EPI_ISL_678023, EPI_ISL_678024, EPI_ISL_678025, EPI_ISL_678152, EPI_ISL_678153, EPI_ISL_678183, EPI_ISL_678187, EPI_ISL_678188, EPI_ISL_678189, EPI_ISL_678190, EPI_ISL_678191, EPI_ISL_678192, EPI_ISL_678193, EPI_ISL_678194, EPI_ISL_678195, EPI_ISL_678196, EPI_ISL_678197, EPI_ISL_678198, EPI_ISL_678199, EPI_ISL_678200, EPI_ISL_678201, EPI_ISL_678202, EPI_ISL_678203, EPI_ISL_678204, EPI_ISL_678205, EPI_ISL_678206, EPI_ISL_678207, EPI_ISL_678208, EPI_ISL_678209, EPI_ISL_678210, EPI_ISL_678237			
see above	Pathogen Genomics Lab King Abdullah University of Science and Technology(KAUST)	Pathogen Genomics Lab King Abdullah University of Science and Technology(KAUST)	Abbas Al Mutair; Abdulaziz Alahmadi; Afrah Alsomali; Ahmad Bakur Mahmoud; Amanda Ooi; Amit Kumar Subudhi; Anwar Hashem; Arnab Pain; Asim Khogeer; Awad A-Omari; Awad Al-Omari; Fadwa Alofi; Fathia Ben Rached; Jumana Taha; Kahled Algithami; Luke Esau; Muhammad Shuaib; Naif Almontashiri; Olga Douvropoulou; Raeece Naeem; Rahul P Salunke; Raushan Nugmanova; Samer Salih; Sara Mfarrej; Sharif Hala

We gratefully acknowledge the following Authors from the Originating laboratories responsible for obtaining the specimens, as well as the Submitting laboratories where the genome data were generated and shared via GISAID, on which this research is based.

All Submitters of data may be contacted directly via www.gisaid.org

Authors are sorted alphabetically.

Acknowledgement EPI_SET Identifier: EPI_SET_20220314tg

Accession ID	Originating Laboratory	Submitting Laboratory	Authors
EPI_ISL_528703, EPI_ISL_528705, EPI_ISL_528707	Alsafar - Khalifa University Abu Dhabi	Alsafar - Khalifa University Abu Dhabi	Andreas Henschel; Ernesto Damiani; Gihan Daw Elbait; Guan Tay; Habiba Alsafar; Rifat Hamoudi; Samuel Feng
EPI_ISL_859561, EPI_ISL_859562, EPI_ISL_859563, EPI_ISL_859564, EPI_ISL_859565, EPI_ISL_859566, EPI_ISL_859567, EPI_ISL_859568, EPI_ISL_859570, EPI_ISL_859571, EPI_ISL_859572, EPI_ISL_859574, EPI_ISL_859575, EPI_ISL_859578, EPI_ISL_859579, EPI_ISL_859580, EPI_ISL_859581, EPI_ISL_859582, EPI_ISL_859584, EPI_ISL_859585, EPI_ISL_859586, EPI_ISL_859587, EPI_ISL_859588, EPI_ISL_859589, EPI_ISL_859591, EPI_ISL_859593, EPI_ISL_859594, EPI_ISL_859595, EPI_ISL_859596, EPI_ISL_859597, EPI_ISL_859598, EPI_ISL_859599, EPI_ISL_859600, EPI_ISL_859604, EPI_ISL_859606, EPI_ISL_859609, EPI_ISL_859611, EPI_ISL_859612, EPI_ISL_859613, EPI_ISL_859615, EPI_ISL_859616, EPI_ISL_859617, EPI_ISL_859619, EPI_ISL_859621, EPI_ISL_859622, EPI_ISL_859623, EPI_ISL_859625, EPI_ISL_859627, EPI_ISL_859629, EPI_ISL_859630, EPI_ISL_859631, EPI_ISL_859632, EPI_ISL_859634, EPI_ISL_859635, EPI_ISL_859637, EPI_ISL_859639, EPI_ISL_859640, EPI_ISL_859641, EPI_ISL_859642, EPI_ISL_859643, EPI_ISL_859646, EPI_ISL_859648, EPI_ISL_859649, EPI_ISL_859651, EPI_ISL_859652, EPI_ISL_859653, EPI_ISL_859654, EPI_ISL_859656, EPI_ISL_859657, EPI_ISL_859659, EPI_ISL_859660, EPI_ISL_859662, EPI_ISL_859663, EPI_ISL_859664, EPI_ISL_859665, EPI_ISL_859667, EPI_ISL_859668, EPI_ISL_859670, EPI_ISL_859671, EPI_ISL_859673, EPI_ISL_859674, EPI_ISL_859676, EPI_ISL_859677, EPI_ISL_859678, EPI_ISL_859680, EPI_ISL_859681			
see above	BTC, Khalifa University	BTC, Khalifa University	Al Safar et al
EPI_ISL_632262, EPI_ISL_632263, EPI_ISL_632266, EPI_ISL_632267, EPI_ISL_632284	Communicable Disease Laboratory, Public Health Directorate	Communicable Disease Laboratory, Public Health Directorate	AlAbbas, Z.; AlHujairi, Z.; AlTaif, Z.; AlWasti, H.
EPI_ISL_485401	Communicable Disease Laboratory, Public Health Directorate	Communicable Disease Laboratory, Public Health Directorate	Al-Taif; Al-Wasti, H.; Z. and Shehab, F.; Zaed, A.
EPI_ISL_698191, EPI_ISL_699027, EPI_ISL_699028, EPI_ISL_699029, EPI_ISL_699030, EPI_ISL_699032, EPI_ISL_699033, EPI_ISL_699035, EPI_ISL_699036, EPI_ISL_699037, EPI_ISL_699038, EPI_ISL_699039, EPI_ISL_699040, EPI_ISL_699042, EPI_ISL_699043, EPI_ISL_699044, EPI_ISL_699045, EPI_ISL_699046, EPI_ISL_699047, EPI_ISL_699048, EPI_ISL_699050, EPI_ISL_699054, EPI_ISL_699055, EPI_ISL_699056, EPI_ISL_699057, EPI_ISL_699058, EPI_ISL_699059, EPI_ISL_699060, EPI_ISL_699061, EPI_ISL_699062, EPI_ISL_699063, EPI_ISL_699064, EPI_ISL_699065, EPI_ISL_699067, EPI_ISL_699068, EPI_ISL_699069, EPI_ISL_699072, EPI_ISL_699074, EPI_ISL_699076, EPI_ISL_699079, EPI_ISL_699081, EPI_ISL_699083, EPI_ISL_699084, EPI_ISL_699086, EPI_ISL_699087, EPI_ISL_699089, EPI_ISL_699090, EPI_ISL_699092, EPI_ISL_699094, EPI_ISL_699095, EPI_ISL_699100, EPI_ISL_699102, EPI_ISL_699103, EPI_ISL_699104, EPI_ISL_699105, EPI_ISL_699107, EPI_ISL_699108, EPI_ISL_699110, EPI_ISL_699112			
see above	Group 42 (G42) Healthcare, Abu Dhabi, United Arab Emirates; Department of Health, The United Arab Emirates	G42 Healthcare	Ashish Koshy; Budoor Alqarni; Denghui Liu; Fang Chen; Hanif Khalak; Huanming Yang; Javier Quilez; Jian Wang; Junhua Li; Ke Liang; Long Lin; Mohammed Saifuddin Fasihuddin; Nan Qiao; Nawal Ahmed Mohamed Al Kaabi; Pauline Ogrodzki; Pei Wu; Peng Xiao; Pengjuan Liu; Rong Liu; Sally Mahmoud; Siyang Liu; Stephen S. Francis; Tao Ma; Vinay Kusuma; Walid Abbas Zaher; Weibin Liu; Wenjun He; Xavier Anton; Xin Jin; Xin Meng; Xinyu Huang; Xun Xu; Zhaorong Yuan
EPI_ISL_1713290, EPI_ISL_1713297, EPI_ISL_1713300, EPI_ISL_1713301, EPI_ISL_1713304, EPI_ISL_1713310, EPI_ISL_1713342, EPI_ISL_1713356			
see above	Ministry of Public Health / Hamad Medical Corporation	Biomedical Research Center (BRC), Qatar University / Qatar Genome Project (QGP)	Asmaa A. Al-Thani. MOPH and HMC: Abdullatif Al-Khal; BRC: Fatiha M. Benslimane; Chadi Saad; Dana Al-Batesh; Dina Elgakhlab QGP: Fatima H. Al-Kuwari; Einas A. E. Al-Kuwari; Hadi M. Yassine; Hamad E. Al-Romaihi; Hamda Alromaihi; Heba A. Al-Khatib; Mashael A. Al-Bader; Mohammed Al-Thani; Muna A. S. Al-Maslamani; Oal Al-Jamal; Peter V. Coyle; Reham A. El-Kahlout. QBB: Tasneem Al-Hamad; Roberto Bertollini; Salih Al-Marri
EPI_ISL_1714046, EPI_ISL_1714164, EPI_ISL_1714165, EPI_ISL_1714173, EPI_ISL_1714176, EPI_ISL_1714177, EPI_ISL_1714191, EPI_ISL_1714196, EPI_ISL_1714202, EPI_ISL_1714203, EPI_ISL_1714207, EPI_ISL_1714210, EPI_ISL_1714212, EPI_ISL_1714219, EPI_ISL_1714222, EPI_ISL_1714226, EPI_ISL_1714228, EPI_ISL_1714229, EPI_ISL_1714231, EPI_ISL_1714232, EPI_ISL_1714237, EPI_ISL_1714241, EPI_ISL_1714242, EPI_ISL_1714248, EPI_ISL_1714256, EPI_ISL_1714262, EPI_ISL_1714264, EPI_ISL_1714268, EPI_ISL_1714270, EPI_ISL_1714365, EPI_ISL_1714366, EPI_ISL_1714367, EPI_ISL_1714369, EPI_ISL_1714370, EPI_ISL_1714371, EPI_ISL_1714372, EPI_ISL_1714373, EPI_ISL_1714374, EPI_ISL_1714375, EPI_ISL_1714376, EPI_ISL_1714377, EPI_ISL_1714379, EPI_ISL_1714380, EPI_ISL_1714381, EPI_ISL_1714382, EPI_ISL_1714383, EPI_ISL_1714384, EPI_ISL_1714385, EPI_ISL_1714386, EPI_ISL_1714387, EPI_ISL_1714388, EPI_ISL_1714389, EPI_ISL_1714390, EPI_ISL_1714391, EPI_ISL_1714392, EPI_ISL_1714394, EPI_ISL_1714395, EPI_ISL_1714396, EPI_ISL_1714397, EPI_ISL_1714398, EPI_ISL_1714399, EPI_ISL_1714400, EPI_ISL_1714401, EPI_ISL_1714402, EPI_ISL_1714403, EPI_ISL_1714404, EPI_ISL_1714405, EPI_ISL_1714406, EPI_ISL_1714407, EPI_ISL_1714408, EPI_ISL_1714409, EPI_ISL_1714410, EPI_ISL_1714411, EPI_ISL_1714414, EPI_ISL_1714415, EPI_ISL_1714416, EPI_ISL_1714417, EPI_ISL_1714419, EPI_ISL_1714420, EPI_ISL_1714421, EPI_ISL_1714422, EPI_ISL_1714423, EPI_ISL_1714424, EPI_ISL_1714426, EPI_ISL_1714427, EPI_ISL_1714428, EPI_ISL_1714429, EPI_ISL_1714430, EPI_ISL_1714431, EPI_ISL_1714432, EPI_ISL_1714433, EPI_ISL_1714434, EPI_ISL_1714435, EPI_ISL_1714436, EPI_ISL_1714437, EPI_ISL_1714438, EPI_ISL_1714439, EPI_ISL_1714440, EPI_ISL_1714441, EPI_ISL_1714442, EPI_ISL_1714443, EPI_ISL_1714444, EPI_ISL_1714445, EPI_ISL_1714446, EPI_ISL_1714447, EPI_ISL_1714448, EPI_ISL_1714449, EPI_ISL_1714450, EPI_ISL_1714451, EPI_ISL_1714452, EPI_ISL_1714453, EPI_ISL_1714454, EPI_ISL_1714455, EPI_ISL_1714456, EPI_ISL_1714457, EPI_ISL_1714458, EPI_ISL_1714459, EPI_ISL_1714462, EPI_ISL_1714463, EPI_ISL_1714464, EPI_ISL_1714465, EPI_ISL_1714466, EPI_ISL_1714467, EPI_ISL_1714468, EPI_ISL_1714469, EPI_ISL_1714470, EPI_ISL_1714471, EPI_ISL_1714472, EPI_ISL_1714473, EPI_ISL_1714474, EPI_ISL_1714475, EPI_ISL_1714476, EPI_ISL_1714477, EPI_ISL_1714478, EPI_ISL_1714479, EPI_ISL_1714480, EPI_ISL_1714481, EPI_ISL_1714482, EPI_ISL_1714483, EPI_ISL_1714484, EPI_ISL_1714485, EPI_ISL_1714486, EPI_ISL_1714487, EPI_ISL_1714488, EPI_ISL_1714489, EPI_ISL_1714490, EPI_ISL_1714491, EPI_ISL_1714493, EPI_ISL_1714494, EPI_ISL_1714495, EPI_ISL_1714496, EPI_ISL_1714497, EPI_ISL_1714498, EPI_ISL_1714499, EPI_ISL_1714500, EPI_ISL_1714501, EPI_ISL_1714502, EPI_ISL_1714503, EPI_ISL_1714504, EPI_ISL_1714505, EPI_ISL_1714506, EPI_ISL_1714507, EPI_ISL_1714508, EPI_ISL_1714509, EPI_ISL_1714510, EPI_ISL_1714512, EPI_ISL_1714513, EPI_ISL_1714514, EPI_ISL_1714515, EPI_ISL_1714516, EPI_ISL_1714517, EPI_ISL_1714518, EPI_ISL_1714519, EPI_ISL_1714520, EPI_ISL_1714521, EPI_ISL_1714522, EPI_ISL_1714523, EPI_ISL_1714525, EPI_ISL_1714526, EPI_ISL_1714527, EPI_ISL_1714528, EPI_ISL_1714529, EPI_ISL_1714530, EPI_ISL_1714531, EPI_ISL_1714532, EPI_ISL_1714533, EPI_ISL_1714534, EPI_ISL_1714535, EPI_ISL_1714536, EPI_ISL_1714537, EPI_ISL_1714539, EPI_ISL_1714541, EPI_ISL_1714542, EPI_ISL_1714543, EPI_ISL_1714544, EPI_ISL_1714545, EPI_ISL_1714546, EPI_ISL_1714548, EPI_ISL_1714549			
see above	Ministry of Public Health / Hamad Medical Corporation	Weill Cornell Medical College - Qatar (WCM-Q), Genomics Core Laboratory / Qatar Genome Project (QGP)	Chadi Saad MOPH and HMC: Abdullatif Al-Khal; Dina Elgakhlab; Einas A. E. Al-Kuwari; Hamad E. Al-Romaihi; Hamda Alromaihi; Joel A Malek. QGP: Fatima H. Al-Kuwari; Laith Abu-Raddad; Mashael A. Al-Bader; Meryem Bensaad; Mohammed Al-Thani; Muna A. S. Al-Maslamani; Peter V. Coyle; Reham A. El-Kahlout. QBB: Tasneem Al-Hamad; Roberto Bertollini; Salih Al-Marri; Shameem Younuskunju; WCMQ: Ayeda A. Ahmed; Yasmin Mohamoud
EPI_ISL_486888, EPI_ISL_486889	National Influenza Center, Bahrain	National Influenza Center, Bahrain	AlWasti, H.; Altaif, Z.; Shehab, F.; Zaed, A.
EPI_ISL_1795048	Oman-NIC	Department of Microbiology and Immunology-SQUH	Abdulla Balkhair; Ahlam Al-Amri; Aisha Al-Amri; Aisha Al-Busaidi; Amina Al Jardani; Fahad Zadjali; Fatma BaAlawi; Hamida AL Barwani; Hanan Al-kindi; Intisar Al-Shukri; Khulood Al-Mammary; Mohammed Al-Tobi; Samiha Al Kharusi; Samira Al-Maruqi; Zeyana AL-Dahmani
EPI_ISL_1795049, EPI_ISL_1795050, EPI_ISL_1795051, EPI_ISL_1795052	Oman-NIC	Oman-National Influenza Center-Department of Microbiology and Immunology-SQUH	Abdulla Balkhair; Ahlam Al-Amri; Aisha Al-Amri; Aisha Al-Busaidi; Amina Al Jardani; Fahad Zadjali; Fatma BaAlawi; Hamida AL Barwani; Hanan Al-kindi; Intisar Al-Shukri; Khulood Al-Mammary; Mohammed Al-Tobi; Samiha Al Kharusi; Samira Al-Maruqi; Zeyana AL-Dahmani
EPI_ISL_1819072, EPI_ISL_1819075, EPI_ISL_1819076	Oman-NIC	Oman-National Influenza Center-Department of Microbiology and Immunology-SQUH	Abdulla Balkhair; Ahlam Al-Amri; Aisha Al-Amri; Aisha Al-Busaidi; Amina Al Jardani; Fahad Zadjali; Fatma BaAlawi; Hamida AL Barwani; Hanan Al-kindi; Intisar Al-Shukri; Khulood Al-Mammary; Mohammed Al-Tobi; Samiha Al Kharusi; Samira Al-Maruqi; Zeyana AL-Dahmani
EPI_ISL_1532283, EPI_ISL_1532284, EPI_ISL_1532285, EPI_ISL_1532286, EPI_ISL_1532287, EPI_ISL_1532288, EPI_ISL_1532289, EPI_ISL_1532302, EPI_ISL_1532303, EPI_ISL_1532305, EPI_ISL_2603816, EPI_ISL_2603841, EPI_ISL_2603855, EPI_ISL_2692996, EPI_ISL_2692997			
see above	Oman-National Influenza Center	Biotechnology & OMICs Laboratory	; Abdul Latif Khan; Ahmed Al Harrasi; Ahmed Al Harrasi.; Ahmed Al-Rawahi; Ahmed N Al-Rawahi; Aisha Al-Amri; Amal Al-Maani; Amina Al-Jardani; Amina Al-Jardani.; Bilal Hussain; Fahad Al-Balushi; Hanan Al-Kindi; Intisar Al-Shukri; Laila Al-Balushi; Sajjad Asaf; Samira Al-Mahruqi; Samiya Al-Zadjali; Saqib Bilal
EPI_ISL_678247	Pathogen Genomics Lab King Abdullah University of Science and Technology (KAUST)	Pathogen Genomics Lab King Abdullah University of Science and Technology (KAUST)	Abbas Al Mutair; Arnab Pain; Awad Al-Omari; Muhammad Shuaib; Olga Douvropoulou; Raeece Naeem; Raushan Nugmanova; Samer Salih; Sara Mfarrej; Sharif Hala
EPI_ISL_512874, EPI_ISL_512875, EPI_ISL_512876, EPI_ISL_512877, EPI_ISL_512882, EPI_ISL_512883, EPI_ISL_512895, EPI_ISL_512899, EPI_ISL_512900, EPI_ISL_512901, EPI_ISL_512902, EPI_ISL_512904, EPI_ISL_512906, EPI_ISL_512907, EPI_ISL_512920, EPI_ISL_512922, EPI_ISL_512923, EPI_ISL_512924, EPI_ISL_512927, EPI_ISL_513064, EPI_ISL_513065, EPI_ISL_513071, EPI_ISL_513072, EPI_ISL_677921, EPI_ISL_677922, EPI_ISL_677923, EPI_ISL_677926, EPI_ISL_677927, EPI_ISL_677928, EPI_ISL_677929, EPI_ISL_677930, EPI_ISL_677931, EPI_ISL_677932, EPI_ISL_677933, EPI_ISL_677934, EPI_ISL_677935, EPI_ISL_677936, EPI_ISL_677991, EPI_ISL_677992, EPI_ISL_677993, EPI_ISL_677994, EPI_ISL_677995, EPI_ISL_677996, EPI_ISL_677997, EPI_ISL_678001, EPI_ISL_678006, EPI_ISL_678007, EPI_ISL_678008, EPI_ISL_678009, EPI_ISL_678064, EPI_ISL_678070, EPI_ISL_678083, EPI_ISL_678084, EPI_ISL_678085, EPI_ISL_678086, EPI_ISL_678087, EPI_ISL_678088, EPI_ISL_678089, EPI_ISL_678090, EPI_ISL_678091, EPI_ISL_678092, EPI_ISL_678093, EPI_ISL_678094, EPI_ISL_678095, EPI_ISL_678154, EPI_ISL_678155, EPI_ISL_678157, EPI_ISL_678158, EPI_ISL_678159, EPI_ISL_678184, EPI_ISL_678211, EPI_ISL_678212, EPI_ISL_678213, EPI_ISL_678214, EPI_ISL_678215, EPI_ISL_678216, EPI_ISL_678217, EPI_ISL_678218, EPI_ISL_678219, EPI_ISL_678220, EPI_ISL_678221, EPI_ISL_678222, EPI_ISL_678223, EPI_ISL_678224, EPI_ISL_678225, EPI_ISL_678226, EPI_ISL_678239, EPI_ISL_678241, EPI_ISL_751225, EPI_ISL_751226, EPI_ISL_751227, EPI_ISL_751228, EPI_ISL_751229, EPI_ISL_751230, EPI_ISL_751231, EPI_ISL_751232, EPI_ISL_751233, EPI_ISL_751234, EPI_ISL_751235, EPI_ISL_751236			
see above	Pathogen Genomics Lab King Abdullah University of Science and Technology(KAUST)	Pathogen Genomics Lab King Abdullah University of Science and Technology(KAUST)	Abbas Al Mutair; Abdulaziz Alahmadi; Afrah Alsomali; Ahmad Bakur Mahmoud; Amanda; Amanda Ooi; Amit Kumar Subudhi; Anwar Hashem; Arnab Pain; Asim Khogeer; Awad Al-Omari; Fadwa Alofi; Fathia Ben Rached; Jumana Taha; Kahled Algithami; Luke; Luke Esau; Muhammad Shuaib; Naif Almontashiri; Olga Douvropoulou; Raeece Naeem; Rahul P Salunke; Raushan Nugmanova; Samer Salih; Sara Mfarrej; Sharif Hala
EPI_ISL_636973	Public Health Lab	Public Health Lab	Alwasti; H
EPI_ISL_582642, EPI_ISL_582643, EPI_ISL_582644, EPI_ISL_582645, EPI_ISL_582646	Sheikh Khalifa Medical City	Molecular/Surveillance lab Sheikh Khalifa Medical City	Amirtharaj Francis; Hala Imambaccus; Hiba Saud; Sahar Almarzooqi; Sajeed Abdul; Stefan Weber
EPI_ISL_487270, EPI_ISL_487272, EPI_ISL_487273, EPI_ISL_487274	unknown	Communicable Disease Laboratory, Public Health Directorate	AlTaif, Z.; AlWasti, H.; Altaif, Z.; Altaif, z.; Shehab, F.; Zaed, A.

We gratefully acknowledge the following Authors from the Originating laboratories responsible for obtaining the specimens, as well as the Submitting laboratories where the genome data were generated and shared via GISAID, on which this research is based.

All Submitters of data may be contacted directly via www.gisaid.org

Authors are sorted alphabetically.

Acknowledgement EPI_SET Identifier: EPI_SET_20220314fw

Accession ID	Originating Laboratory	Submitting Laboratory	Authors
EPI_ISL_859576, EPI_ISL_859577, EPI_ISL_859601, EPI_ISL_859602, EPI_ISL_859682, EPI_ISL_859683, EPI_ISL_859684, EPI_ISL_859685, EPI_ISL_859686, EPI_ISL_859687, EPI_ISL_859688, EPI_ISL_859689, EPI_ISL_859690, EPI_ISL_859691, EPI_ISL_859692, EPI_ISL_859693, EPI_ISL_859694, EPI_ISL_859695, EPI_ISL_859697, EPI_ISL_859698, EPI_ISL_859699, EPI_ISL_859703, EPI_ISL_859705, EPI_ISL_859707, EPI_ISL_859708, EPI_ISL_859713, EPI_ISL_859715, EPI_ISL_859719, EPI_ISL_859720, EPI_ISL_859723, EPI_ISL_859727, EPI_ISL_859731, EPI_ISL_859734, EPI_ISL_859739, EPI_ISL_859748, EPI_ISL_859755, EPI_ISL_859756, EPI_ISL_859757, EPI_ISL_859758, EPI_ISL_859760, EPI_ISL_859761, EPI_ISL_859763, EPI_ISL_859764, EPI_ISL_859767, EPI_ISL_859768, EPI_ISL_859770, EPI_ISL_859771, EPI_ISL_859772, EPI_ISL_859773, EPI_ISL_859774, EPI_ISL_859775, EPI_ISL_859776, EPI_ISL_859777, EPI_ISL_859778, EPI_ISL_859780, EPI_ISL_859781, EPI_ISL_859782, EPI_ISL_859783, EPI_ISL_859784, EPI_ISL_859785, EPI_ISL_859786, EPI_ISL_859788, EPI_ISL_859789, EPI_ISL_859790, EPI_ISL_859791, EPI_ISL_859792, EPI_ISL_859793, EPI_ISL_859794, EPI_ISL_859795, EPI_ISL_859796, EPI_ISL_859798, EPI_ISL_859799, EPI_ISL_859800, EPI_ISL_859801, EPI_ISL_859802, EPI_ISL_859803, EPI_ISL_859804, EPI_ISL_859805, EPI_ISL_859808, EPI_ISL_859809, EPI_ISL_859810, EPI_ISL_859811, EPI_ISL_859812, EPI_ISL_859813, EPI_ISL_859814, EPI_ISL_859815, EPI_ISL_859816, EPI_ISL_859817, EPI_ISL_859818, EPI_ISL_859819, EPI_ISL_859820, EPI_ISL_859821, EPI_ISL_859822, EPI_ISL_859823, EPI_ISL_859824, EPI_ISL_859825, EPI_ISL_859826, EPI_ISL_859827, EPI_ISL_859828, EPI_ISL_859829, EPI_ISL_859830, EPI_ISL_859832, EPI_ISL_859833, EPI_ISL_859834, EPI_ISL_859835, EPI_ISL_859836, EPI_ISL_859837, EPI_ISL_859839, EPI_ISL_859841, EPI_ISL_859842, EPI_ISL_859845, EPI_ISL_859846, EPI_ISL_859847, EPI_ISL_859849, EPI_ISL_859850, EPI_ISL_859851, EPI_ISL_859852, EPI_ISL_859853, EPI_ISL_859854, EPI_ISL_859855, EPI_ISL_859856, EPI_ISL_859857, EPI_ISL_859858, EPI_ISL_859859, EPI_ISL_859860, EPI_ISL_859861, EPI_ISL_859862, EPI_ISL_859863, EPI_ISL_859864, EPI_ISL_859865, EPI_ISL_859866, EPI_ISL_859867, EPI_ISL_859868, EPI_ISL_859870, EPI_ISL_859873, EPI_ISL_859874, EPI_ISL_859875, EPI_ISL_859876, EPI_ISL_859877, EPI_ISL_859878, EPI_ISL_859881, EPI_ISL_859882, EPI_ISL_859884, EPI_ISL_859885, EPI_ISL_859886, EPI_ISL_859887, EPI_ISL_859888, EPI_ISL_859889, EPI_ISL_859890, EPI_ISL_859891, EPI_ISL_859893, EPI_ISL_859895, EPI_ISL_859896, EPI_ISL_859897, EPI_ISL_859898, EPI_ISL_859899, EPI_ISL_859901, EPI_ISL_859903, EPI_ISL_859904, EPI_ISL_859905, EPI_ISL_859907, EPI_ISL_859908, EPI_ISL_859909, EPI_ISL_859910, EPI_ISL_859911, EPI_ISL_859912, EPI_ISL_859913, EPI_ISL_859914, EPI_ISL_859915, EPI_ISL_859916, EPI_ISL_859918, EPI_ISL_859919, EPI_ISL_859920, EPI_ISL_859922, EPI_ISL_859923, EPI_ISL_859924, EPI_ISL_859925, EPI_ISL_859926, EPI_ISL_859927, EPI_ISL_859929, EPI_ISL_859930, EPI_ISL_859931, EPI_ISL_859932, EPI_ISL_859933, EPI_ISL_860026, EPI_ISL_860027, EPI_ISL_860028, EPI_ISL_860029, EPI_ISL_860030, EPI_ISL_860031, EPI_ISL_860032, EPI_ISL_860033, EPI_ISL_860034, EPI_ISL_860035, EPI_ISL_860036, EPI_ISL_860039, EPI_ISL_860040, EPI_ISL_860042, EPI_ISL_860043, EPI_ISL_860044, EPI_ISL_860046, EPI_ISL_860047, EPI_ISL_860048, EPI_ISL_860049, EPI_ISL_860051, EPI_ISL_860052, EPI_ISL_860053, EPI_ISL_860054, EPI_ISL_860055, EPI_ISL_860056, EPI_ISL_860058, EPI_ISL_860059, EPI_ISL_860060, EPI_ISL_860061, EPI_ISL_860062, EPI_ISL_860063, EPI_ISL_860064, EPI_ISL_860065, EPI_ISL_860066, EPI_ISL_860067, EPI_ISL_860068, EPI_ISL_860069, EPI_ISL_860070, EPI_ISL_860071, EPI_ISL_860072, EPI_ISL_860073, EPI_ISL_860074, EPI_ISL_860075, EPI_ISL_860076, EPI_ISL_860077, EPI_ISL_860079, EPI_ISL_860080, EPI_ISL_860083, EPI_ISL_860084, EPI_ISL_860085, EPI_ISL_860086, EPI_ISL_860088, EPI_ISL_860089, EPI_ISL_860090, EPI_ISL_860091			
see above	BTC, Khalifa University	BTC, Khalifa University	Al Safar et al
EPI_ISL_632250, EPI_ISL_632251, EPI_ISL_632252, EPI_ISL_632253, EPI_ISL_632254, EPI_ISL_632255, EPI_ISL_632256, EPI_ISL_632257, EPI_ISL_632258, EPI_ISL_632259, EPI_ISL_632260, EPI_ISL_632264, EPI_ISL_632268, EPI_ISL_632269, EPI_ISL_632270, EPI_ISL_632271, EPI_ISL_632272, EPI_ISL_632273, EPI_ISL_632274, EPI_ISL_632275, EPI_ISL_632277, EPI_ISL_632278, EPI_ISL_632279, EPI_ISL_632281, EPI_ISL_632282, EPI_ISL_632899, EPI_ISL_632900, EPI_ISL_632901, EPI_ISL_632902, EPI_ISL_632903, EPI_ISL_632904, EPI_ISL_632905, EPI_ISL_632906, EPI_ISL_632907, EPI_ISL_678261, EPI_ISL_678262, EPI_ISL_678264, EPI_ISL_678265, EPI_ISL_678266, EPI_ISL_678267, EPI_ISL_678269, EPI_ISL_678270, EPI_ISL_678271, EPI_ISL_678272, EPI_ISL_681298, EPI_ISL_681299, EPI_ISL_681300, EPI_ISL_681301, EPI_ISL_681302, EPI_ISL_681303, EPI_ISL_681304, EPI_ISL_681305, EPI_ISL_681306, EPI_ISL_681307, EPI_ISL_681308, EPI_ISL_681309, EPI_ISL_681310, EPI_ISL_681311, EPI_ISL_681312, EPI_ISL_681313, EPI_ISL_681314, EPI_ISL_681315, EPI_ISL_681316, EPI_ISL_681317, EPI_ISL_681318, EPI_ISL_681319, EPI_ISL_682299, EPI_ISL_682300, EPI_ISL_682301, EPI_ISL_682302, EPI_ISL_682303, EPI_ISL_682304, EPI_ISL_682305, EPI_ISL_682306, EPI_ISL_682307, EPI_ISL_682308, EPI_ISL_682309, EPI_ISL_682310, EPI_ISL_682311, EPI_ISL_682312, EPI_ISL_682313, EPI_ISL_682314, EPI_ISL_682315, EPI_ISL_682316, EPI_ISL_682317, EPI_ISL_682318, EPI_ISL_682319, EPI_ISL_682320, EPI_ISL_682321, EPI_ISL_682322, EPI_ISL_684028, EPI_ISL_684029, EPI_ISL_684030, EPI_ISL_684031, EPI_ISL_684032, EPI_ISL_684033, EPI_ISL_684034, EPI_ISL_684035, EPI_ISL_684036			
see above	Communicable Disease Laboratory, Public Health Directorate	Communicable Disease Laboratory, Public Health Directorate	AlAbbas, Z.; AlHujairi, Z.; AlTaif, Z.; AlWasti, H.; Alhujairi, Z.; Altaif, Z.; Alwasti, H.
EPI_ISL_903381, EPI_ISL_903382, EPI_ISL_903383, EPI_ISL_903388, EPI_ISL_903389	MOH - Jaber Al-Ahmad Hospital (Innovation Research Laboratory)	MOH - Jaber Al-Ahmad Hospital (Innovation Research Laboratory)	Mohammad Alghounaim; Salman Al-Sabah
EPI_ISL_1713196, EPI_ISL_1713197, EPI_ISL_1713198, EPI_ISL_1713199, EPI_ISL_1713201, EPI_ISL_1713202, EPI_ISL_1713203, EPI_ISL_1713204, EPI_ISL_1713205, EPI_ISL_1713208, EPI_ISL_1713209, EPI_ISL_1713210, EPI_ISL_1713211, EPI_ISL_1713212, EPI_ISL_1713213, EPI_ISL_1713214, EPI_ISL_1713216, EPI_ISL_1713219, EPI_ISL_1713220, EPI_ISL_1713222, EPI_ISL_1713225, EPI_ISL_1713226, EPI_ISL_1713227, EPI_ISL_1713229, EPI_ISL_1713231, EPI_ISL_1713232, EPI_ISL_1713236, EPI_ISL_1713237, EPI_ISL_1713238, EPI_ISL_1713239, EPI_ISL_1713248, EPI_ISL_1713250, EPI_ISL_1713254, EPI_ISL_1713255, EPI_ISL_1713259, EPI_ISL_1713266, EPI_ISL_1713268, EPI_ISL_1713275, EPI_ISL_1713276, EPI_ISL_1713278, EPI_ISL_1713279, EPI_ISL_1713281, EPI_ISL_1713283, EPI_ISL_1713422, EPI_ISL_1713425, EPI_ISL_1713426, EPI_ISL_1713429, EPI_ISL_1713870, EPI_ISL_1713895, EPI_ISL_1713896			
see above	Ministry of Public Health / Hamad Medical Corporation	Biomedical Research Center (BRC), Qatar University / Qatar Genome Project (QGP)	Asmaa A. Al-Thani. MOPH and HMC: Abdullatif Al-Khal; BRC: Fatiha M. Benslimane; Chadi Saad; Dana Al-Batesh; Dina Elgakhlab QGP: Fatima H. Al-Kuwari; Einas A. E. Al-Kuwari; Hadi M. Yassine; Hamad E. Al-Romaihi; Hamda Alromaihi; Heba A. Al-Khatib; Mashael A. Al-Bader; Mohammed Al-Thani; Muna A. S. Al-Maslamani; Oal Al-Jamal; Peter V. Coyle; Reham A. El-Kahlout. QBB: Tasneem Al-Hamad; Roberto Bertollini; Salih Al-Marri
EPI_ISL_1714179, EPI_ISL_1714183, EPI_ISL_1714276, EPI_ISL_1714277, EPI_ISL_1714278, EPI_ISL_1714282, EPI_ISL_1714283, EPI_ISL_1714284, EPI_ISL_1714288, EPI_ISL_1714289, EPI_ISL_1714290, EPI_ISL_1714291, EPI_ISL_1714292, EPI_ISL_1714293, EPI_ISL_1714294, EPI_ISL_1714295, EPI_ISL_1714296, EPI_ISL_1714303, EPI_ISL_1714304, EPI_ISL_1714305, EPI_ISL_1714306, EPI_ISL_1714307, EPI_ISL_1714308, EPI_ISL_1714309, EPI_ISL_1714311, EPI_ISL_1714312, EPI_ISL_1714313, EPI_ISL_1714315, EPI_ISL_1714316, EPI_ISL_1714317, EPI_ISL_1714318, EPI_ISL_1714319, EPI_ISL_1714320, EPI_ISL_1714321, EPI_ISL_1714323, EPI_ISL_1714324, EPI_ISL_1714325, EPI_ISL_1714326, EPI_ISL_1714327, EPI_ISL_1714328, EPI_ISL_1714329, EPI_ISL_1714331, EPI_ISL_1714332, EPI_ISL_1714333, EPI_ISL_1714334, EPI_ISL_1714336, EPI_ISL_1714338, EPI_ISL_1714339, EPI_ISL_1714340, EPI_ISL_1714341, EPI_ISL_1714343, EPI_ISL_1714346, EPI_ISL_1714348, EPI_ISL_1714350			
see above	Ministry of Public Health / Hamad Medical Corporation	Weill Cornell Medical College - Qatar (WCM-Q), Genomics Core Laboratory / Qatar Genome Project (QGP)	Chadi Saad MOPH and HMC: Abdullatif Al-Khal; Dina Elgakhlab; Einas A. E. Al-Kuwari; Hamad E. Al-Romaihi; Hamda Alromaihi; Joel A Malek. QGP: Fatima H. Al-Kuwari; Laith Abu-Raddad; Mashael A. Al-Bader; Meryem Bensaad; Mohammed Al-Thani; Muna A. S. Al-Maslamani; Peter V. Coyle; Reham A. El-Kahlout. QBB: Tasneem Al-Hamad; Roberto Bertollini; Salih Al-Marri; Shameem Younuskunju; WCMQ: Ayeda A. Ahmed; Yasmin Mohamoud
EPI_ISL_2429123, EPI_ISL_2429124, EPI_ISL_2429125, EPI_ISL_2429126, EPI_ISL_2429127	Mubarak Al-Kabeer Hospital	Virology Unit, Department of Microbiology, Faculty of Medicine, Kuwait University	Anfal Al-Adwani; Hussain Safar; Nada Madi
EPI_ISL_2429129	Mubaral Al-Kabeer Hospital	Virology Unit, Department of Microbiology, Faculty of Medicine, Kuwait University	Anfal Al-Adwani; Hussain Safar; Nada Madi
EPI_ISL_1517192, EPI_ISL_1532290, EPI_ISL_1532291, EPI_ISL_1532292, EPI_ISL_1532293, EPI_ISL_1532294, EPI_ISL_1532295, EPI_ISL_1532296, EPI_ISL_1532297, EPI_ISL_1532298, EPI_ISL_1532300, EPI_ISL_1532301, EPI_ISL_1532304			
see above	Oman-National Influenza Center	Biotechnology & OMICs Laboratory	Abdul Latif Khan; Ahmed Al Harrasi; Ahmed Al Harrasi.; Ahmed Al-Rawahi; Ahmed N Al-Rawahi; Aisha Al-Amri; Amal Al-Maani; Amina Al-Jardani; Amina Al-Jardani.; Bilal Hussain; Hanan Al-Kindi; Intisar Al-Shukri; Sajjad Asaf; Samira Al-Mahruqi; Samiya Al-Zadjali; Saqib Bilal
EPI_ISL_766569	Oman-National Influenza Center	Oman-National Influenza Center	Aisha Al-Busaidi; Amina Al-Jardani; Hamida Al-Barwani; Hanan Al-Kindi; Intisar Al-Shukri; Laila Al-Balushi; Samiha Al-Kharusi; Samira Al-Mahruqi
EPI_ISL_582648, EPI_ISL_582649, EPI_ISL_582650, EPI_ISL_582651, EPI_ISL_582653, EPI_ISL_582655, EPI_ISL_582656, EPI_ISL_582657, EPI_ISL_582659, EPI_ISL_582662, EPI_ISL_582663, EPI_ISL_582665, EPI_ISL_582667, EPI_ISL_582672, EPI_ISL_582673, EPI_ISL_582674, EPI_ISL_582675, EPI_ISL_582676, EPI_ISL_582677, EPI_ISL_582678, EPI_ISL_582679, EPI_ISL_582680, EPI_ISL_582681, EPI_ISL_582682, EPI_ISL_582683, EPI_ISL_582684, EPI_ISL_582685, EPI_ISL_582686, EPI_ISL_582687, EPI_ISL_582688, EPI_ISL_582689, EPI_ISL_582690			
see above	Sheikh Khalifa Medical City	Molecular/Surveillance lab Sheikh Khalifa Medical City	Amirtharaj Francis; Hala Imambaccus; Hiba Saud; Sahar Almarzooqi; Sajeed Abdul; Stefan Weber

We gratefully acknowledge the following Authors from the Originating laboratories responsible for obtaining the specimens, as well as the Submitting laboratories where the genome data were generated and shared via GISAID, on which this research is based.

All Submitters of data may be contacted directly via www.gisaid.org

Authors are sorted alphabetically.

Acknowledgement EPI_SET Identifier: EPI_SET_20220314sb

Accession ID	Originating Laboratory	Submitting Laboratory	Authors
EPI_ISL_2001099	BIOSCIENCE, King Faisal Hospital Research Center	BIOSCIENCE, King Faisal Hospital Research Center	Al-Qahtani A, A.; Alahideb B, M.; Alhamlan F, S.; Almaghrabi R, S.; Alsanea M, S.; Althawadi S, I.; K. and Balavenkatesh Mani, M.; Mutabagani M, S.; UdayaRaja G
EPI_ISL_1660421, EPI_ISL_1660422, EPI_ISL_1660423, EPI_ISL_1660424, EPI_ISL_1660425, EPI_ISL_1660426, EPI_ISL_1660428, EPI_ISL_1660430, EPI_ISL_1660431, EPI_ISL_1660433, EPI_ISL_1660437, EPI_ISL_1660438, EPI_ISL_1660439, EPI_ISL_1660440, EPI_ISL_1660442, EPI_ISL_1660444, EPI_ISL_1660445, EPI_ISL_1660446, EPI_ISL_1660447, EPI_ISL_1660449, EPI_ISL_1660451, EPI_ISL_1660454, EPI_ISL_1660457, EPI_ISL_1660458, EPI_ISL_1660459, EPI_ISL_1660460, EPI_ISL_1660461, EPI_ISL_1660464, EPI_ISL_1660465, EPI_ISL_1660466, EPI_ISL_1660468, EPI_ISL_1660469, EPI_ISL_1660470, EPI_ISL_1660471, EPI_ISL_2715285, EPI_ISL_2715286, EPI_ISL_2715287, EPI_ISL_2715288, EPI_ISL_2715289, EPI_ISL_2812539, EPI_ISL_2812540, EPI_ISL_2812541, EPI_ISL_2812542, EPI_ISL_2812543, EPI_ISL_2812547, EPI_ISL_2812558, EPI_ISL_2812560, EPI_ISL_2812562, EPI_ISL_2812573, EPI_ISL_2812574			
see above	Communicable Disease Laboratory, Public Health Directorate	Communicable Disease Laboratory, Public Health Directorate	AlAbbas, Z.; AlHujairi, Z.; Almoamen, G.; Alwasti, H.; Marhoon, A.
EPI_ISL_2107507, EPI_ISL_2151336, EPI_ISL_2151337	Infectious Diseases, King Faisal Hospital Research Center	Infectious Diseases, King Faisal Hospital Research Center	Al-Qahtani A, A.; Alahideb B, M.; Alhamlan F, S.; Almaghrabi R, S.; Alsanea M; Alsanea M, S.; Althawadi S, I.; Balavenkatesh Mani; Balavenkatesh Mani, M.; M. and UdayaRaja G, K.; Mutabagani M, S.; S. and Alahideb B, M.; UdayaRaja G, K.
EPI_ISL_2673521	MOH - Jaber Al-Ahmad Hospital (Innovation Research Laboratory)	MOH - Jaber Al-Ahmad Hospital (Innovation Research Laboratory)	Mohammad Alghounaim; Salman Al-Sabah
EPI_ISL_1713520, EPI_ISL_1713524, EPI_ISL_1713528, EPI_ISL_1713569, EPI_ISL_1713584, EPI_ISL_1713585, EPI_ISL_1713588, EPI_ISL_1713593, EPI_ISL_1713595, EPI_ISL_1713599, EPI_ISL_1713601, EPI_ISL_1713603, EPI_ISL_1713605, EPI_ISL_1713609, EPI_ISL_1713613, EPI_ISL_1713615, EPI_ISL_1713617, EPI_ISL_1713618, EPI_ISL_1713621, EPI_ISL_1713622, EPI_ISL_1713623, EPI_ISL_1713628, EPI_ISL_1713629, EPI_ISL_1713630, EPI_ISL_1713638, EPI_ISL_1713642, EPI_ISL_1713643, EPI_ISL_1713644, EPI_ISL_1713651, EPI_ISL_1713653, EPI_ISL_1713668, EPI_ISL_1713669, EPI_ISL_1713671, EPI_ISL_1713676, EPI_ISL_1713679, EPI_ISL_1713683, EPI_ISL_1713687, EPI_ISL_1713689, EPI_ISL_1713691, EPI_ISL_1713692, EPI_ISL_1713694, EPI_ISL_1713695, EPI_ISL_1713697, EPI_ISL_1713700, EPI_ISL_1713701, EPI_ISL_1713704, EPI_ISL_1713705, EPI_ISL_1713706, EPI_ISL_1713707, EPI_ISL_1713708, EPI_ISL_1713710, EPI_ISL_1713711, EPI_ISL_1713714, EPI_ISL_1713717, EPI_ISL_1713720, EPI_ISL_1713723, EPI_ISL_1713736, EPI_ISL_1713746, EPI_ISL_1713750, EPI_ISL_1713751, EPI_ISL_1713752, EPI_ISL_1713753, EPI_ISL_1713754, EPI_ISL_1713755, EPI_ISL_1713760, EPI_ISL_1713769, EPI_ISL_1713772, EPI_ISL_1713776, EPI_ISL_1713783, EPI_ISL_1713787, EPI_ISL_1713812, EPI_ISL_1713876, EPI_ISL_1713892, EPI_ISL_1713894, EPI_ISL_1713902, EPI_ISL_1713908, EPI_ISL_1713911, EPI_ISL_1713915, EPI_ISL_1713924, EPI_ISL_1713936, EPI_ISL_1713937, EPI_ISL_1713945, EPI_ISL_1713946, EPI_ISL_2274300, EPI_ISL_2274305, EPI_ISL_2274309, EPI_ISL_2274311, EPI_ISL_2274317, EPI_ISL_2274326, EPI_ISL_2274334, EPI_ISL_2274338, EPI_ISL_2274349, EPI_ISL_2274360, EPI_ISL_2274404, EPI_ISL_2274421, EPI_ISL_2274424, EPI_ISL_2274429, EPI_ISL_2274432, EPI_ISL_2274435, EPI_ISL_2408317, EPI_ISL_2408352, EPI_ISL_2842847, EPI_ISL_2842848, EPI_ISL_2842849, EPI_ISL_2842850, EPI_ISL_2842851, EPI_ISL_2842852, EPI_ISL_2842853, EPI_ISL_2842854, EPI_ISL_2842855, EPI_ISL_2842856, EPI_ISL_2842857, EPI_ISL_2842858, EPI_ISL_2842859, EPI_ISL_2842860, EPI_ISL_2842861, EPI_ISL_2842862, EPI_ISL_2842863, EPI_ISL_2842864, EPI_ISL_2842865, EPI_ISL_2842866, EPI_ISL_2842867, EPI_ISL_2842868, EPI_ISL_2842869, EPI_ISL_2842870, EPI_ISL_2842871, EPI_ISL_2842872, EPI_ISL_2842873, EPI_ISL_2842874, EPI_ISL_2842875, EPI_ISL_2842876, EPI_ISL_2842877, EPI_ISL_2842878, EPI_ISL_2842879, EPI_ISL_2842880, EPI_ISL_2842881, EPI_ISL_2842882, EPI_ISL_2842883, EPI_ISL_2842884, EPI_ISL_2842885, EPI_ISL_2842886, EPI_ISL_2842887, EPI_ISL_2842888, EPI_ISL_2842889, EPI_ISL_2842890, EPI_ISL_2842891, EPI_ISL_2842892, EPI_ISL_2842893, EPI_ISL_2842894, EPI_ISL_2842895, EPI_ISL_2842896, EPI_ISL_2842897, EPI_ISL_2842898, EPI_ISL_2842899, EPI_ISL_2842900, EPI_ISL_2842901, EPI_ISL_2842902, EPI_ISL_2842903, EPI_ISL_2842904, EPI_ISL_2842905, EPI_ISL_2842906, EPI_ISL_2842907, EPI_ISL_2842908, EPI_ISL_2842909, EPI_ISL_2842910, EPI_ISL_2842911, EPI_ISL_2842912, EPI_ISL_2842913, EPI_ISL_2842914, EPI_ISL_2842915, EPI_ISL_2842916, EPI_ISL_2842917, EPI_ISL_2842918, EPI_ISL_2842919, EPI_ISL_2842920, EPI_ISL_2842921, EPI_ISL_2842922, EPI_ISL_2842923, EPI_ISL_2842924, EPI_ISL_2842925, EPI_ISL_2842926, EPI_ISL_2842927, EPI_ISL_2842928, EPI_ISL_2842929, EPI_ISL_2842930, EPI_ISL_2842931, EPI_ISL_2842932, EPI_ISL_2842933, EPI_ISL_2842934, EPI_ISL_2842935, EPI_ISL_2842936, EPI_ISL_2842937, EPI_ISL_2842938, EPI_ISL_2842939, EPI_ISL_2842940, EPI_ISL_2842941, EPI_ISL_2842942, EPI_ISL_2842943, EPI_ISL_2842944, EPI_ISL_2842945, EPI_ISL_2842946, EPI_ISL_2842947, EPI_ISL_2842948, EPI_ISL_2842949, EPI_ISL_2842950, EPI_ISL_2842951, EPI_ISL_2842952, EPI_ISL_2842953, EPI_ISL_2842954, EPI_ISL_2842955, EPI_ISL_2842956, EPI_ISL_2842957, EPI_ISL_2842958, EPI_ISL_2842959, EPI_ISL_2842960, EPI_ISL_2842961, EPI_ISL_2842962, EPI_ISL_2842963, EPI_ISL_2842964, EPI_ISL_2842965, EPI_ISL_2842966, EPI_ISL_2842967, EPI_ISL_2842968, EPI_ISL_2842969, EPI_ISL_2842970, EPI_ISL_2842971, EPI_ISL_2842972, EPI_ISL_2842973, EPI_ISL_2842974, EPI_ISL_2842975, EPI_ISL_2842976, EPI_ISL_2842977, EPI_ISL_2842978, EPI_ISL_2842979, EPI_ISL_2842980, EPI_ISL_2842981, EPI_ISL_2842982, EPI_ISL_2842983, EPI_ISL_2842984, EPI_ISL_2842985, EPI_ISL_2842986, EPI_ISL_2842987, EPI_ISL_2842988, EPI_ISL_2842989, EPI_ISL_2842990, EPI_ISL_2842991, EPI_ISL_2842992, EPI_ISL_2842993, EPI_ISL_2842994, EPI_ISL_2842995, EPI_ISL_2842996, EPI_ISL_2842997, EPI_ISL_2842998, EPI_ISL_2842999, EPI_ISL_2843000, EPI_ISL_2843001, EPI_ISL_2843002, EPI_ISL_2843003, EPI_ISL_2843004, EPI_ISL_2843005, EPI_ISL_2843006, EPI_ISL_2843007, EPI_ISL_2843008, EPI_ISL_2843009, EPI_ISL_2843010, EPI_ISL_2843011, EPI_ISL_2843012, EPI_ISL_2843015, EPI_ISL_2843016, EPI_ISL_2843017, EPI_ISL_2843018, EPI_ISL_2843019, EPI_ISL_2843020, EPI_ISL_2843029, EPI_ISL_2843040, EPI_ISL_2843048, EPI_ISL_2843049, EPI_ISL_2843050, EPI_ISL_2843051, EPI_ISL_2843052, EPI_ISL_2843053, EPI_ISL_2843054, EPI_ISL_2843055, EPI_ISL_2843056, EPI_ISL_2843057, EPI_ISL_2843058, EPI_ISL_2843059, EPI_ISL_2843060, EPI_ISL_2843061, EPI_ISL_2843062, EPI_ISL_2843063, EPI_ISL_2843064, EPI_ISL_2843065, EPI_ISL_2843066, EPI_ISL_2843067, EPI_ISL_2843068, EPI_ISL_2843069, EPI_ISL_2843070, EPI_ISL_2843071, EPI_ISL_2843072, EPI_ISL_2843073, EPI_ISL_2843074, EPI_ISL_2843075, EPI_ISL_2843076, EPI_ISL_2843077, EPI_ISL_2843078, EPI_ISL_2843079, EPI_ISL_2843080, EPI_ISL_2843081, EPI_ISL_2843082, EPI_ISL_2843083, EPI_ISL_2843084, EPI_ISL_2843085, EPI_ISL_2843086, EPI_ISL_2843087, EPI_ISL_2843088, EPI_ISL_2843089, EPI_ISL_2843091, EPI_ISL_2843092, EPI_ISL_2843093, EPI_ISL_2843094, EPI_ISL_2843095, EPI_ISL_2843096, EPI_ISL_2843097, EPI_ISL_2843098, EPI_ISL_2843099, EPI_ISL_2843100, EPI_ISL_2843101, EPI_ISL_2843102, EPI_ISL_2843103, EPI_ISL_2843104, EPI_ISL_2843105, EPI_ISL_2843106, EPI_ISL_2843107, EPI_ISL_2843108, EPI_ISL_2843109, EPI_ISL_2843110, EPI_ISL_2843111, EPI_ISL_2843112, EPI_ISL_2843113, EPI_ISL_2843114, EPI_ISL_2843115, EPI_ISL_2843116, EPI_ISL_2843117, EPI_ISL_2843118, EPI_ISL_2843119, EPI_ISL_2843120, EPI_ISL_2843121, EPI_ISL_2843122, EPI_ISL_2843123, EPI_ISL_2843124, EPI_ISL_2843125, EPI_ISL_2843126, EPI_ISL_2843127, EPI_ISL_2843128, EPI_ISL_2843129, EPI_ISL_2843130, EPI_ISL_2843131, EPI_ISL_2843132, EPI_ISL_2843133, EPI_ISL_2843134, EPI_ISL_2843135, EPI_ISL_2843136, EPI_ISL_2843137, EPI_ISL_2843138, EPI_ISL_2843139, EPI_ISL_2843140, EPI_ISL_2843141, EPI_ISL_2843142, EPI_ISL_2843143, EPI_ISL_2843144, EPI_ISL_2843145, EPI_ISL_2843146, EPI_ISL_2843147, EPI_ISL_2843148, EPI_ISL_2843149, EPI_ISL_2843150, EPI_ISL_2843151, EPI_ISL_2843152, EPI_ISL_2843153, EPI_ISL_2843154, EPI_ISL_2843155, EPI_ISL_2843156, EPI_ISL_2843157, EPI_ISL_2843158, EPI_ISL_2843159, EPI_ISL_2843160, EPI_ISL_2843161, EPI_ISL_2843162, EPI_ISL_2843163, EPI_ISL_2843164, EPI_ISL_2843165, EPI_ISL_2843166, EPI_ISL_2843167, EPI_ISL_2843168, EPI_ISL_2843169, EPI_ISL_2843171, EPI_ISL_2843172, EPI_ISL_2843173, EPI_ISL_2843174, EPI_ISL_2843175, EPI_ISL_2843176, EPI_ISL_2843177, EPI_ISL_2843178, EPI_ISL_2843179, EPI_ISL_2843180, EPI_ISL_2843181, EPI_ISL_2843182, EPI_ISL_2843183, EPI_ISL_2843184, EPI_ISL_2843185, EPI_ISL_2843186, EPI_ISL_2843188, EPI_ISL_2843189, EPI_ISL_2843190, EPI_ISL_2843191, EPI_ISL_2843192, EPI_ISL_2843193, EPI_ISL_2843194, EPI_ISL_2843195, EPI_ISL_2843196, EPI_ISL_2843197, EPI_ISL_2843200, EPI_ISL_2843201, EPI_ISL_2843202, EPI_ISL_2843203, EPI_ISL_2843204, EPI_ISL_2843205, EPI_ISL_2843206, EPI_ISL_2843291, EPI_ISL_2843292, EPI_ISL_2843293, EPI_ISL_2843294, EPI_ISL_2843295, EPI_ISL_2843296, EPI_ISL_2843297, EPI_ISL_2843298, EPI_ISL_2843299, EPI_ISL_2843300, EPI_ISL_2843301, EPI_ISL_2843302			
see above	Ministry of Public Health / Hamad Medical Corporation	Biomedical Research Center (BRC), Qatar University / Qatar Genome Project (QGP)	Asmaa A. Al-Thani. MOPH and HMC: Abdullatif Al-Khal; Asmaa A. Al-Thani. MOPH and HMC: Abdullatif Al-Khal; BRC: Fatiha M. Benslimane; Chadi Saad; Dana Al-Batesh; Dina Elgakhlab QGP: Fatima H. Al-Kuwari; Dina Elgakhlab QGP: Fatima H. Al-Kuwari; Einas A. E. Al-Kuwari; Hadi M. Yassine; Hamad E. Al-Romaihi; Hamda Alromaihi; Heba A. Al-Khatib; Mashael A. Al-Bader; Mohammed Al-Thani; Muna A. S. Al-Maslamani; Oal Al-Jamal; Peter V. Coyle; Reham A. El-Kahlout. QBB: Tasneem Al-Hamad; Reham A. El-Kahlout. QBB: Tasneem Al-Hamad; Roberto Bertollini; Salih Al-Marri
EPI_ISL_2429128, EPI_ISL_2429130, EPI_ISL_2429131	Mubarak Al-Kabeer Hospital	Virology Unit, Department of Microbiology, Faculty of Medicine, Kuwait University	Anfal Al-Adwani; Hussain Safar; Nada Madi
EPI_ISL_2661466	US Air Force School of Aerospace Medicine	US Air Force School of Aerospace Medicine	Amanda Javorina; Anthony Fries; Carol Garrett; Clarise Starr; Elizabeth Macias; Jennifer Meyer; Sarah Purves; William Gruner

We gratefully acknowledge the following Authors from the Originating laboratories responsible for obtaining the specimens, as well as the Submitting laboratories where the genome data were generated and shared via GISAID, on which this research is based.

All Submitters of data may be contacted directly via www.gisaid.org

Authors are sorted alphabetically.

Acknowledgement EPI_SET Identifier: EPI_SET_20220314bu

Accession ID	Originating Laboratory	Submitting Laboratory	Authors
EPI_ISL_2833742, EPI_ISL_2833744, EPI_ISL_2833746, EPI_ISL_2833748, EPI_ISL_2833749, EPI_ISL_2833750, EPI_ISL_2833753, EPI_ISL_2833779, EPI_ISL_2833780, EPI_ISL_2833781, EPI_ISL_2833791, EPI_ISL_2833851, EPI_ISL_2833867, EPI_ISL_2833903, EPI_ISL_2833915, EPI_ISL_2833916			
see above	Adan Hospital	Kuwait Cancer Control Center	Mona Alateeqi; Shakir Bahzad
EPI_ISL_2833784	Adan Hospital	Kuwait Cancer control Center	Mona Alateeqi; Shakir Bahzad
EPI_ISL_2849763	Artic Network workflow-EPI2ME labs	King Khalid Bin Abdul Aziz street, South Surra, Kuwait City	Anfal Al-Adwani; Ebaa Al-Awadhi; Hussain Safar; Nada Madi
EPI_ISL_2622082, EPI_ISL_2622083, EPI_ISL_2622084, EPI_ISL_2622085, EPI_ISL_2622086, EPI_ISL_2622087, EPI_ISL_2663318, EPI_ISL_2663319, EPI_ISL_2663320, EPI_ISL_2663321, EPI_ISL_2663322, EPI_ISL_2663323, EPI_ISL_2663324, EPI_ISL_2812544, EPI_ISL_2812546, EPI_ISL_2812548, EPI_ISL_2812549, EPI_ISL_2812550, EPI_ISL_2812551, EPI_ISL_2812552, EPI_ISL_2812553, EPI_ISL_2812554, EPI_ISL_2812555, EPI_ISL_2812556, EPI_ISL_2812557, EPI_ISL_2812559, EPI_ISL_2812561, EPI_ISL_2812563, EPI_ISL_2812564, EPI_ISL_2812565, EPI_ISL_2812566, EPI_ISL_2812567, EPI_ISL_2812568, EPI_ISL_2812569, EPI_ISL_2812570, EPI_ISL_2812571, EPI_ISL_2812572, EPI_ISL_2812575			
see above	Communicable Disease Laboratory, Public Health Directorate	Communicable Disease Laboratory, Public Health Directorate	AlAbbas, Z.; AlHujairi, Z.; Almoamen, G.; Alwasti, H.; Marhoon, A.
EPI_ISL_2844844	Jaber Quarantine	Virology Lab, Jaber Al Ahmad Al Sabah Hospital	Adil Afridi; Dr. Ebaa Al-Awadhi; Dr. Zahrah Buhamad; Haroon Masih
EPI_ISL_2556117, EPI_ISL_2556119, EPI_ISL_2557251, EPI_ISL_2557309, EPI_ISL_2557459, EPI_ISL_2615399, EPI_ISL_2759093, EPI_ISL_2763624, EPI_ISL_2833731, EPI_ISL_2833733, EPI_ISL_2833734, EPI_ISL_2833735, EPI_ISL_2833738, EPI_ISL_2833739			
see above	MOH - Jaber Al-Ahmad Hospital (Innovation Research Laboratory)	MOH - Jaber Al-Ahmad Hospital (Innovation Research Laboratory)	Mohammad Alghounaim; Salman Al-Sabah
EPI_ISL_2603591, EPI_ISL_2603594, EPI_ISL_2603825, EPI_ISL_2603843, EPI_ISL_2603857, EPI_ISL_2604034, EPI_ISL_2604185, EPI_ISL_2604187, EPI_ISL_2604216, EPI_ISL_2604251, EPI_ISL_2680986, EPI_ISL_2776430, EPI_ISL_2835687, EPI_ISL_2838048, EPI_ISL_2838049, EPI_ISL_2846022, EPI_ISL_2846023, EPI_ISL_2846024, EPI_ISL_2847868, EPI_ISL_2851036			
see above	Ministry of Health, Jaber Al-Ahmad Hospital	Virology Unit, Department of Microbiology, Faculty of Medicine, Kuwait University	Anfal Al-Adwani; Ebaa Al-Awadhi; Hussain Safar; Nada Madi
EPI_ISL_2776369	Ministry of Health, Jaber Al-Ahmad Hospital	Virology Unit, Department of Microbiology, Faulty of Medicine, Kuwait University	Anfal Al-Adwani; Ebaa Al-Awadhi; Hussain Safar; Nada Madi
EPI_ISL_2835641, EPI_ISL_2835642, EPI_ISL_2835689	Ministry of Health-Jaber Al-Ahmad Hospital	Virology Unit, Department of Microbiology, Faculty of Medicine, Kuwait University	Anfal Al-Adwani; Ebaa Al-Awadhi; Ebaa Al-Awadi; Ebaa Al-awadhi; Hussain Safar; Nada Madi
EPI_ISL_2408255, EPI_ISL_2843013, EPI_ISL_2843014, EPI_ISL_2843021, EPI_ISL_2843022, EPI_ISL_2843023, EPI_ISL_2843024, EPI_ISL_2843025, EPI_ISL_2843026, EPI_ISL_2843027, EPI_ISL_2843028, EPI_ISL_2843030, EPI_ISL_2843031, EPI_ISL_2843032, EPI_ISL_2843033, EPI_ISL_2843034, EPI_ISL_2843035, EPI_ISL_2843036, EPI_ISL_2843037, EPI_ISL_2843038, EPI_ISL_2843039, EPI_ISL_2843041, EPI_ISL_2843042, EPI_ISL_2843043, EPI_ISL_2843044, EPI_ISL_2843045, EPI_ISL_2843046, EPI_ISL_2843047, EPI_ISL_2843187, EPI_ISL_2843198, EPI_ISL_2843199, EPI_ISL_2843207, EPI_ISL_2843208, EPI_ISL_2843209, EPI_ISL_2843210, EPI_ISL_2843211, EPI_ISL_2843212, EPI_ISL_2843213, EPI_ISL_2843214, EPI_ISL_2843215, EPI_ISL_2843216, EPI_ISL_2843217, EPI_ISL_2843218, EPI_ISL_2843219, EPI_ISL_2843220, EPI_ISL_2843221, EPI_ISL_2843222, EPI_ISL_2843223, EPI_ISL_2843224, EPI_ISL_2843225, EPI_ISL_2843226, EPI_ISL_2843227, EPI_ISL_2843228, EPI_ISL_2843230, EPI_ISL_2843231, EPI_ISL_2843232, EPI_ISL_2843234, EPI_ISL_2843235, EPI_ISL_2843236, EPI_ISL_2843238, EPI_ISL_2843239, EPI_ISL_2843240, EPI_ISL_2843241, EPI_ISL_2843242, EPI_ISL_2843243, EPI_ISL_2843244, EPI_ISL_2843245, EPI_ISL_2843246, EPI_ISL_2843247, EPI_ISL_2843248, EPI_ISL_2843249, EPI_ISL_2843250, EPI_ISL_2843251, EPI_ISL_2843252, EPI_ISL_2843253, EPI_ISL_2843254, EPI_ISL_2843255, EPI_ISL_2843256, EPI_ISL_2843257, EPI_ISL_2843258, EPI_ISL_2843259, EPI_ISL_2843260, EPI_ISL_2843261, EPI_ISL_2843262, EPI_ISL_2843263, EPI_ISL_2843264, EPI_ISL_2843265, EPI_ISL_2843266, EPI_ISL_2843267, EPI_ISL_2843268, EPI_ISL_2843269, EPI_ISL_2843270, EPI_ISL_2843271, EPI_ISL_2843272, EPI_ISL_2843273, EPI_ISL_2843274, EPI_ISL_2843275, EPI_ISL_2843276, EPI_ISL_2843277, EPI_ISL_2843278, EPI_ISL_2843279, EPI_ISL_2843280, EPI_ISL_2843281, EPI_ISL_2843282, EPI_ISL_2843283, EPI_ISL_2843284, EPI_ISL_2843285, EPI_ISL_2843286, EPI_ISL_2843287, EPI_ISL_2843288, EPI_ISL_2843289, EPI_ISL_2843290, EPI_ISL_2843303, EPI_ISL_2843304			
see above	Ministry of Public Health / Hamad Medical Corporation	Biomedical Research Center (BRC), Qatar University / Qatar Genome Project (QGP)	Asmaa A. Al-Thani. MOPH and HMC: Abdullatif Al-Khal; BRC: Fatiha M. Benslimane; Chadi Saad; Dana Al-Batesh; Dina Elgakhlab QGP: Fatima H. Al-Kuwari; Einas A. E. Al-Kuwari; Hadi M. Yassine; Hamad E. Al-Romaihi; Hamda Alromaihi; Heba A. Al-Khatib; Mashael A. Al-Bader; Mohammed Al-Thani; Muna A. S. Al-Maslamani; Oal Al-Jamal; Peter V. Coyle; Reham A. El-Kahlout. QBB: Tasneem Al-Hamad; Roberto Bertollini; Salih Al-Marri
EPI_ISL_2661491, EPI_ISL_2661492, EPI_ISL_2661493, EPI_ISL_2661494, EPI_ISL_2661495, EPI_ISL_2661496, EPI_ISL_2661497, EPI_ISL_2661499, EPI_ISL_2715358			
see above	US Air Force School of Aerospace Medicine	US Air Force School of Aerospace Medicine	Amanda Javorina; Anthony Fries; Carol Garrett; Clarise Starr; Cole Anderson; Elizabeth Macias; Fritz Castillo; Jennifer Meyer; Sarah Purves; William Gruner
