# Supplementary material for: Comparative Phylodynamics Reveals the Evolutionary History of SARS-CoV-2 Emerging Variants in the Arabian Peninsula
Source: Virus Evol. 2022 May 18:veac040. doi: 10.1093/ve/veac040 (PMC9129158; doi:10.1093/ve/veac040)
Supplement: veac040_Supp [file veac040_supp.zip › Suppl_Revised.pdf]

Comparative Phylodynamic Models Reveals the Evolutionary History of SARS-CoV-2 Emerging Variants in the Arabian Peninsula

Moh A. Alkhamis[1*☯], Nicholas M. Fountain-Jones[2☯], Mohammad M. Khajah[3], Mohammad Alghounaim[4, 5], Salman K. Al-Sabah[5,6]

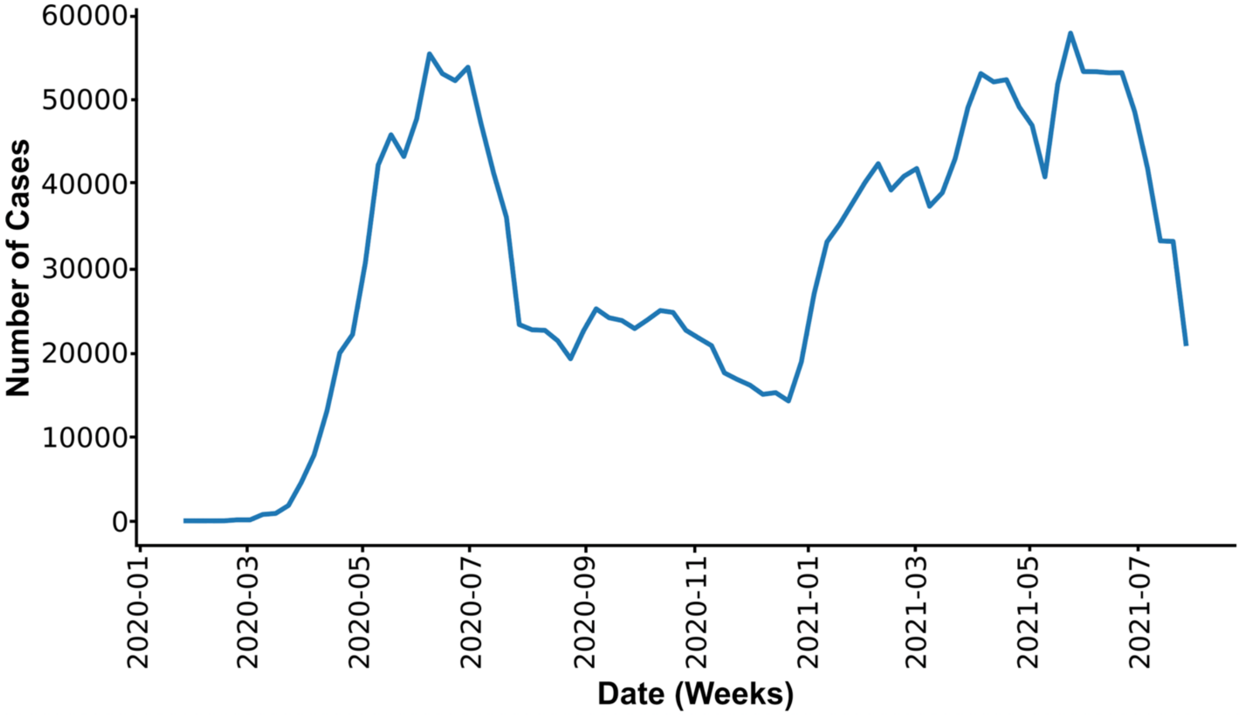

Figure S1. Weekly number of SARS-CoV-2 cases in the GCC countries between February 2020 and July 2021.

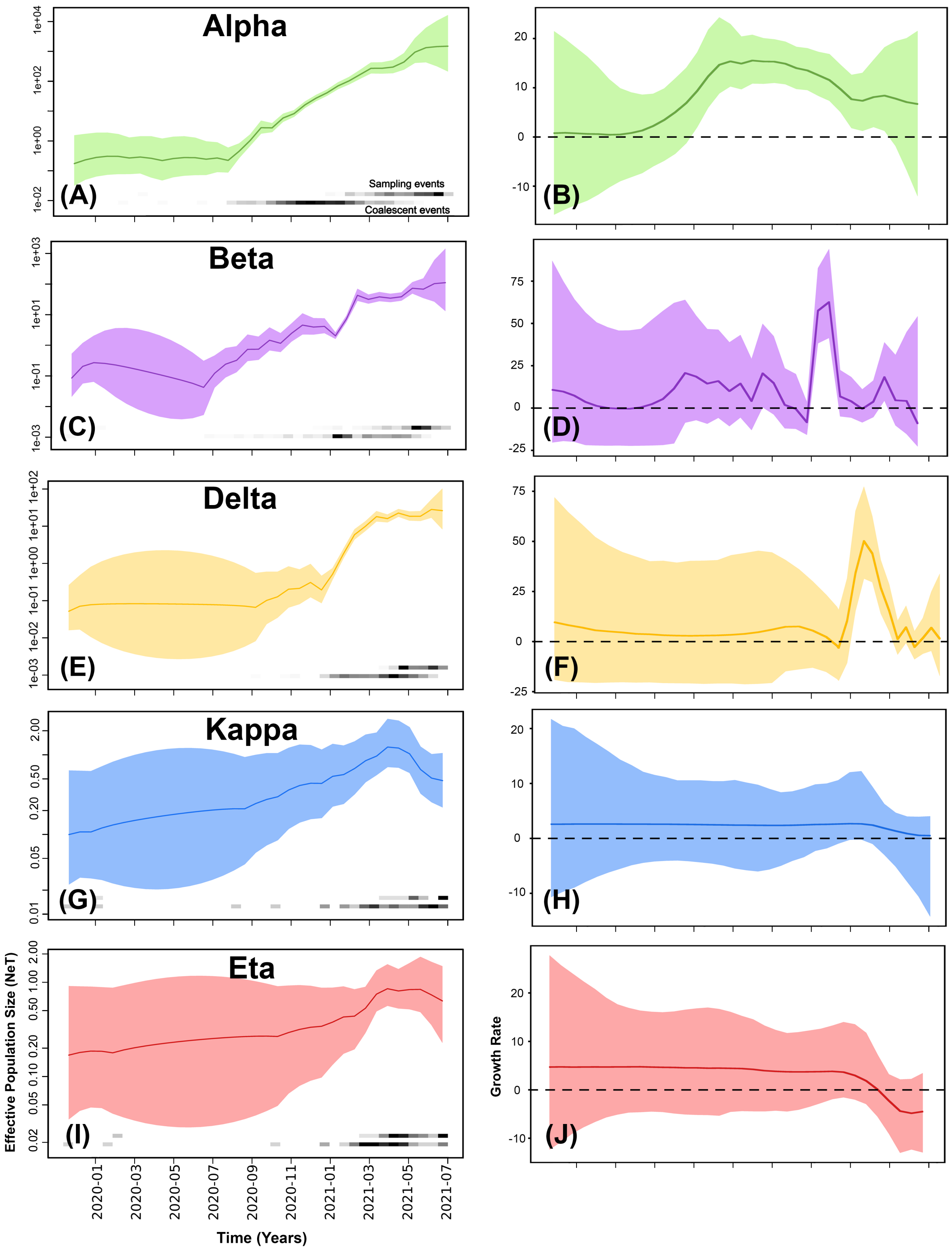

Figure S2. ***Phylodyn*** **skygrid plots and inferred growth rates through time of the selected SARS-CoV-2 variants in the Arabian Peninsula countries between November 16th 2020 and June 28th 2021.** The posterior median estimate is indicated by the dark colored line, and the light shaded areas correspond to the 95% high posterior density (HPD). (A-B) Alpha variant; (C-D) Beta variant; (E-F) represents Delta variant (G-H) represents Kappa variant; (I-J) represents Eta variant.

Table S1. Frequencies of the selected SARS-CoV-2 variants per GCC countries between November 16th 2020 and June 28th 2021.

	Alpha	Beta	Delta	Eta	Kappa	Total
Bahrain	40	8	28	-	3	79
Saudi Arabia	1	1	-	-	-	2
Kuwait	24	-	43	7	-	74
Oman	1	-	-	-	-	1
Qatar	106	250	149	1	2	508
United Arab Emirates	17	6	-	-	-	23
Total	189	265	220	8	5	686

Table S2. Frequencies of the selected SARS-CoV-2 variants per geographical region between November 16th 2020 and June 28th 2021.

Alpha			
Region/Year	2020	2021	Total
Africa	-	14	14
Asia	2	44	46
Europe	3	129	132
GCC	19	170	189
NorthAmerica	1	74	75
Oceania	1	28	29
SouthAmerica	-	28	28
Total	26	487	513
Beta			
	2020	2021	
Africa	3	46	49
Asia	-	27	27
Europe	-	16	16
GCC	5	254	259
NorthAmerica	-	8	8
Oceania	-	6	6
Grand Total	8	357	365
Delta			
	2020	2021	
Africa	-	29	29
Asia	-	65	65
Europe	-	32	32
GCC	-	218	218
NorthAmerica	-	11	11
Oceania	-	51	51
SouthAmerica	-	1	1
Total	-	407	407
Eta			
	2020	2021	
Africa	-	30	30
Asia	-	-	0
Europe	-	3	3
GCC	-	8	8
NorthAmerica	-	1	1
Oceania	-	1	1
Total	-	43	43

	Kappa		
	2020	2021	
Asia	-	9	9
Europe	-	1	1
GCC	-	5	5
Oceania	-	21	21
Total	-	36	36

Table S3. Bayes factor (BF) comparisons of the Alpha variant demographic phylodynamic models using path-sampling (PS) and stepping-stone (SS) methods. BFs based on the PS marginal likelihood estimates are on the upper off-diagonal of the table, while BFs based on SS marginal likelihood estimates are on the lower off-diagonal of thetable. Best fitting models have been boldfaced.

	**Bayes Factor**						
**Model**	UCED+CP	UCED+EG	UCED+EGx	UCED+SG	UCLN+CP	UCLN+EG	UCLN+SG
UCED[a]+CP[b]	—	17	-36	14	17	74	53
UCED+EG[c]	-4	—	-53	-3	0	57	36
**UCED+EGx[d]**	**50**	**54**	**—**	**50**	**53**	**110**	**89**
UCED+SG[f]	-45	-41	-95	—	3	60	39
UCLN[g]+CP	-15	-11	-65	30	—	57	36
UCLN+EG	-60	-56	-110	-15	-45	—	-21
UCLN+EGx	-122	-118	-172	-77	-107	-62	—
UCLN+SG	-12	-62	-62	33	3	48	110

[a]Uncorrelated relaxed clock with exponential distribution
[b]Constant population size coalescent model
[c]Expansion population size coalescent model
[d]Exponential population size coalescent model
[f]Bayesian skylgrid coalescent model
[g]Uncorrelated relaxed clock with log-normal distribution

Table S4. Bayes factor (BF) comparisons of the Delta variant demographic phylodynamic models using path-sampling (PS) and stepping-stone (SS) methods. BFs based on the PS marginal likelihood estimates are on the upper off-diagonal of the table, while BFs based on SS marginal likelihood estimates are on the lower off-diagonal of thetable. Best fitting models have been boldfaced.

	**Bayes Factor**						
**Model**	UCED+CP	UCED+EG	UCED+EGx	UCED+SG	UCLN+CP	UCLN+EG	UCLN+SG
UCED[a]+CP[b]	—	-103	-140	-87	-18	-137	-119
UCED+EG[c]	115	—	-37	16	85	-34	-16
**UCED+EGx[d]**	**120**	**5**	**—**	**53**	**122**	**3**	**21**
UCED+SG[f]	88	-27	-32	—	69	-50	-32
UCLN[g]+CP	18	-97	-102	-70	—	-119	-101
UCLN+EG	114	-1	-6	26	96	—	18
UCLN+EGx	85	-30	-35	-3	67	-29	—
UCLN+SG	34	-86	-86	-54	16	-80	-51

[a]Uncorrelated relaxed clock with exponential distribution
[b]Constant population size coalescent model
[c]Expansion population size coalescent model
[d]Exponential population size coalescent model
[f]Bayesian skylgrid coalescent model
[g]Uncorrelated relaxed clock with log-normal distribution
